# Pelvic Belt Effects on Pelvic Morphometry, Muscle Activity and Body Balance in Patients with Sacroiliac Joint Dysfunction

**DOI:** 10.1371/journal.pone.0116739

**Published:** 2015-03-17

**Authors:** Odette Soisson, Juliane Lube, Andresa Germano, Karl-Heinz Hammer, Christoph Josten, Freddy Sichting, Dirk Winkler, Thomas L. Milani, Niels Hammer

**Affiliations:** 1 Institute of Anatomy, University of Leipzig, Faculty of Medicine, Leipzig, Germany; 2 Institute of Applied Kinesiology, Technische Universität Chemnitz, Chemnitz, Germany; 3 Department of Trauma and Reconstructive Surgery, University of Leipzig, Faculty of Medicine, Leipzig, Germany; 4 Orthopedist, Osteologist and Pain Specialist, Kirchberg, Germany; 5 Department of Neurosurgery, University of Leipzig, Faculty of Medicine, Leipzig, Germany; The University of Queensland, AUSTRALIA

## Abstract

**Introduction:**

The sacroiliac joint (SIJ) is frequently involved in low back and pelvic girdle pain. However, morphometrical and functional characteristics related to SIJ pain are poorly defined. Pelvic belts represent one treatment option, but evidence still lacks as to their pain-reducing effects and the mechanisms involved. Addressing these two issues, this case-controlled study compares morphometric, functional and clinical data in SIJ patients and healthy controls and evaluates the effects of short-term pelvic belt application.

**Methods:**

Morphometric and functional data pertaining to pelvic belt effects were compared in 17 SIJ patients and 17 controls. Lumbar spine and pelvis morphometries were obtained from 3T magnetic resonance imaging. Functional electromyography data of pelvis and leg muscles and center of pressure excursions were measured in one-leg stance. The numerical rating scale was used to evaluate immediate pain-reducing effects.

**Results:**

Pelvic morphometry was largely unaltered in SIJ patients and also by pelvic belt application. The angle of lumbar lateral flexion was significantly larger in SIJ patients without belt application. Muscle activity and center of pressure were unaffected by SIJ pain or by belt application in one-leg stance. Nine of 17 patients reported decreased pain intensities under moderate belt application, four reported no change and four reported increased pain intensity. For the entire population investigated here, this qualitative description was not confirmed on a statistical significant level.

**Discussion:**

Minute changes were observed in the alignment of the lumbar spine in the frontal plane in SIJ patients. The potential pain-decreasing effects of pelvic belts could not be attributed to altered muscle activity, pelvic morphometry or body balance in a static short-term application. Long-term belt effects will therefore be of prospective interest.

## Introduction

The sacroiliac joint (SIJ) is frequently involved in painful conditions of the pelvis and the lower extremity [1–9]. The anatomy of the SIJ and presumably its biomechanics predispose it to become involved in low back pain [10]. However, the SIJ is difficult to identify as the source of low back pain [1–3,5,8,11]. Specific pain provocation tests are used to identify the SIJ as the primary source of pain [12], but mostly with poor inter-rater reliability [13]. Injecting local anesthetics into the SIJ cavity is the gold standard for confirming this diagnosis [1,8]. At the same time injections can provide a temporary pain relief [13]. However, the clinical and radiological findings related to SIJ syndromes are poorly defined and the underlying pathomechanisms are subject of speculation [14,15]. Despite the high incidence of SIJ pain, only sparse data can be found on the association of SIJ pain and pelvic or lower limb anatomy [16,17] and muscle activity [18–23]. It is therefore of interest to study the relations of SIJ pain to joint morphology and muscle activation patterns in order to optimize the treatment of SIJ patients.

According to the recommendations of the international association for the study of pain (IASP), SIJ pain should primarily be managed conservatively [24]. Overall SIJ related intervention rates have increased by more than 300% in the last decade with an increasing ratio of surgical interventions [25]. Surgical interventions to the SIJ lack in beneficial effects [26], are significantly more expensive [27] and have higher complication rates than the non-surgical treatment [28,29]. Their cost-effectiveness is questionable [30]. As a consequence, the surgical management of SIJ pain should be limited to therapy-refractory cases [31]. Pelvic belts are one cost-effective option in the non-surgical treatment of SIJ pain [32]. Pelvic belts are assumed to increase neuromotor performance [23,33] and *form* and *force closure* [34]. However, there is limited evidence that the pelvic belts reduce SIJ mobility and there are few patient-controlled studies to specify their effects on the pelvis [23,32,34]. Taking into account the prescription frequencies of pelvic belts in Europe, there is a clear lack of scientific benchmark data for these devices [34]. Our study aims to address this lack of scientific data from a biomedical point of view.

This study compares pelvic morphometry of patients with SIJ pain to healthy controls in a static position. Muscle activation patterns of pelvis and lower limb muscles and center of pressure data (COP) of the foot in one-leg stance were also subject of this investigation. Additionally, in both groups, the effects of pelvic belts were investigated on pelvic morphometry, muscle activation and on COP. It was hypothesized that pelvic morphometry, muscle activation and COP are different in SIJ patients, as compared to healthy controls. It was furthermore hypothesized that pelvic belts are capable of normalizing altered pelvic morphometry, muscle activation patterns and/or COP.

## Material and Methods

**Fig 1:**
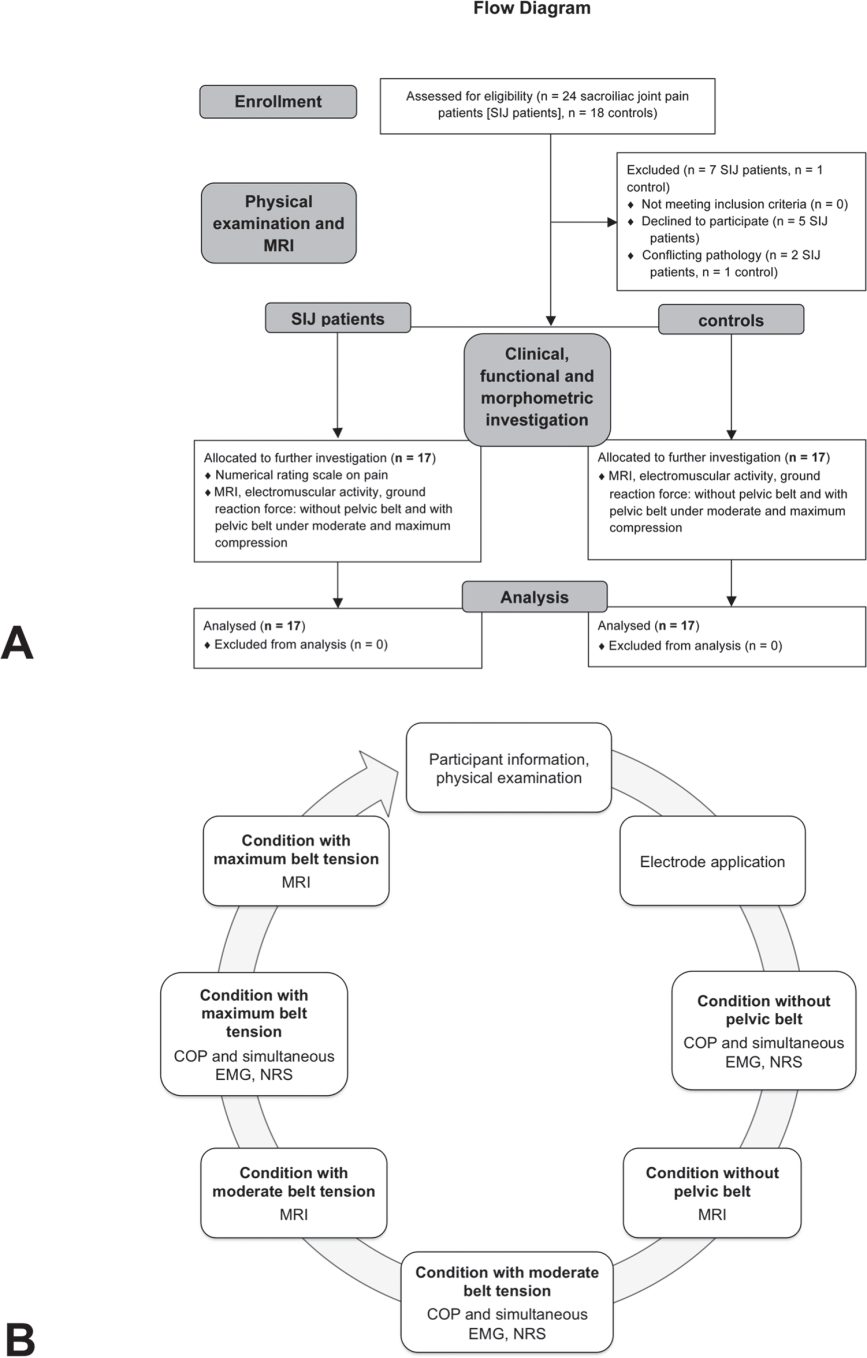
Summary of the experimental setup.

This study was approved by the ethics committee of the University of Leipzig (number 063-11-07032011) and registered at ClinicalTrials.gov (NCT02027038). The ethics committee approved the clinical trial protocol shown in Fig. 1A and 1B before the trial began. Written consent was ratified from all participants. The principal investigator (N.H.) delayed the registration of the study until data acquisition was completed for confidentiality reasons concerning the study methods. The authors confirm that all ongoing and related trials for this intervention are registered.

### General information

The study population consisted of 24 patients suffering from chronic pain arising from the SIJ joint, enrolled between August 2011 and December 2012. The patients were sent from orthopedic outpatients’ clinics. SIJ patients were selected according to the following criteria: pain duration of at least twelve weeks, at least three positive SIJ pain provocation tests [1,8] and if intra-articular injection of local anesthetics provided temporal relief of the symptoms of at least 75% [35–37]. The control group consisted of 18 age-matched controls without any history of musculoskeletal disorders. All participants were interviewed regarding their current health condition and their medical history and underwent a second physical examination. None of the participants were taking any medication that could affect body balance response or analgesics on the investigation day. A flow diagram [38] and the study protocol are given in Fig. 1A and 1B, respectively, according to the STROBE guidelines [39]. The exclusion criteria are listed in Table 1. The raw data are presented in the S1 Data.

**Table 1:** Exclusion criteria of patients with sacroiliac joint pain and healthy controls.

SIJ patients	Healthy controls
fractures or muscular disorders	
metallic implants (e.g. pacemakers or endoprostheses)	
previous episodes of claustrophobia	
somatoform disorders	
Pregnancy	
degenerative joint diseases except sacroiliac joint pain	any kind of degenerative or inflammatory joint disease
inflammatory joint diseases	complaints of the low back and/or history of low back pain

The effects of pelvic belts (SacroLoc, Bauerfeind AG, Zeulenroda-Triebes, Germany) were determined in three levels of application intensity: no pelvic belt application, moderate pelvic belt tension and the maximal tolerable pelvic belt tension. The magnitude of moderate tension was adapted by the participants as being suitable for everyday situations, according to the manufacturer. The maximum tolerable tension was defined as the highest applicable belt tautness without perceiving pelvic belt-related pain or discomfort in the standing position. Each pelvic belt was exclusively used for one participant. Four different clothing sizes were available, being adapted depending on the pelvic circumference of each participant. Magnetic resonance imaging (MRI), electromyography (EMG) and stance analyses were performed in each step and in all participants, as shown in Fig. 1.

### Numerical Rating Scale (NRS)

All patients were surveyed regarding their pain intensity with the 11-point NRS. They were surveyed without applying the pelvic belt and with the pelvic belt under moderate and under maximum tension. The survey was performed immediately after the participants underwent each trial including the respective MRI scan, EMG and stance analyses of the respective level of application, averaging one hour each.

### MRI

Three Tesla MRI data (MAGNETOM TRIO, Siemens AG, Erlangen, Germany) of the lumbar spine and pelvis were recorded in all participants to investigate pelvic belt-related effects on the morphometry of the pelvic ring and the SIJ. With the exception of the lumbar spine scanned only without pelvic belt application, all scans were recorded without a pelvic belt and with a pelvic belt under moderate and maximum tension. Additionally, MRI scans were obtained from all participants to rule out inflammatory causes of SIJ pain or extra-articular pathologies that potentially cause comparable symptoms. The lumbar spine, the pelvic ring and both SIJ were investigated in T1-weighted, T2-weighted, Turbo-Inversion Recovery-Magnitude and Double Echo Steady State sequences in the lying position [17,40,41].

### Comparison of pelvic and SIJ morphometry related to pelvic belt effects in SIJ patients and controls

Two investigators (O.S., N.H.) performed the morphometric evaluation with the Voxim software (JoCoMed, Chemnitz, Germany). Prior to the measurements, the MRI data of all participants were rendered anonymous and blinded to which group the participants were in. Additionally, both investigators were blinded to their previous measurements. Anatomical landmarks were defined at the lumbar spine, the pelvic ring and the SIJ in a patient- or control-specific coordinate system. The placement points of the anatomical landmarks were defined as follows:

- Center of the respective anatomical structure,
- within the plane of the patient- or control-specific coordinate system that was (most) perpendicular to the landmark,
- in the MRI section that included the anatomical landmark to maximum extent, and (if this applied to more than one section)
- within the MRI section most distally from the region of interest.

Based on these data, distances and angles between the anatomical landmarks were computed as shown below. Every anatomical landmark was determined twice in each dataset in a random order.

#### Lumbar spine

**Fig 2:**
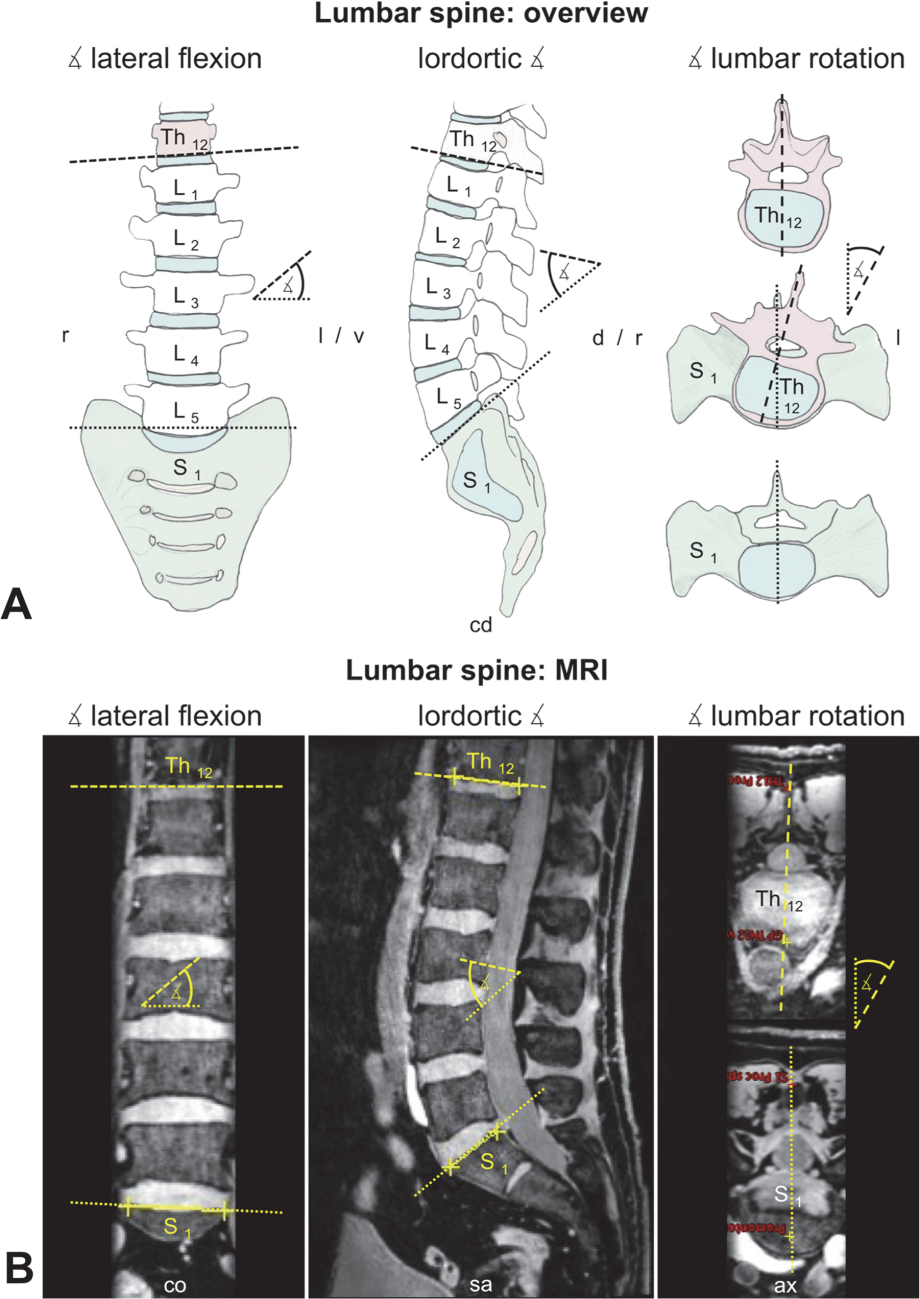
Morphometrical measurements. On basis of 3 Tesla magnetic resonance imaging, angles and spatial relations were compared at the lumbar spine. ASIS = anterior superior iliac spine, ax = axial plane, co = coronal plane, cd = caudal, cr = cranial, d = dorsal, l = left, MRI = magnetic resonance imaging, PSIS = posterior superior iliac spine, r = right, S _1,2,etc._ = first (second, etc.) sacral vertebral body, sa = sagittal plane, Th _12_ = twelfth thoracic vertebral body, v = ventral, ∡ = angle.

Each angle was determined in the respective anatomical plane, as done in standard X-rays of the lumbar spine (Fig. 2A,B). The lumbar lordotic angle was defined by the intersection of two lines in the median sagittal plane. One line represented the lower twelfth thoracic vertebra (Th12) surface and the other line represented the upper first sacral vertebra (S1) surface (S1 Fig.). The angle of lumbar rotation was defined by the intersection of two lines from Th12 and S1 in the horizontal plane. Here, each line connected the anterior center of the vertebral corpus with the respective spinous process (S2 Fig.). The angle of lateral flexion was defined by the intersection of a line at the lower Th12 surface with a line at the upper S1 surface in the frontal plane. Both lines consisted of two points set at both lateral borders of each respective vertebral body (S3 Fig.).

#### Pelvis

**Fig 3:**
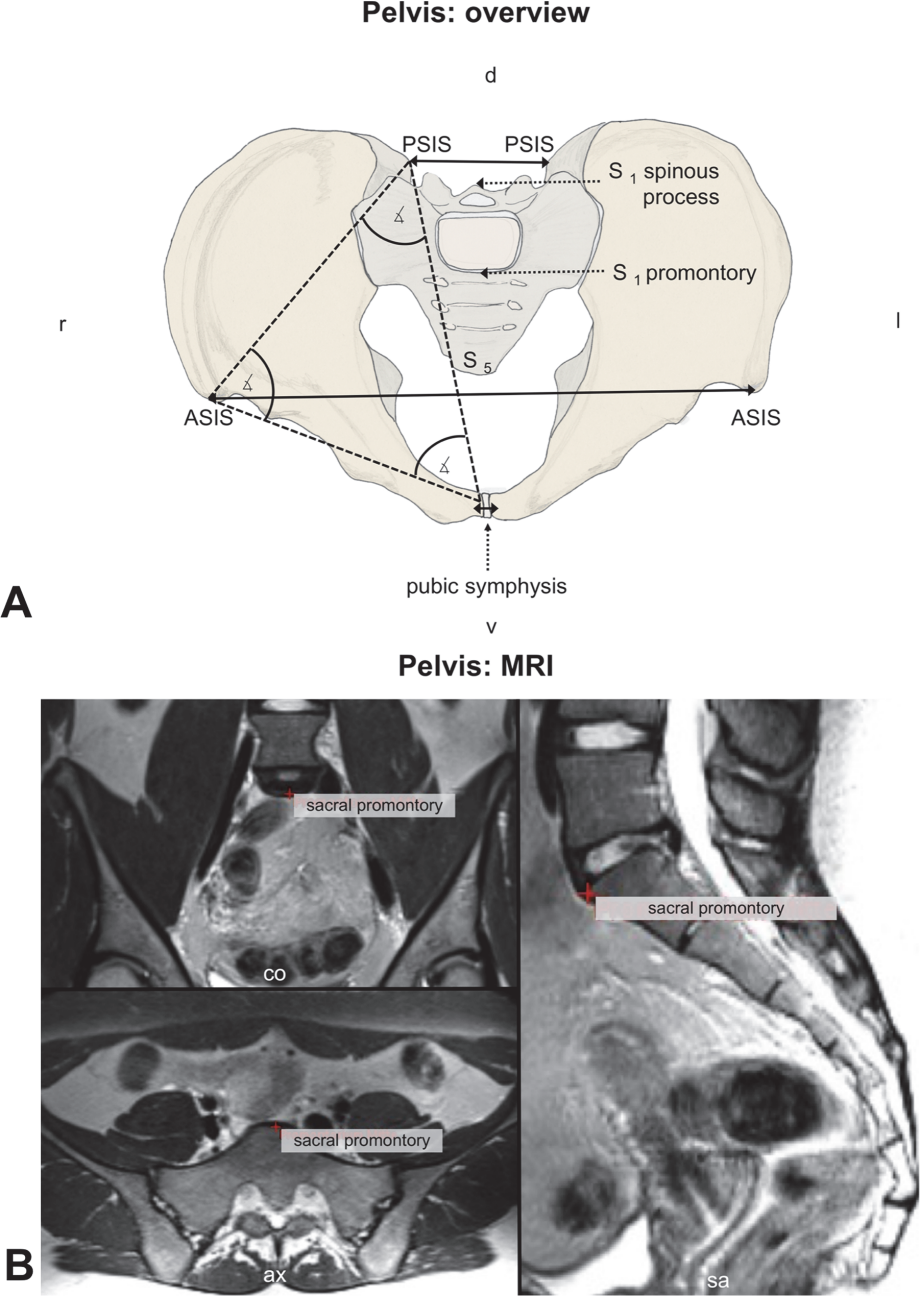
Morphometrical measurements. On basis of 3 Tesla magnetic resonance imaging, angles and spatial relations were compared at the pelvis. ASIS = anterior superior iliac spine, ax = axial plane, co = coronal plane, cd = caudal, cr = cranial, d = dorsal, l = left, MRI = magnetic resonance imaging, PSIS = posterior superior iliac spine, r = right, S _1,2,etc._ = first (second, etc.) sacral vertebral body, sa = sagittal plane, Th _12_ = twelfth thoracic vertebral body, v = ventral, ∡ = angle.

The following anatomical landmarks were defined bilaterally at the pelvic ring: the anterior superior iliac spine (ASIS), posterior superior iliac spine (PSIS) and the center of the inferior ramus (symphysis) of the pubic bone (Fig. 3A,B; S4 Fig.). At the sacrum, the ventral center of the first sacral vertebral body (S1 promontory), the S1 spinous process and the lower ventral edge of the fifth sacral vertebral body (S5) were selected (S2 Fig.). To analyze pelvic belt-related compression effects, the distances between the ASIS, PSIS and both parts of the symphysis were computed. For determining motions occurring within each of the pelvic bones related to pelvic belt application, the ASIS-PSIS, ASIS-symphysis and PSIS-symphysis distances were measured bilaterally. Additionally, the rotation and the translation of the sacrum were analyzed relative to each of the pelvic bones. Here, the distances and the angles from three vectors were compared: promontory-S5 and ASIS-promontory left as well as promontory-S5 and ASIS-promontory right.

#### SIJ

**Fig 4:**
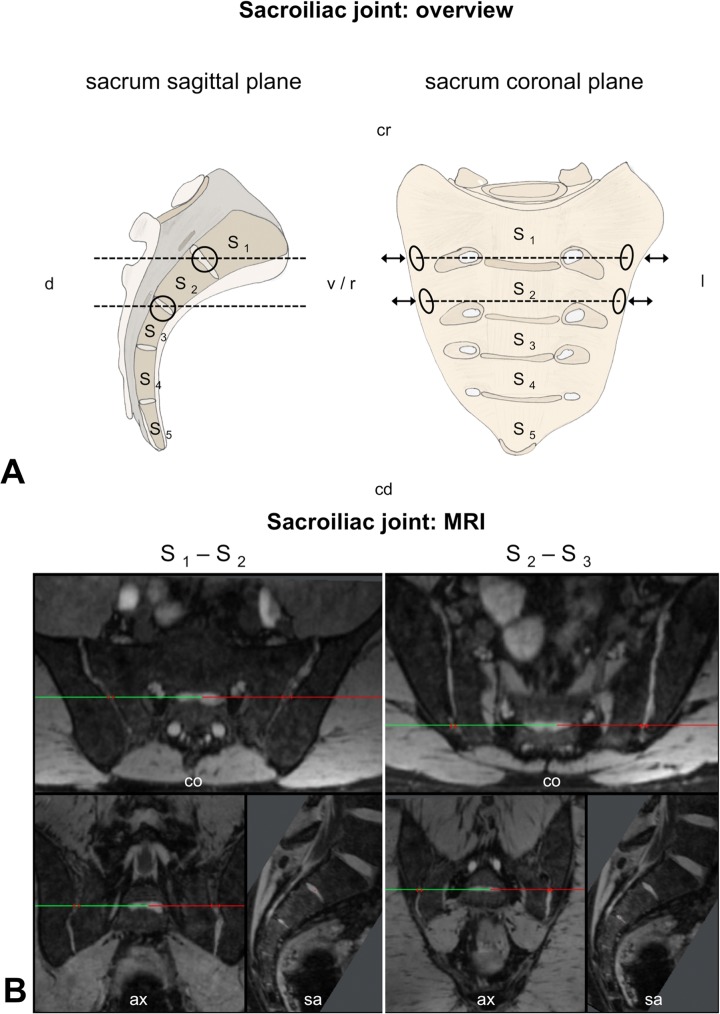
Morphometrical measurements. On basis of 3 Tesla magnetic resonance imaging, angles and spatial relations were compared at the sacroiliac joint. ASIS = anterior superior iliac spine, ax = axial plane, co = coronal plane, cd = caudal, cr = cranial, d = dorsal, l = left, MRI = magnetic resonance imaging, PSIS = posterior superior iliac spine, r = right, S _1,2,etc._ = first (second, etc.) sacral vertebral body, sa = sagittal plane, Th _12_ = twelfth thoracic vertebral body, v = ventral, ∡ = angle

The distances between the cartilage of the ilium and the sacrum were measured bilaterally at the S1-S2 and S2-S3 disk level to depict compression effects at the auricular surface of the SIJ (Fig. 4A,B; S5 Fig.).

### EMG and stance analysis

**Fig 5:**
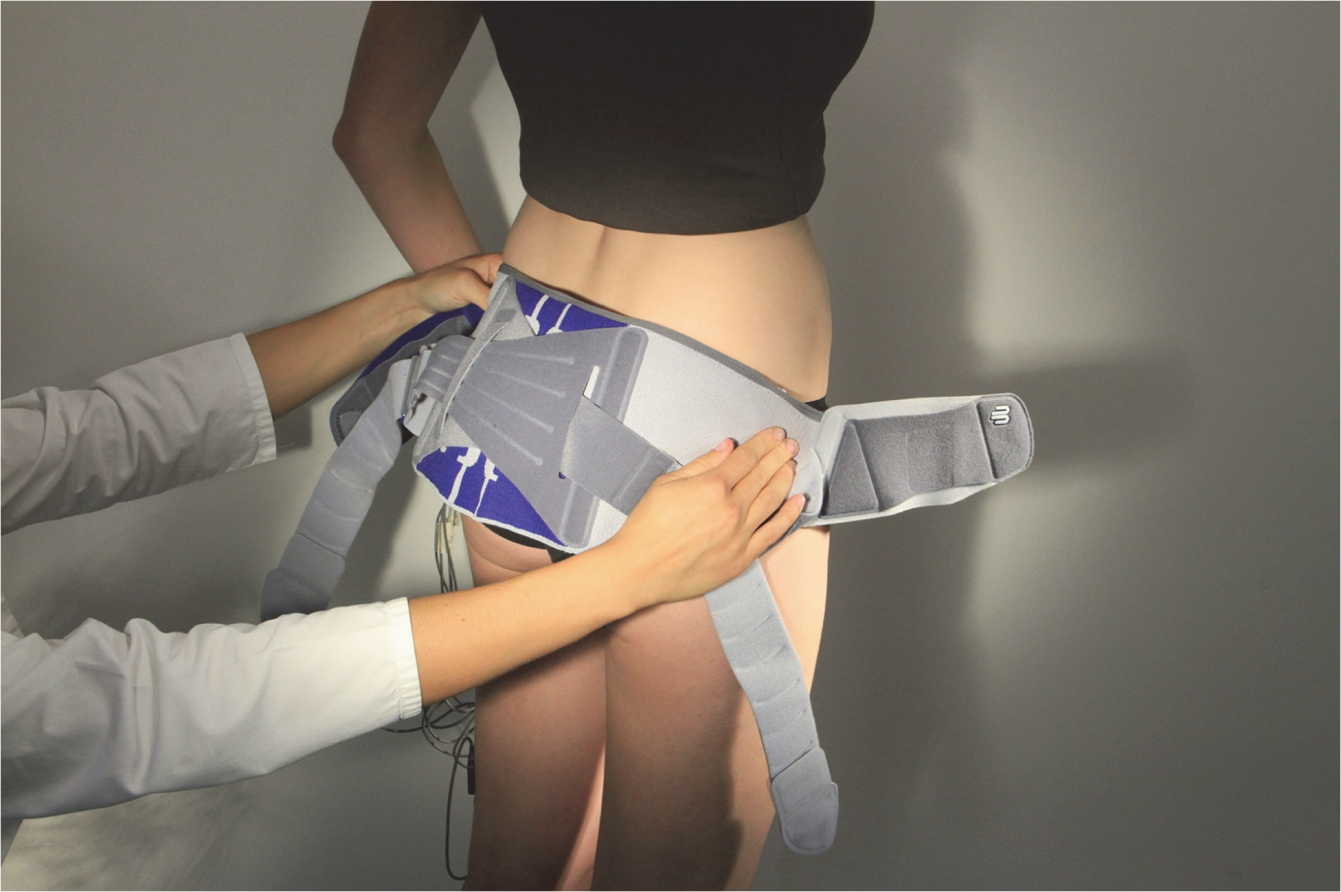
Pelvic belt application. A SacroLoc belt (Bauerfeind AG, Zeulenroda-Triebes, Germany) is applied to a female volunteer under moderate tension, as recommended by the manufacturer.

Surface EMG were recorded simultaneously with COP measurements in one-leg stance for all pelvic belt conditions (Fig. 5, S6 and S7 Figs.). The placement of the sensors was performed according to SENIAM recommendations [42]. The adductor magnus, the biceps femoris (long head), the gastrocnemius (medial head), the gluteus maximus, the medial vastus, the rectus femoris, the tensor fasciae latae muscles and the tibialis anterior were recorded from the dominant leg. The dominant leg was identified as proposed by Tate and coworkers [43]. The reference electrode was placed at the lateral malleolus of the respective foot. For EMG-data acquisition the Bagnoli-8 EMG system (Delsys Inc., Boston, MA, USA) was used. EMG signals measured at a frequency of 1000 Hz, pre-amplified and band-pass filtered (20–450 Hz; Butterworth 4th order). Integrated EMG (iEMG) was calculated using MatLab software (version 8.5, National Instruments, Austin, TX, USA).

Body balance analyses were performed with an AFDM 1.5 measuring plate (zebris Medical GmbH, Isny, Germany) at a frequency of 100 Hz. All participants were asked to stand upright, look straightforward for the duration of data recording, lasting ten seconds. Data from the side (more) affected by SIJ pain regarding the patient group and the dominant leg regarding the controls were recorded [44]. COP excursions were computed using the WinFDM software (version 2, zebris Medical GmbH, Isny, Germany).

### Statistical analysis

Statistical computations were performed using R software (The R Foundation for Statistical Computing, Vienna, Austria), Excel 2010 (Microsoft Cooperation, Redmond, WA, USA) and SPSS version 20.0 (Armonk, NY, USA). Normal distribution was determined with the Kolmogorow-Smirnow test. The Student’s t test for independent samples and the Mann-Whitney-U test were applied to evaluate differences in the baseline characteristics of the participants including age, gender, body height and weight, the pain-reducing effect (Δ NRS) and the angle of lumbar lateral flexion. Within-group comparison on the different tension conditions of the pelvic belt was performed using Levene’s test to assess the equality of variances, proceeded by a repeated measures univariate ANOVA for more than two paired samples and post-hoc analyses with the Bonferroni, Fisher's Least Significant Difference and Tukey's range test if applicable. Between-group comparison of patients and controls was performed with the Friedman’s tests with posthoc analyses with the Wilcoxon signed-rank test if applicable. Bland-Altman plots were used to determine the reliability of the measurements in the MRI scans [45,46]. *P*-values of 5% or less were considered being statistically significant.

## Results

The data of 17 SIJ patients (10 ♀, 7 ♂) and healthy controls (11 ♀, 6 ♂) were included in this prospective study. Seven patients and one control were excluded for the following reasons: claustrophobia in MRI (5 patients) and conflicting pathology after physical examination (1 patient, 1 control). One patient was excluded after the interpretation of the MRI records due to a gynecological pathology. Patient mean age was 45.1 ± 11.0 years (mean ± standard deviation) and mean body mass index (BMI) was 24.9 ± 3.4 kg/m^2^. Controls had a mean age of 43.7 ± 19.9 years and a mean BMI of 24.2 ± 3.9 kg/m^2^. Mean age, body height, weight and BMI did not vary significantly between patients and controls. All patients suffered from moderate or severe SIJ pain (NRS or visual analogue scale ≥ 3; [47]). Further baseline data are given in Table 2.

**Table 2:** Baseline data of SIJ patients.

Patient no.		Age	Sex	Height	Weight	BMI	Dominant	Pain duration	NRS			Δ NRS	
									no	moderate	maximum	no belt -	no belt -
		[years]		[cm]	[kg]		leg	[months]	belt	tension		moderate	maximum
1		39	f	1.60	50.9	19.9	r	46	4	6	4	2	0
2		45	f	1.64	69.3	25.8	r	82	8	8	8	0	0
3		49	m	1.80	79.9	24.7	r	130	3	4	5	1	3
4		71	m	1.75	86.5	28.2	l	170	2	1	5	−1	3
5		40	f	1.78	91.3	28.8	r	36	3	5	4	2	1
6		33	f	1.53	58.0	24.8	r	12	4	3	4	1	1
7		39	f	1.78	64.2	20.3	l	54	3	2	3	−1	0
8		46	m	1.87	85.3	24.4	r	54	4	4	3	−1	−1
9		55	m	1.68	88.4	31.3	l	18	6	0	4	−6	−2
10		51	f	1.67	61.1	21.9	l	4	6	5	6	−1	1
11		32	m	1.74	66.9	22.1	r	19	2	0	0	−2	−2
12		46	m	1.73	73.4	24.5	r	10	6	4	3	−2	−3
13		60	f	1.63	68.8	25.9	r	60	3	3	5	0	2
14		42	f	1.75	73.3	23.9	l	120	2	2	3	0	1
15		27	m	1.84	71.6	21.1	r	4	2	2	1	0	−1
16		38	f	1.64	83.1	30.9	r	12	6	5	6	−1	1
17		54	f	1.68	71.8	25.4	r	96	5	4	4	−1	−1
**MV ± SD**	**patients**	**45.1 ± 11.0**		**1.71 ± 0.1**	**73.2 ± 11.3**	**24.9 ± 3.4**		**54.5 ± 48.2**	**4.0 ± 1.8**	**3.4 ± 2.1**	**4.0 ± 1.9**	**−0.6 ± 1.7**	**0.0 ± 1.6**
**Median**									**4.0**	**3.5**	**4.0**	**−0.5**	**0.0**
**MV ± SD**	**controls**	**43.7 ± 19.9**		**1.68 ± 0.1**	**68.1 ± 9.3**	**24.2 ± 3.9**							

Age, gender, body height and weight, body mass index (BMI), the side of the dominant leg (l = left, r = right), pain duration and the results of the numerical rating scale (NRS) survey are given. MV = Mean value, SD = standard deviation, Δ NRS = pain-reducing effect

### Short-term application of pelvic belts was related to non-significant alterations in pain intensity

**Fig 6:**
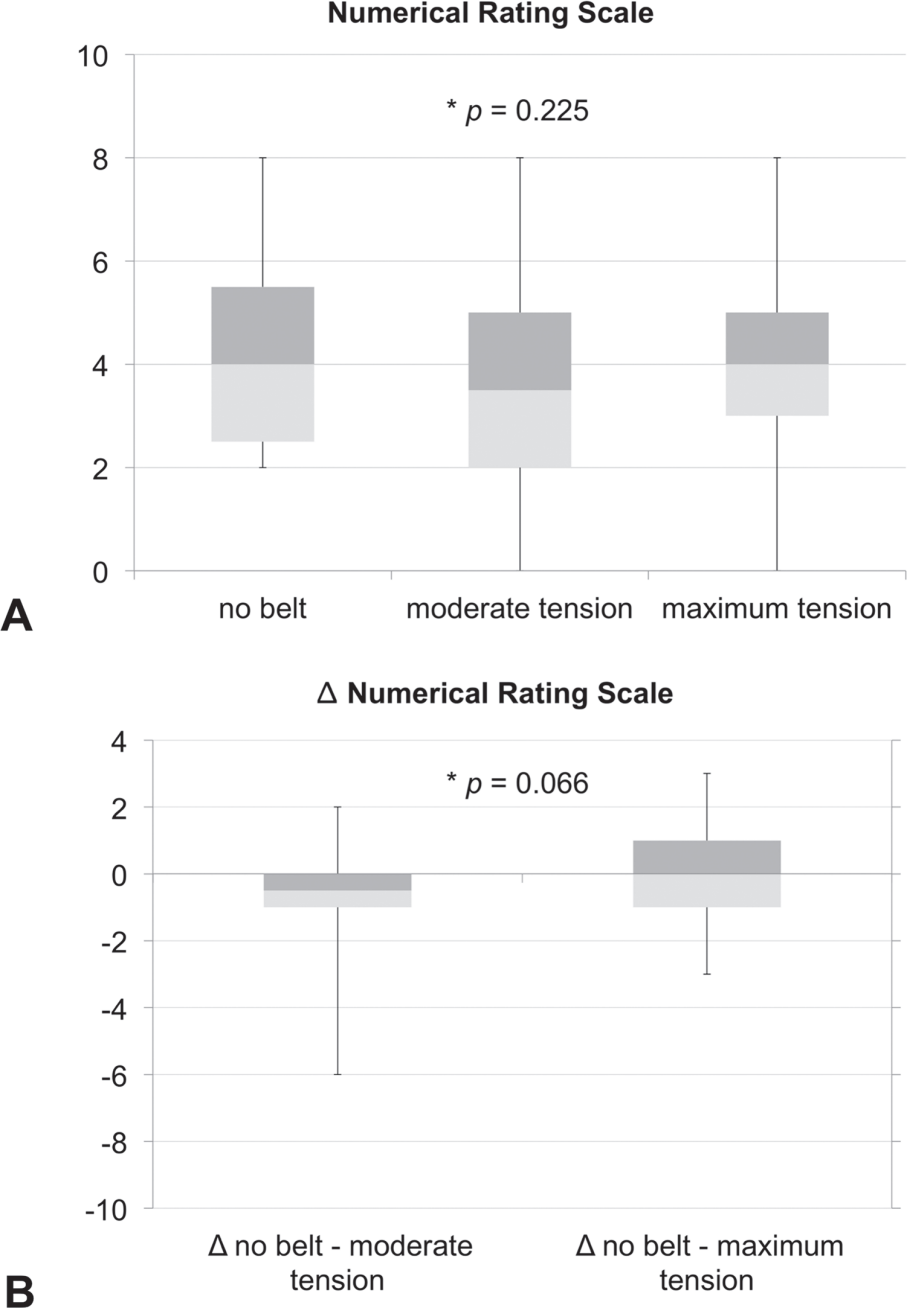
11-point Numerical rating scale (NRS) data on pain intensity. Fig. 6A: Non-significantly altered pain intensity was observed in sacroiliac joint pain patients with belt application under moderate tension, while maximum tension slightly increased pain intensity. Fig. 6B: Comparison to the condition without pelvic belt (Δ NRS) revealed that moderate tension tended to change pain intensity more effectively than maximum tension in patients with sacroiliac joint pain on a non-significant level.

The NRS was 4.0 ± 1.8 on the investigation day without using a pelvic belt but after physical examination (Fig. 6A; Table 2; median = 4.0). Pelvic belt application under moderate tension changed the pain intensity non-significantly to 3.4 ± 2.1 (median = 3.5), as compared to the condition without belt and with the belt under maximum tension (*p* = 0.23, Friedman test). Pelvic belt application under maximum tension was related to a pain intensity of 4.0 ± 1.9 (median = 4.0; *p* = 0.23; Friedman test). Nine of 17 patients reported decreased pain intensity under moderate tension, whereas four patients reported no change and four patients reported increased pain intensity (Table 2). Under maximum tension, six patients reported decreased pain intensity, three reported no change and eight patients reported increased pain intensity.

Compared to the condition without a belt (Δ NRS), applying pelvic belt under moderate tension tended to non-significantly decrease SIJ-related pain intensity to more extent than maximum tension with -0.6 ± 1.7 (median = -0.5) vs. 0.0 ± 1.6 (median = 0.0; *p* = 0.07, Student’s t test for independent samples; Fig. 6B; Table 2).

### Spatial relations of the anatomical landmarks are largely unaffected by SIJ pain except for the angle of lateral flexion at the lumbar spine and spatial relations are largely unaltered by pelvic belt application

Without wearing the pelvic belt, the angle of lateral flexion at the lumbar spine displayed slight but significantly higher values in SIJ patients (4.73 ± 2.72°), as compared to the healthy controls (2.81 ± 2.17°; *p* = 0.02). The mean values and standard deviations of the data from the lumbar spine, the pelvis and the SIJ are given in Table 3. Mean differences (bias) and limits of agreement were low, indicating reliability in repeated measurements (S1 Table). No further differences on a significant different level were determined in the measurements of the pelves and the SIJs, comparing the landmark positions derived from the MRI data of SIJ patients and controls under the different states of pelvic belt tension (S2 Table).

**Table 3:** Between-group comparison of lumbar spine, pelvis and sacroiliac joint (SIJ) morphometries of SIJ patients and controls with and without pelvic belt application.

		No belt		Moderate tension		Maximum tension		*p*
		patients	controls	patients	controls	patients	controls	
**Lumbar spine**								
angle	lateral flexion	4.7 ± 2.7	2.8 ± 2.2	not recorded		not recorded		*0.02*
[°]	lumbar rotation	2.0 ± 0.9	3.2 ± 3.0					*0.48*
	lumbar lordotic	52.1 ± 8.9	56.6 ± 7.0					*0.39*
**Pelvis**								
distance	ASIS left—ASIS right	223.7 ± 20.6	232.5 ± 19.7	224.3 ± 21.6	232.8 ± 20.2	224.6 ± 20.5	232.6 ± 19.5	*1.00*
[mm]	PSIS left—PSIS right	90.7 ± 10.9	91.3 ± 10.5	90.6 ± 10.1	91.3 ± 10.2	90.3 ± 10.9	91.0 ± 10.6	*1.00*
	symphysis left—right	14.6 ± 19.5	11.6 ± 2.5	11.1 ± 2.7	11.2 ± 3.2	10.7 ± 2.2	10.9 ± 2.4	*0.69*
	ASIS—PSIS left	162.4 ± 6.5	159.9 ± 7.9	161.9 ± 6.3	160.0 ± 8.3	162.0 ± 6.2	159.4 ± 8.0	*0.99*
	ASIS—PSIS right	163.4 ± 6.6	160.9 ± 8.0	163.0 ± 5.7	160.6 ± 7.8	163.2 ± 6.0	160.6 ± 7.6	*1.00*
	ASIS—symphysis left	153.2 ± 8.3	155.1 ± 11.3	153.2 ± 8.5	155.4 ± 11.4	153.5 ± 7.6	155.7 ± 11.9	*1.00*
	ASIS—symphysis right	151.9 ± 9.1	153.1 ± 10.9	152.6 ± 8.9	153.9 ± 11.5	152.3 ± 8.9	154.0 ± 11.3	*1.00*
	PSIS—symphysis left	178.6 ± 9.7	176.7 ± 10.0	177.9 ± 10.0	177.3 ± 9.9	178.4 ± 9.1	176.9 ± 10.3	*0.97*
	PSIS—symphysis right	173.9 ± 14.2	177.3 ± 8.8	177.5 ± 9.4	176.7 ± 8.6	177.2 ± 9.5	176.7 ± 8.9	*0.65*
	symphysis—S5	58.3 ± 0.1	58.3 ± 0.1	57.2 ± 2.0	57.6 ± 2.3	58.3 ± 0.1	58.3 ± 0.1	*0.58*
angle	promontory—ASIS left	59.2 ± 4.4	58.4 ± 4.7	57.1 ± 6.6	57.8 ± 3.8	58.2 ± 5.2	58.2 ± 5.1	*0.82*
[°]	promontory—ASIS right	124.1 ± 13.5	128.7 ± 9.3	128.2 ± 15.3	129.2 ± 9.6	126.6 ± 12.0	128.7 ± 9.9	*0.84*
**SIJ**								
distance	S1—S2 left	6.1 ± 2.8	5.2 ± 2.6	6.8 ± 3.4	5.8 ± 3.0	6.2 ± 2.9	5.4 ± 2.8	*0.99*
[mm]	S1—S2 right	5.9 ± 3.0	4.8 ± 2.6	5.9 ± 2.9	5.0 ± 2.5	6.1 ± 2.4	4.9 ± 2.6	*0.97*
	S2—S3 left	4.2 ± 1.5	3.8 ± 1.9	4.5 ± 1.9	3.9 ± 1.5	4.0 ± 1.4	4.3 ± 2.6	*0.64*
	S2—S3 right	4.4 ± 1.9	3.6 ± 1.4	3.8 ± 1.6	3.8 ± 2.0	4.1 ± 1.6	3.2 ± 1.2	*0.53*

The angle of lumbar lateral flexion was significantly larger in SIJ patients, as compared to healthy controls (p = 0.02). Values are given as mean values, standard deviations and p-values. ASIS = anterior superior iliac spine, PSIS = posterior superior iliac spine, S1, S2, S3 = first, second, third sacral vertebral body

### Pelvis and lower extremity muscle activity was largely unaltered by SIJ pain or pelvic belt application in one-leg stance

Both increases and decreases were found for the muscle activities of the gluteus maximus, tensor fasciae latae, rectus femoris, adductor magnus, biceps femoris, medial vastus, gastrocnemius and tibialis anterior muscle (Tables 4 and 5). However, these changes did not reach a statistically significant level between SIJ patients and controls under the different states of pelvic belt tension (Table 4). Also, the within-group comparison in the SIJ patients and controls showed no significant increases or decreases in the muscle activities due to pelvic belt application in one-leg stance, as indicated by the integral (Table 5).

**Table 4:** Between-group comparison of surface electromyography (EMG) and ground reaction force data of patients with sacroiliac joint (SIJ) pain and controls with and without pelvic belt application.

	No belt		Moderate tension		Maximum tension		*p*
	patients	controls	patients	controls	patients	controls	
**Analyzed muscles integral [μVs]**							
gluteus maximus	421.3 ± 218.2	404.2 ± 221.3	481.7 ± 235.3	475.5 ± 245.7	561.9 ± 251.5	411.0 ± 213.8	*0.41*
tensor fasciae latae	1011.4 ± 710.1	1223.2 ± 744.8	1081.0 ± 908.7	1494.7 ± 1301.2	1096.0 ± 736.2	1416.8 ± 1017.7	*0.92*
rectus femoris	696.4 ± 627.2	377.7 ± 236.6	609.9 ± 606.0	402.4 ± 247.0	770.6 ± 666.0	372.2 ± 234.8	*0.73*
adductor magnus	243.1 ± 130.9	240.4 ± 138.3	270.4 ± 155.3	256.5 ± 121.2	319.1 ± 260.4	232.3 ± 151.7	*0.57*
biceps femoris	908.4 ± 496.7	495.6 ± 303.1	775.4 ± 427.8	490.7 ± 268.0	815.0 ± 501.2	424.2 ± 235.6	*0.79*
medial vastus	849.7 ± 811.8	578.7 ± 503.8	862.8 ± 788.8	637.8 ± 684.9	812.0 ± 748.1	581.2 ± 653.7	*0.99*
medial gastrocnemius	2234.2 ± 1701.0	1726.7 ± 676.5	2338.1 ± 1457.6	1850.0 ± 830.8	2119.9 ± 1570.9	1663.6 ± 573.3	*1.00*
tibialis anterior	2544.8 ± 1699.2	2349.1 ± 2032.4	2373.1 ± 1648.4	2315.3 ± 2096.9	2211.1 ± 1335.4	2280.9 ± 2347.5	*0.96*
**Ground reaction force**							
COP [mm]	330.1 ± 97.1	342.5 ± 136.1	320.1 ± 92.8	328.5 ± 126.6	337.2 ± 101.9	305.3 ± 101.3	*0.66*

No significant differences in the muscle activities of SIJ patients and healthy controls were observed. Also, no differences were observed in the center of pressure (COP) data, derived from the pressure distribution data in one-leg stance. Values are given as mean values, standard deviation and p-values.

**Table 5:** Within-group comparison of surface electromyography (EMG) ground reaction force data of patients with sacroiliac joint (SIJ) pain and controls with and without pelvic belt application.

*p-values*	No belt: moderate tension: maximum tension	
	Patients	controls
**Analyzed muscles [integral]**		
gluteus maximus	*0.06*	*0.25*
tensor fasciae latae	*0.61*	*0.14*
rectus femoris	*0.93*	*0.14*
adductor magnus	*0.40*	*0.61*
biceps femoris	*0.14*	*0.61*
medial vastus	*0.58*	*0.14*
medial gastrocnemius	*0.93*	*0.94*
tibialis anterior	*0.40*	*0.22*
**Ground reaction force**		
COP, side of SIJ pain	*0.79*	-
COP, standing leg	-	*0.20*

The p-values refer to the data given in Table 4. No significant differences were observed in the muscle activities and in the center of pressure (COP) data.

### COP force data is similar in the SIJ patients and controls and largely unaffected by pelvic belt application

The comparison of the center of pressure data in one-leg stance did not reveal any changes related to pelvic belt application or differences between SIJ patients and controls. The mean values, standard deviations and *p*-values are listed in Tables 4 and 5.

## Discussion

Our study aimed to identify morphometric changes in the pelvis and SIJ in the sense of *form closure* and functional differences in the pelvic and lower limb muscles along with COP for measuring the extent of *force closure* [23,34] from a biomedical point of view. Three Tesla MRI, EMG and COP were utilized for this purpose, comparing patients with chronic SIJ pain to healthy control subjects. Furthermore, our study aimed at investigating the effects of pelvic belts in SIJ patients and controls and to determine acute pain-relieving effects using the NRS scale. This is the first study to perform an encompassing comparison between SIJ patients and age-matched controls as well as on the effects of pelvic belts on *form* and *force closure* [18]. Previous studies focused on the clinical tests with related muscle forces [48] or on SIJ laxity [33].

### Pelvic belt-mediated pain-relieving effects were unlikely mediated in a short-term application in SIJ patients—does long-term use decrease pain intensity?

Pelvic belt application caused a slight and non-significant change in pain intensity when the pelvic belt was applied under moderate tension, as compared to the condition without a belt (Fig. 6A; Table 2). When the pelvic belt was applied under maximum tension, the pain intensity was non-significantly higher, as compared to the condition without the pelvic belt application. A small majority of the patients with SIJ pain (9/17; Table 2) benefited from pelvic belt application under moderate tension in a short-term setting, but only to a limited extent, as indicated by the Δ NRS data (Fig. 6B; Table 2). These data suggest that pelvic belts are potentially capable of decreasing SIJ-related pain to some extent or of maintaining decreased pain intensity related to other interventions even in a short-term application. The pain-reducing effect tended to be better under moderate than under maximum tension (Fig. 6B; Table 2), indicating that belt application under tension may rather be recommended on the basis of the missing effects in the MRI, EMG and stance analysis data. The mean NRS change over all SIJ patients related to short-term pelvic belt application was -0.5 and therefore smaller than the NRS decreases recommended by the group of Childs et al. [49] and Salaffi and coworkers [50] to show a clinically meaningful therapeutic effect. However, the minimally clinically important difference was exceeded in nine of the seventeen patients, as indicated by Δ NRS decreases of -1 and -6 under moderate tension [50], indicating that there are potential responders and non-responders of belt application even in a short-term setting (Table 2). This phenomenon might be due to the variability of sources of low back pain discussed in literature [1–3,5,9,11,14,24,34,35,96].

It needs to be emphasized that the condition under maximum tension was just below the tension required for perceiving pelvic belt-related pain by the participants. Moreover, the MRI scans of the study protocol forced all participants to lay motionless in a supine position for 30 minutes or more even with the belt under maximum tension.

Greater declines in pain intensity were reported with SIJ manipulation techniques [51,52], when treating the SIJ surgically by joint fusion [53–61], by denervation techniques [28,62,63] or by the injection of local anesthetics [1,2,29,60,62,64–71] in a long-term follow up. However, surgical interventions are accompanied by adverse complications such as nerve lesions or postoperative wound infections, and there is limited evidence for the long-term outcome of these procedures [28,29]. No such complications have ever been reported for pelvic belt application. Pelvic belts may even protect from the pain-increasing effects of increased intraabdominal pressure onto the SIJ [48]. Therefore, surgical interventions should be limited to therapy-refractory cases [54]. Also, the health expenditures related to pelvic belt application in the treatment of SIJ dysfunction far smaller than surgical intervention, underlining the appropriateness of pelvic belts as a treatment of SIJ afflictions [27]. Less than 50% of the patients return to work after surgical SIJ interventions [29]. It is therefore highly relevant to gain insight into pelvic belt effects on *form and force closure* related to the pain-decreasing effects of pelvic belts. Beyond the minute and non-significant immediate pain-reducing effects related to pelvic belt application, longer-term follow up data are necessary in patients and in a more dynamic setting, e.g. when walking.

### The horizontal alignment of the lumbar spine was altered in SIJ dysfunction and pelvic belts had negligible compression effects on the pelvis

In order to evaluate differences in pelvic morphometry, anatomical landmarks at the bony pelvis and the SIJ were compared between SIJ patients and controls using data obtained from MRI. Furthermore, this method was used for comparing changes in pelvic morphometry related to the application of pelvic belts. One parameter differed significantly between patients and controls: the angle of lumbar lateral flexion (Fig. 2; Table 3). This finding indicated that there could be an association between the alignment of the lumbar spine in the frontal plane and SIJ pain, as shown recently [72]. An increased angle of lateral flexion might be related to shear stress at the lumbosacral transition [73] or vice versa an increased angle of lateral flexion might be a compensatory reaction to counterbalance SIJ pathologies contra-laterally. These data are in accordance with previous findings showing that SIJ dysfunction is closely related to lumbar spine impairments [72,74] and may be attributed to increased lumbopelvic muscle activity to the effect that pelvic motion decreases [75]. Previous reports stated that the sagittal alignment of the spine might be related to SIJ pain [72,76]. Conclusively, also its alignment in the frontal plane could be attributed to SIJ pain. However, the differences in the angle of lateral flexion of the lumbar spine were minute and they were found on both the more and the less affected SIJ side. Here, the question arises whether such small differences are clinically relevant or measurable at all. Concerning morphometric changes within the pelvic ring and the SIJ between patients and controls, no further differences were found on a statistically significant level. It was impossible to visualize alterations of pelvic morphometry related to the application of pelvic belts in our setting with MRI. It can therefore be concluded that the changes in pelvic morphometry related the application of pelvic belts are minute, confirming studies on human subjects [77,78] and computer simulations with the SIJ [79,80]. Consequently, our data indicated that 3 Tesla MRI in the given setup was insufficient to visualize compression effects to pelvic belts or that compressive effects were below the accuracy of the MRI measurements. Vice versa, pelvic belts may possibly mediate the pain relieving effects not exclusively via compression, but also by selectively recruiting the stabilizing musculature or other neurophysiological pathways [34,81,82]. However, no acute pain-relieving effects on a significant level could be shown in our present study.

A couple of limitations need to be addressed in the context of our approach using MRI. Firstly, MRI is an inferior method for visualizing minute changes of bone morphometry, which is a clear advantage of computed tomography or plain film radiography [83]. These imaging modalities visualize the pelvic bones more clearly and with less statistical variance [84,85]. In spite of the limitations of MRI as an imaging modality, it was chosen due to its clinical availability, the minimal health risks, the potential of MRI to visualize the SIJ morphology and to exclude inflammatory or extra-articular causes of SIJ pain [2,16,17,41,86–88]. Secondly, all scans were performed in the lying position, which may have caused a counter nutation [89] and altered muscle activity with effects on the SIJ, as compared to the standing posture. Lumbar spine measurements were missing in the conditions under moderate and maximum tension to keep the measurement times acceptable for the patients. This data will be of interest in future studies incorporating open MRI to determine whether an altered lumbar spine alignment is also found in the standing posture and whether pelvic belts are capable of altering the alignment of the lumbar spine. Thirdly, measurement errors [90] may have been introduced by our morphometrical setup with the given anatomic landmarks. The Bland and Altman plots presented here support this suspicion (S1 and S2 Tables). Further testing for agreement on basis of the Bland and Altman plots [45] revealed that the limits of agreement (95% confidence limits) for repeated measurements were larger than the calculated mean differences (bias) between SIJ patients and controls in each state of belt application or comparing the different states of pelvic belt tension within the patients or controls (S1 Table). This implied that the predictable measurement error by repeated measurements could be larger than the actual mean difference between patients and controls or certain pelvic belt interventions.

### Short-term pelvic belt application did not alter pelvis and lower extremity muscle activity in SIJ patients and healthy controls in one-leg stance

Comparison of the EMG data obtained from the pelvic and limb muscles revealed that there were no significant differences in the muscle activity of SIJ patients and healthy controls (Table 4). Furthermore, the muscle activity remained largely unaltered by the application of pelvic belts in a short-term application and in one-leg stance (Table 5). Shadmehr et al. [22] determined changes in the recruitment of the biceps femoris of SIJ patients and controls. Jung et al. [20] showed that the biceps femoris activity is altered by the application of pelvic belts. The biceps femoris exerts torsional forces on the SIJ, as it originates at the tuberosity of the ischium and as it is closely interspersed with the sacrotuberous ligament [91–93], a potential pain generator of the SIJ [8,94–97]. It was hypothesized that an increased biceps femoris activity reduces SIJ motion as a compensatory effect in the sense of *force closure* [19,69,98]. Pelvic belt application decreased the activity of the biceps femoris in patients [20], indicating that such application increases *form and/or force closure*. However, these effects could not be confirmed by our data in a short-term setting and in one-leg stance.

Previously, gluteus maximus activity was shown to either increase [21] or decrease [20] with the application of the pelvic belt. The gluteus maximus is also known to exert torsional and shear forces on the SIJ [92]. It originates at the sacrum, the ischium, and it is closely blended with the erector spinae and to the sacrotuberous ligament [76,99]. The gluteus maximus inserts mainly at the femur [99] and the iliotibial tract [100], making it very likely that the gluteus maximus additionally causes compressive stress at the SIJ and shear forces to the lower lumbar segments [101], which may be related to the lumbar lateral flexion. Gluteal weakness has been reported in the context of SIJ pain [69]. These findings could however not be confirmed by our data (Tables 4 and 5). Shadmehr et al. recorded decreased activity of the gluteus maximus in SIJ patients when performing the active straight leg raise in the lying position without using a pelvic belt [22]. Therefore an increased gluteus maximus activity might indicate partial recovery of gluteal strength [69]. Though rectus femoris activity was always larger in patients than in controls in our data, this difference failed to reach a significant level. In the synopsis of the muscle activity data of SIJ patients and controls in one-leg stance, surface EMG may not give reliable results to confirm or exclude the diagnosis of SIJ dysfunction and the clinical significance of these findings needs to be questioned in a short-term application setting of pelvic belts. Further research in a dynamic walking setting may therefore help to gain more insight into alterations of muscle activity related to SIJ dysfunction and belt effects.

Our EMG data were limited by the fact that only the dominant legs were investigated in the healthy controls and that the (more) symptomatic side was investigated in the SIJ patients. Though the side of the affected SIJ in the patient group was the dominant one in the majority of the cases (10/17; 59%), this simplification impacted the results of the EMG data. Measuring the muscles on both sides would have solved this issue. However, we wanted to keep the data comparable between patients and controls and only had a limited amount of EMG equipment available for this study. Furthermore, most clinical tests in the context of SIJ pain may be regarded as “static”, e.g. the active straight leg raise test [11,18–20,22] or the Storck test [11,102], which was our justification for this static setup in the one-leg stance.

The EMG data was subject to the following restrictions: Skin preparation and repetitive electrode positioning might have caused different conditions in the participants [103]. Also, neighboring muscles might have interfered with the EMG signals of the respective muscles of interest in the sense of cross talk [104–106]. These limitations might have caused the variations in the EMG [104,105] in addition to inter-individual variations.

### Body balance was largely unaltered in SIJ patients or by pelvic belts in one-leg stance

Based on the assumption of differences in muscle activity, we further hypothesized that the body balance might differ in patients and in controls and that pelvic belts affect the center of foot pressure or weight distribution when standing. Joseph and coworkers [107] and by Mendez et al. [108] proposed that there might be a difference in body balance in the sense of an impaired forward-feed activation of the foot. The lack of any significant differences in COP when standing in our study might be associated with the self-locking characteristics of the SIJ when being loaded in the one-leg stance as investigated here (Tables 4 and 5). An increased *form* and *force closure* might have optimized the force transition to the foot, being reflected by similar COP as a compensatory mechanism [108]. A small sample size and a short measuring interval limited our COP. Furthermore, comparison of the more symptomatic side in patients to the dominant side in the controls may have affected the results. Parreira et al. proposed longer intervals to record the center of pressure, which was however impossible with the SIJ patients in the given setup [109]. The lack of further morphometric differences and similar COP excursions of the foot between SIJ patients and controls indicated that even without therapeutic intervention the musculoligamentous apparatus might partly be capable of compensating imbalances in *form and force closure*, resulting in minute changes of pelvic and lower extremity biomechanics in SIJ patients.

### Summary and conclusions

Patients with pain arising from the SIJ were shown to have a minutely increased angle of lateral flexion at the lumbar spine. There was a lack of evidence that compressive forces were exerted on the SIJ or pelvis via pelvic belts. Muscle activity was largely unaltered in patients with SIJ dysfunction in one-leg stance. A majority of SIJ patients reported decreased pain intensity with a pelvic belt applied under moderate tension. However, the mean pain-altering effects averaged over all patients were minute in a short-term setting and on a non-significant level. Muscle activity was largely unaltered in patients with SIJ dysfunction in one-leg stance, as compared to healthy controls. There were no significant differences in the COP excursions between SIJ patients and controls. The given study focused on morphometric und functional differences between SIJ patients and controls as well as immediate effects of pelvic belts in a static setting. Being well aware of the multi-facet pathogenesis of SIJ dysfunction that well incorporates bio-psychosocial dimensions, this study focused on the biomedical point of view exclusively. However, this simplification helped determine somatic effects of SIJ dysfunction, which was also strengthened by the strict exclusion criteria of the SIJ patients in our study. In the static one-leg stance setting presented here, neither 3 Tesla MRI, nor surface electromyography of the given muscles or COP analyses served as a tool to differentiate patients with SIJ-related pain from control participants. Further research is necessary to establish differences between SIJ patients and controls in a long-term and dynamic setting, and to elucidate the dynamic effects of pelvic belts, addressing their potential in improving the health-related quality of life in SIJ patients.

## Supporting Information

S1 Data Raw numerical rating scale on pain (NRS) data along with the baseline data of the patients with chronic sacroiliac joint dysfunction (PDF)

S1 Fig Pictorial summary of the anatomical landmarks that were used to compute pelvic belt-induced motions at the pelvis and sacroiliac joint and morphometric differences between patients with sacroiliac-joint pain and healthy controls. Each of the landmarks was checked in all standard anatomical planes. The lumbar lordotic angle was defined by the intersection of two lines in the median sagittal plane. One line represented the lower twelfth thoracic vertebra surface (baseplate) and the other line represented the upper first sacral vertebra surface (endplate). Each line consisted of a landmark at the ventral and the dorsal edge at the respective vertebra.
(TIF)

S2 Fig Pictorial summary of the anatomical landmarks that were used to compute pelvic belt-induced motions at the pelvis and sacroiliac joint and morphometric differences between patients with sacroiliac-joint pain and healthy controls. Each of the landmarks was checked in all standard anatomical planes. The angle of lumbar rotation was defined by the intersection of two lines from the baseplate of the twelfth thoracic vertebra and the endplate of the first sacral vertebra in the horizontal plane. Each line consisted of a landmark at the ventral edge and the spinous process of the respective vertebra.
(TIF)

S3 Fig Pictorial summary of the anatomical landmarks that were used to compute pelvic belt-induced motions at the pelvis and sacroiliac joint and morphometric differences between patients with sacroiliac-joint pain and healthy controls. Each of the landmarks was checked in all standard anatomical planes. The angle of lateral flexion was defined by the intersection of a line at the twelfth thoracic vertebra baseplate with a line at the first sacral vertebra endplate in the frontal plane. Both lines consisted of two points set at the lateral edges of each respective vertebral body.
(TIF)

S4 Fig Pictorial summary of the anatomical landmarks that were used to compute pelvic belt-induced motions at the pelvis and sacroiliac joint and morphometric differences between patients with sacroiliac-joint pain and healthy controls. Each of the landmarks was checked in all standard anatomical planes. The anterior and superior iliac spine and the pubic symphysis are marked at the pelvis.
(TIF)

S5 Fig Pictorial summary of the anatomical landmarks that were used to compute pelvic belt-induced motions at the pelvis and sacroiliac joint and morphometric differences between patients with sacroiliac-joint pain and healthy controls. Each of the landmarks was checked in all standard anatomical planes. A reference plane was created with three landmarks: the ventral edge of the first sacral vertebra endplate (promontory), the first sacral vertebra spinous process and the caudal tip of the fifth sacral vertebra. Two lines indicated in green perpendicular to this plane were set at the S1-S2 and S2-S3 disk level. At this line, landmarks were set on the sacral and the iliac side of the sacroiliac joint, measuring the cartilage and joint cavity to full extent.
(TIF)

S6 Fig Surface electromyography data were recorded from all patients with sacroiliac joint pain and controls in one-leg stance without applying a pelvic belt, under moderate and maximum tolerable tension. The electrodes were positioned on the adductor magnus, the rectus femoris, the medial vastus, the tibialis anterior.
(TIF)

S7 Fig Surface electromyography data were recorded from all patients with sacroiliac joint pain and controls in one-leg stance without applying a pelvic belt, under moderate and maximum tolerable tension. The electrodes were positioned on the gluteus maximus, the tensor fasciae latae, the biceps femoris and the (medial) gastrocnemius.
(TIF)

S1 Table Comparison of MRI-based measurement agreement of lumbar spine, pelvis and sacroiliac joint (SIJ) morphometries on basis of Bland-Altman plots without pelvic belt, under moderate and maximum tension. Means and bias of the Bland-Altman analyses are given. ASIS = anterior superior iliac spine, PSIS = posterior superior iliac spine, S1 (2, 3) = first (second, third) sacral vertebral body. *A:* Between-group comparison of SIJ patients and healthy controls. *B:* Within-group comparison of the SIJ patient group and the control group measurements from two raters
(DOCX)

S2 Table Within-group comparison of MRI-based morphometry data of patients with sacroiliac joint (SIJ) pain and controls with and without pelvic belt application. The *p*-values refer to the data given in Table 3. No significant differences were observed for the different conditions of pelvic belt tension.
(DOCX)
