# Supplementary material for: Pelvic Belt Effects on Pelvic Morphometry, Muscle Activity and Body Balance in Patients with Sacroiliac Joint Dysfunction
Source: PLoS One. 2015 Mar 17;10(3):e0116739. doi: 10.1371/journal.pone.0116739 (PMC4364533; doi:10.1371/journal.pone.0116739)
Supplement: S1 Data — (PDF) [file pone.0116739.s001.pdf]

Patienten-ID: 2011 - 10 - 17 - 17 P

Fragebogen Teil 1

Liebe Patientin, lieber Patient,

Wir bitten Sie alle nachfolgenden Fragen wahrheitsgemäß zu beantworten.
Bitte kreuzen Sie für jede Frage, sofern nicht anders angegeben, ein Kästchen an!
Bearbeiten Sie zuerst die linke, dann die rechte Spalte.
Bei Fragen stehen wir jederzeit zur Verfügung.

Persönliche Angaben

1. Geburtsdatum: 30.12.71 Alter: 39
2. Geschlecht: ☐ männlich ☒ weiblich
3. Größe: 160
4. Gewicht: 50,9 5. BMI: 19,88
6. Beinigkeit: ☐ links ☒ rechts
7. Beckenumfang: 84,5 cm

Orthesengröße: ☒ 1 ☐ 2 ☐ 3 ☐ 4

8. Haben sie das Gefühl, dass Sie sich unsicher beim Gehen fühlen, bzw. häufig stürzen?

☐ ja ☒ nein

Angaben zur Schmerzsymptomatik

9. Seit wann bestehen die Schmerzen?

☐ weniger als 1 Monat ☐ 1 Monat bis ½ Jahr
☐ ½ bis 1 Jahr ☒ 1 bis 2 Jahre
☐ 2 bis 5 Jahre ☐ mehr als 5 Jahre

10. Bitte zeichnen Sie ein, an welchen Körperstellen Ihre Schmerzen auftreten

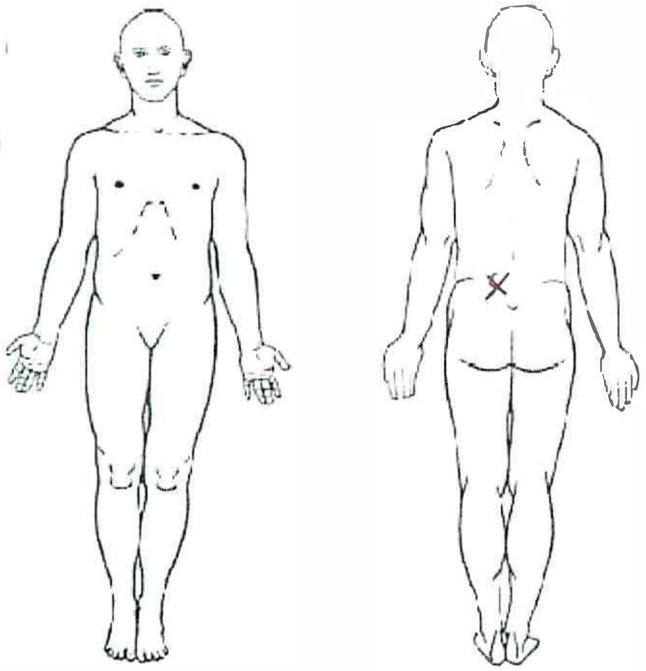

11. Bitte versuchen Sie die Schmerzqualität genauer zu beschreiben, das heißt, wie Sie die Schmerzen typischerweise empfinden. ***Mehrfachnennung ist möglich.***

☐ dumpf ☐ drückend ☐ pochend
☐ klopfend ☐ stechend ☒ ziehend
☐ heiß ☒ brennend ☐ unerträglich

Bitte geben Sie nun die Stärke Ihrer Schmerzen an. Kreuzen Sie dafür auf den Linien die Zahlen an, die Ihrer Schmerzempfindung am nächsten kommen.
0 bedeutet, dass Sie keinerlei Schmerzen haben. Die stärksten Schmerzen, die Sie sich vorstellen können, haben den Zahlenwert 10.
Alle dazwischen liegenden Zahlen sollen Abstufungen darstellen.

*12. Wie schätzen Sie die durchschnittlichen Schmerzen während der **letzten 2 Wochen** ein?*

0 1 2 3 4 5 6 7 8 9 10 (Markierung bei 6)

*13. Welche Schmerzstärke wäre für Sie bei **erfolgreicher Behandlung** erträglich?*

0 1 2 3 4 5 6 7 8 9 10 (Markierung bei 2)

*14. Wie stark sind die Schmerzen **im Moment**?*

0 1 2 3 4 5 6 7 8 9 10 (Markierung bei 4)

Therapie

15. Wie wurden Ihre Schmerzen bereits behandelt?

☒ Krankengymnastik ☒ Massagen
☒ Akupunktur ☒ Kälte-/Wärmetherapie
☒ Einspritzung in das Schmerzgebiet
☒ Medikamente, ggf. welche Ibuprofen 400
☐ andere Behandlungen:

16. Nehmen Sie aktuell Medikamente zur Schmerzreduktion ein?

☐ ja ☒ nein

Wenn ja, geben Sie bitte Präparat und Dosierung an.

Wir fragen Sie nun nach den Schmerzen, die Sie haben, wenn Sie die Orthese tragen.
Bitte geben Sie die Stärke Ihrer Schmerzen an. Kreuzen Sie dafür auf den Linien die Zahlen an, die Ihrer Schmerzempfindung am nächsten kommen.
0 bedeutet, dass Sie keinerlei Schmerzen haben. Die stärksten Schmerzen, die Sie sich vorstellen können, haben den Zahlenwert 10. Alle dazwischen liegenden Zahlen sollen Abstufungen darstellen.

*17. Mit welchem Zahlenwert würden Sie die Schmerzen bei **leicht gestraffter Orthese** beschreiben?*

0 1 2 3 4 5 6 7 8 9 10 — x (bei 6)   im liegen

HK

Patienten-ID: 2011-10-17 -17P Fragebogen Teil 3

Wir fragen Sie nun noch einmal nach den Schmerzen, die Sie haben, wenn Sie die Orthese tragen.
Bitte geben Sie nun die Stärke Ihrer Schmerzen an. Kreuzen Sie dafür auf den Linien die Zahlen an, die Ihrer Schmerzempfindung am nächsten kommen.
0 bedeutet, dass Sie keinerlei Schmerzen haben. Die stärksten Schmerzen, die Sie sich vorstellen können, haben den Zahlenwert 10. Alle dazwischen liegenden Zahlen sollen Abstufungen darstellen.

Stehen Liegen

*18. Wie stark empfinden Sie die Schmerzen, wenn die **Orthese maximal gestrafft** ist?*

0 1 2 3 4 5 6 7 8 9 10

*19. Wie stark empfinden Sie die Schmerzen **nach Abnahme** der Orthese?*

0 1 2 3 4 5 6 7 8 9 10

20. Haben Sie das Gefühl, dass die Orthese selbst Ihnen Schmerzen verursacht? Wenn ja, wie stark sind diese?

0 1 2 3 4 5 6 7 8 9 10 im Liegen

Patienten-ID: 2011 - 10 - 17 - [illegible]

Fragebogen Teil 1

Liebe Patientin, lieber Patient,

Wir bitten Sie alle nachfolgenden Fragen wahrheitsgemäß zu beantworten.
Bitte kreuzen Sie für jede Frage, sofern nicht anders angegeben, ein Kästchen an!
Bearbeiten Sie zuerst die linke, dann die rechte Spalte.
Bei Fragen stehen wir jederzeit zur Verfügung.

Persönliche Angaben

1. Geburtsdatum: 24.10.65 Alter: 45
2. Geschlecht: ☐ männlich ☒ weiblich
3. Größe: 164
4. Gewicht: 69,3 5. BMI: 25,77
6. Beinigkeit: ☐ links ☒ rechts
7. Beckenumfang: 96,5 cm
   Orthesengröße: ☐ 1 ☒ 2 ☐ 3 ☐ 4

8. Haben sie das Gefühl, dass Sie sich unsicher beim Gehen fühlen, bzw. häufig stürzen?
   ☐ ja ☒ nein

Angaben zur Schmerzsymptomatik

9. Seit wann bestehen die Schmerzen?

☐ weniger als 1 Monat ☐ 1 Monat bis ½ Jahr
☐ ½ bis 1 Jahr ☐ 1 bis 2 Jahre
☐ 2 bis 5 Jahre ☒ mehr als 5 Jahre

10. Bitte zeichnen Sie ein, an welchen Körperstellen Ihre Schmerzen auftreten

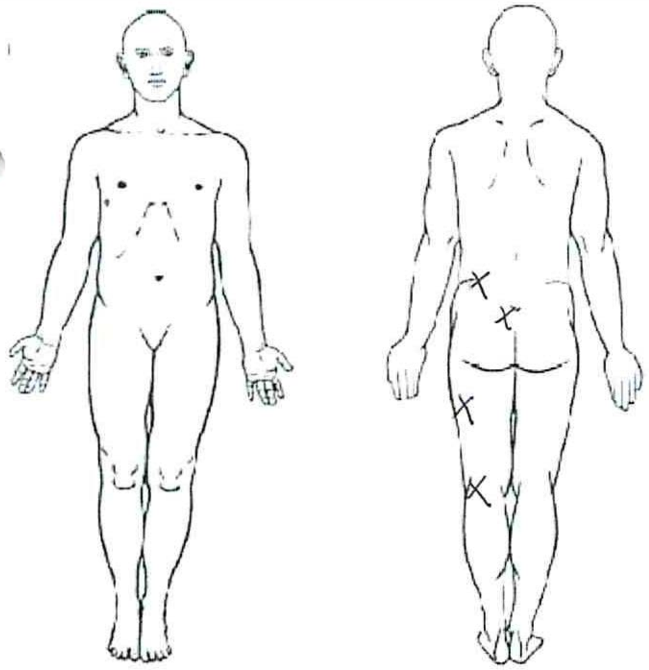

11. Bitte versuchen Sie die Schmerzqualität genauer zu beschreiben, das heißt, wie Sie die Schmerzen typischerweise empfinden. ***Mehrfachnennung ist möglich.***

☐ dumpf ☐ drückend ☒ pochend
☐ klopfend ☒ stechend ☐ ziehend
☐ heiß ☒ brennend ☒ unerträglich

Bitte geben Sie nun die Stärke Ihrer Schmerzen an. Kreuzen Sie dafür auf den Linien die Zahlen an, die Ihrer Schmerzempfindung am nächsten kommen.
0 bedeutet, dass Sie keinerlei Schmerzen haben. Die stärksten Schmerzen, die Sie sich vorstellen können, haben den Zahlenwert 10.
Alle dazwischen liegenden Zahlen sollen Abstufungen darstellen.

*12. Wie schätzen Sie die durchschnittlichen Schmerzen während der **letzten 2 Wochen** ein?*

0 1 2 3 4 5 6 7 8 9 ~~10~~ (angekreuzt: 10)

*13. Welche Schmerzstärke wäre für Sie bei **erfolgreicher Behandlung** erträglich?*

0 1 2 3 ~~4~~ 5 6 7 8 9 10 (angekreuzt: 4)

*14. Wie stark sind die Schmerzen **im Moment**?*

0 1 2 3 4 5 6 7 ~~8~~ 9 10 (angekreuzt: 8)

Therapie

15. Wie wurden Ihre Schmerzen bereits behandelt?

☒ Krankengymnastik ☒ Massagen
☐ Akupunktur ☐ Kälte-/Wärmetherapie
☒ Einspritzung in das Schmerzgebiet
☒ Medikamente, ggf. welche
☐ andere Behandlungen: ____________________

16. Nehmen Sie aktuell Medikamente zur Schmerzreduktion ein?
☒ ja ☐ nein

Wenn ja, geben Sie bitte Präparat und Dosierung an.
Palexia 1-0-1
Berlosin Tropf. 3+40 b.B.

[illegible]

Wir fragen Sie nun nach den Schmerzen, die Sie haben, wenn Sie die Orthese tragen.
Bitte geben Sie die Stärke Ihrer Schmerzen an. Kreuzen Sie dafür auf den Linien die Zahlen an, die Ihrer Schmerzempfindung am nächsten kommen.
0 bedeutet, dass Sie keinerlei Schmerzen haben. Die stärksten Schmerzen, die Sie sich vorstellen können, haben den Zahlenwert 10. Alle dazwischen liegenden Zahlen sollen Abstufungen darstellen.

*17. Mit welchem Zahlenwert würden Sie die Schmerzen bei **leicht gestraffter Orthese** beschreiben?*

0 1 2 3 4 5 6 7 8 9 10

(Kreuz bei 8)

HV

Wir fragen Sie nun noch einmal nach den Schmerzen, die Sie haben, wenn Sie die Orthese tragen.
Bitte geben Sie nun die Stärke Ihrer Schmerzen an. Kreuzen Sie dafür auf den Linien die Zahlen an, die Ihrer Schmerzempfindung am nächsten kommen.
0 bedeutet, dass Sie keinerlei Schmerzen haben. Die stärksten Schmerzen, die Sie sich vorstellen können, haben den Zahlenwert 10. Alle dazwischen liegenden Zahlen sollen Abstufungen darstellen.

18. Wie stark empfinden Sie die Schmerzen, wenn die ***Orthese maximal gestrafft*** *ist?*

0 1 2 3 4 5 6 7 8 9 10

19. Wie stark empfinden Sie die Schmerzen ***nach Abnahme*** *der Orthese?*

0 1 2 3 4 5 6 7 8 9 10

20. Haben Sie das Gefühl, dass die Orthese selbst Ihnen Schmerzen verursacht? Wenn ja, wie stark sind diese?

0 1 2 3 4 5 6 7 8 9 10 nein

Patienten-ID: 2011-11-11-21P

Fragebogen Teil 1

Liebe Patientin, lieber Patient,

Wir bitten Sie alle nachfolgenden Fragen wahrheitsgemäß zu beantworten.
Bitte kreuzen Sie für jede Frage, sofern nicht anders angegeben, ein Kästchen an!
Bearbeiten Sie zuerst die linke, dann die rechte Spalte.
Bei Fragen stehen wir jederzeit zur Verfügung.

Persönliche Angaben

1. Geburtsdatum: 28.10.62 Alter: 49
2. Geschlecht: [x] männlich [ ] weiblich
3. Größe: 180
4. Gewicht: 80 5. BMI: ______
6. Beinigkeit: [ ] links [x] rechts
7. Beckenumfang: 93 cm
   Orthesengröße: [ ] 1 [x] 2 [ ] 3 [ ] 4

8. Haben sie das Gefühl, dass Sie sich unsicher beim Gehen fühlen, bzw. häufig stürzen?
   [ ] ja [x] nein

Angaben zur Schmerzsymptomatik

9. Seit wann bestehen die Schmerzen?

[ ] weniger als 1 Monat
[ ] 1 Monat bis ½ Jahr
[ ] ½ bis 1 Jahr
[ ] 1 bis 2 Jahre
[ ] 2 bis 5 Jahre
[x] mehr als 5 Jahre

10. Bitte zeichnen Sie ein, an welchen Körperstellen Ihre Schmerzen auftreten

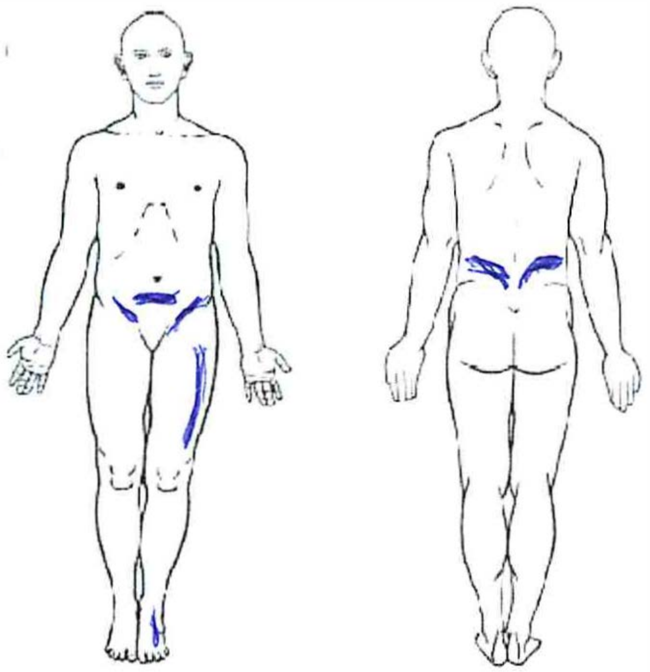

11. Bitte versuchen Sie die Schmerzqualität genauer zu beschreiben, das heißt, wie Sie die Schmerzen typischerweise empfinden. ***Mehrfachnennung ist möglich.***

[x] dumpf
[x] drückend
[ ] pochend
[ ] klopfend
[ ] stechend
[x] ziehend
[ ] heiß
[ ] brennend
[ ] unerträglich

Bitte geben Sie nun die Stärke Ihrer Schmerzen an. Kreuzen Sie dafür auf den Linien die Zahlen an, die Ihrer Schmerzempfindung am nächsten kommen.
0 bedeutet, dass Sie keinerlei Schmerzen haben. Die stärksten Schmerzen, die Sie sich vorstellen können, haben den Zahlenwert 10.
Alle dazwischen liegenden Zahlen sollen Abstufungen darstellen.

12. *Wie schätzen Sie die durchschnittlichen Schmerzen während der **letzten 2 Wochen** ein?*

0 1 2 3 4 5 6 7 8 9 10 (markiert: 4)

13. *Welche Schmerzstärke wäre für Sie bei **erfolgreicher Behandlung** erträglich?*

0 1 2 3 4 5 6 7 8 9 10 (markiert: zwischen 2 und 3)

14. *Wie stark sind die Schmerzen **im Moment**?*

0 1 2 3 4 5 6 7 8 9 10 (markiert: 3)

Therapie

15. Wie wurden Ihre Schmerzen bereits behandelt?

[x] Krankengymnastik
[x] Massagen
[x] Akupunktur
[x] Kälte-/Wärmetherapie
[x] Einspritzung in das Schmerzgebiet
[x] Medikamente, ggf. welche
[ ] andere Behandlungen: ______

16. Nehmen Sie aktuell Medikamente zur Schmerzreduktion ein?
   [x] ja [ ] nein

Wenn ja, geben Sie bitte Präparat und Dosierung an.

* KATADOLON S-LONG b. Bed
* Cymbalta 1x tgl.

[Unterschrift]

Wir fragen Sie nun nach den Schmerzen, die Sie haben, wenn Sie die Orthese tragen.
Bitte geben Sie die Stärke Ihrer Schmerzen an. Kreuzen Sie dafür auf den Linien die Zahlen an, die Ihrer Schmerzempfindung am nächsten kommen.
0 bedeutet, dass Sie keinerlei Schmerzen haben. Die stärksten Schmerzen, die Sie sich vorstellen können, haben den Zahlenwert 10. Alle dazwischen liegenden Zahlen sollen Abstufungen darstellen.

*17. Mit welchem Zahlenwert würden Sie die Schmerzen bei **leicht gestraffter Orthese** beschreiben?*

0 1 2 3 4 5 6 7 8 9 10

Ha

Wir fragen Sie nun noch einmal nach den Schmerzen, die Sie haben, wenn Sie die Orthese tragen.
Bitte geben Sie nun die Stärke Ihrer Schmerzen an. Kreuzen Sie dafür auf den Linien die Zahlen an, die Ihrer Schmerzempfindung am nächsten kommen.
0 bedeutet, dass Sie keinerlei Schmerzen haben. Die stärksten Schmerzen, die Sie sich vorstellen können, haben den Zahlenwert 10. Alle dazwischen liegenden Zahlen sollen Abstufungen darstellen.

*18. Wie stark empfinden Sie die Schmerzen, wenn die **Orthese maximal gestrafft** ist?*

0 1 2 3 4 5 6 7 8 9 10

*19. Wie stark empfinden Sie die Schmerzen **nach Abnahme** der Orthese?*

0 1 2 3 4 5 6 7 8 9 10

20. Haben Sie das Gefühl, dass die Orthese selbst Ihnen Schmerzen verursacht? Wenn ja, wie stark sind diese?

0 1 2 3 4 5 6 7 8 9 10

tar

Patienten-ID: 2012 - 02 - 03 - 25P Fragebogen Teil 1

Liebe Patientin, lieber Patient,

Wir bitten Sie alle nachfolgenden Fragen wahrheitsgemäß zu beantworten.
Bitte kreuzen Sie für jede Frage, sofern nicht anders angegeben, ein Kästchen an!
Bearbeiten Sie zuerst die linke, dann die rechte Spalte.
Bei Fragen stehen wir jederzeit zur Verfügung.

Persönliche Angaben

1. Geburtsdatum: 14.05.40 Alter: 71
2. Geschlecht: ☒ männlich ☐ weiblich
3. Größe: 175 cm
4. Gewicht: [illegible],5 5. BMI: 28,
6. Beinigkeit: ☐ links ☒ rechts
7. Beckenumfang: 97 cm
   Orthesengröße: ☐ 1 ☒ 2 ☐ 3 ☐ 4

8. Haben sie das Gefühl, dass Sie sich unsicher beim Gehen fühlen, bzw. häufig stürzen?
☐ ja ☒ nein

Angaben zur Schmerzsymptomatik

9. Seit wann bestehen die Schmerzen?

☐ weniger als 1 Monat ☐ 1 Monat bis ½ Jahr
☐ ½ bis 1 Jahr ☐ 1 bis 2 Jahre
☐ 2 bis 5 Jahre ☒ mehr als 5 Jahre

10. Bitte zeichnen Sie ein, an welchen Körperstellen Ihre Schmerzen auftreten

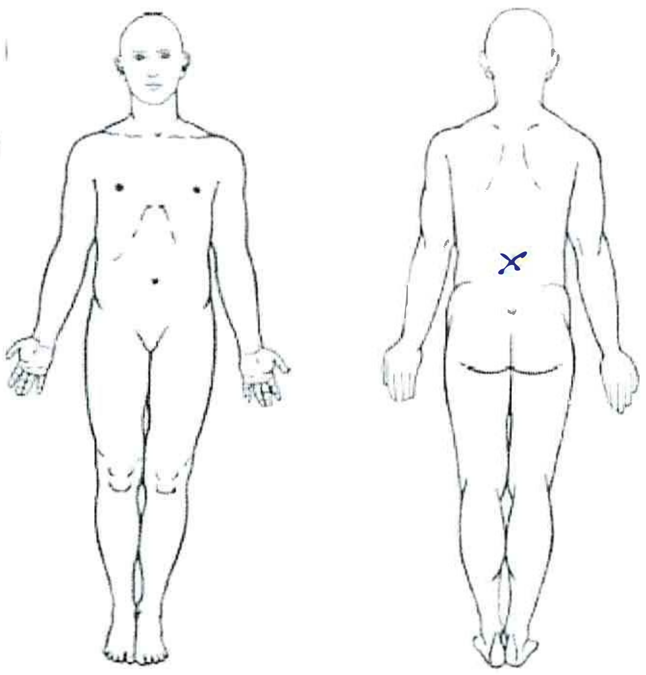

11. Bitte versuchen Sie die Schmerzqualität genauer zu beschreiben, das heißt, wie Sie die Schmerzen typischerweise empfinden. ***Mehrfachnennung ist möglich.***

☒ dumpf ☐ drückend ☐ pochend
☐ klopfend ☐ stechend ☒ ziehend
☐ heiß ☐ brennend ☐ unerträglich

Bitte geben Sie nun die Stärke Ihrer Schmerzen an. Kreuzen Sie dafür auf den Linien die Zahlen an, die Ihrer Schmerzempfindung am nächsten kommen.
0 bedeutet, dass Sie keinerlei Schmerzen haben. Die stärksten Schmerzen, die Sie sich vorstellen können, haben den Zahlenwert 10.
Alle dazwischen liegenden Zahlen sollen Abstufungen darstellen.

*12. Wie schätzen Sie die durchschnittlichen Schmerzen während der **letzten 2 Wochen** ein?*

0 1 ☒2 3 4 5 6 7 8 9 10

*13. Welche Schmerzstärke wäre für Sie bei **erfolgreicher Behandlung** erträglich?*

0 ☒1 2 3 4 5 6 7 8 9 10

*14. Wie stark sind die Schmerzen **im Moment**?*

0 1 ☒2 3 4 5 6 7 8 9 10

Therapie

15. Wie wurden Ihre Schmerzen bereits behandelt?

☒ Krankengymnastik ☒ Massagen
☐ Akupunktur ☐ Kälte-/Wärmetherapie
☐ Einspritzung in das Schmerzgebiet
☒ Medikamente, ggf. welche Diclo 75
☐ andere Behandlungen: ________________

16. Nehmen Sie aktuell Medikamente zur Schmerzreduktion ein?
☐ ja ☒ nein

Wenn ja, geben Sie bitte Präparat und Dosierung an.

Patienten-ID: 2012-02-05-25P

Fragebogen Teil 2

Wir fragen Sie nun nach den Schmerzen, die Sie haben, wenn Sie die Orthese tragen.
Bitte geben Sie die Stärke Ihrer Schmerzen an. Kreuzen Sie dafür auf den Linien die Zahlen an, die Ihrer Schmerzempfindung am nächsten kommen.
0 bedeutet, dass Sie keinerlei Schmerzen haben. Die stärksten Schmerzen, die Sie sich vorstellen können, haben den Zahlenwert 10. Alle dazwischen liegenden Zahlen sollen Abstufungen darstellen.

17. Mit welchem Zahlenwert würden Sie die Schmerzen bei ***leicht gestraffter Orthese*** *beschreiben?*

0 1 2 3 4 5 6 7 8 9 10

Patienten-ID: 2012 - 02 - 03 - 25P

Fragebogen Teil 3

Wir fragen Sie nun noch einmal nach den Schmerzen, die Sie haben, wenn Sie die Orthese tragen.
Bitte geben Sie nun die Stärke Ihrer Schmerzen an. Kreuzen Sie dafür auf den Linien die Zahlen an, die Ihrer Schmerzempfindung am nächsten kommen.
0 bedeutet, dass Sie keinerlei Schmerzen haben. Die stärksten Schmerzen, die Sie sich vorstellen können, haben den Zahlenwert 10. Alle dazwischen liegenden Zahlen sollen Abstufungen darstellen.

*18. Wie stark empfinden Sie die Schmerzen, wenn die **Orthese maximal gestrafft** ist?*

0 1 2 3 4 5 6 7 8 9 10

*19. Wie stark empfinden Sie die Schmerzen **nach Abnahme** der Orthese?*

0 1 2 3 4 5 6 7 8 9 10

20. Haben Sie das Gefühl, dass die Orthese selbst Ihnen Schmerzen verursacht? Wenn ja, wie stark sind diese?

0 1 2 3 4 5 6 7 8 9 10 ./.

Patienten-ID: 2012 - 03 - 12 - 27P

Fragebogen Teil 1

Liebe PatientIn, lieber Patient,

Wir bitten Sie alle nachfolgenden Fragen wahrheitsgemäß zu beantworten.
Bitte kreuzen Sie für jede Frage, sofern nicht anders angegeben, ein Kästchen an!
Bearbeiten Sie zuerst die linke, dann die rechte Spalte.
Bei Fragen stehen wir jederzeit zur Verfügung.

Persönliche Angaben

1. Geburtsdatum: 24 10 71 Alter: 40
2. Geschlecht: ☐ männlich ☒ weiblich
3. Größe: 178
4. Gewicht: 91,5 5. BMI: 28.8
6. Beinigkeit: ☐ links ☒ rechts
7. Beckenumfang: 111 cm

Orthesengröße: ☐ 1 ☐ 2 ☒ 3 ☐ 4

8. Haben sie das Gefühl, dass Sie sich unsicher beim Gehen fühlen, bzw. häufig stürzen?

☐ ja ☒ nein

Angaben zur Schmerzsymptomatik

9. Seit wann bestehen die Schmerzen?

☐ weniger als 1 Monat ☐ 1 Monat bis ½ Jahr
☐ ½ bis 1 Jahr ☐ 1 bis 2 Jahre
☒ 2 bis 5 Jahre ☐ mehr als 5 Jahre

10. Bitte zeichnen Sie ein, an welchen Körperstellen Ihre Schmerzen auftreten

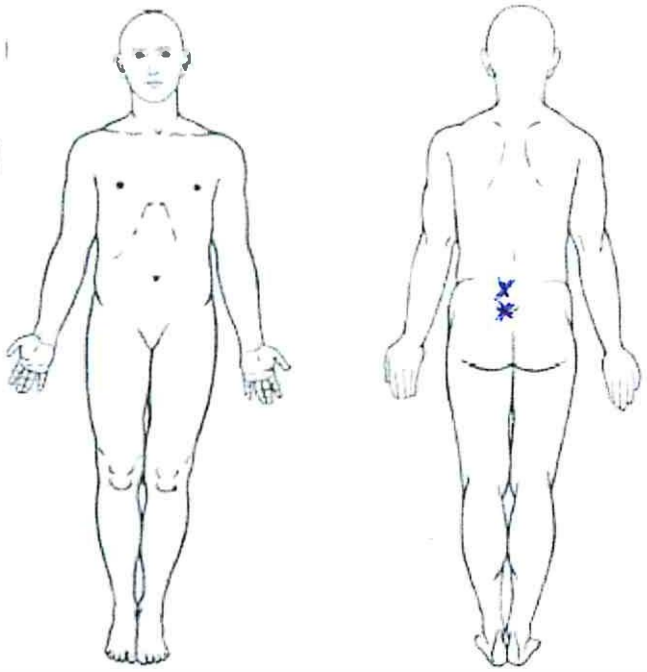

11. Bitte versuchen Sie die Schmerzqualität genauer zu beschreiben, das heißt, wie Sie die Schmerzen typischerweise empfinden. ***Mehrfachnennung ist möglich.***

☐ dumpf ☐ drückend ☐ pochend
☐ klopfend ☒ stechend ☒ ziehend
☐ heiß ☐ brennend ☒ unerträglich

Bitte geben Sie nun die Stärke Ihrer Schmerzen an. Kreuzen Sie dafür auf den Linien die Zahlen an, die ihrer Schmerzempfindung am nächsten kommen.
0 bedeutet, dass Sie keinerlei Schmerzen haben. Die stärksten Schmerzen, die Sie sich vorstellen können, haben den Zahlenwert 10.
Alle dazwischen liegenden Zahlen sollen Abstufungen darstellen.

*12. Wie schätzen Sie die durchschnittlichen Schmerzen während der **letzten 2 Wochen** ein?*

0 1 2 3 4 5 6 7 8 9 10 (angekreuzt: 3, 4, 5)

*13. Welche Schmerzstärke wäre für Sie bei **erfolgreicher Behandlung** erträglich?*

0 1 2 3 4 5 6 7 8 9 10 (angekreuzt: 2)

*14. Wie stark sind die Schmerzen **im Moment**?*

0 1 2 3 4 5 6 7 8 9 10 (angekreuzt: 3)

Therapie

15. Wie wurden Ihre Schmerzen bereits behandelt?

☒ Krankengymnastik ☒ Massagen
☐ Akupunktur ☐ Kälte-/Wärmetherapie
☒ Einspritzung in das Schmerzgebiet
☒ Medikamente, ggf. welche Diclac
☐ andere Behandlungen: ____

16. Nehmen Sie aktuell Medikamente zur Schmerzreduktion ein?

☒ ja ☐ nein

Wenn ja, geben Sie bitte Präparat und Dosierung an.

Diclac 2 x pro Woche

Wir fragen Sie nun nach den Schmerzen, die Sie haben, wenn Sie die Orthese tragen.
Bitte geben Sie die Stärke Ihrer Schmerzen an. Kreuzen Sie dafür auf den Linien die Zahlen an, die Ihrer Schmerzempfindung am nächsten kommen.
0 bedeutet, dass Sie keinerlei Schmerzen haben. Die stärksten Schmerzen, die Sie sich vorstellen können, haben den Zahlenwert 10. Alle dazwischen liegenden Zahlen sollen Abstufungen darstellen.

*17. Mit welchem Zahlenwert würden Sie die Schmerzen bei **leicht gestraffter Orthese** beschreiben?*

X (crossed out) at 0; X between 3 and 4

0 1 2 3 4 5 6 7 8 9 10

Wir fragen Sie nun noch einmal nach den Schmerzen, die Sie haben, wenn Sie die Orthese tragen.
Bitte geben Sie nun die Stärke Ihrer Schmerzen an. Kreuzen Sie dafür auf den Linien die Zahlen an, die Ihrer Schmerzempfindung am nächsten kommen.
0 bedeutet, dass Sie keinerlei Schmerzen haben. Die stärksten Schmerzen, die Sie sich vorstellen können, haben den Zahlenwert 10. Alle dazwischen liegenden Zahlen sollen Abstufungen darstellen.

*18. Wie stark empfinden Sie die Schmerzen, wenn die **Orthese maximal gestrafft** ist?*

0 1 2 3 4 ✗ 5 6 7 8 9 10

*19. Wie stark empfinden Sie die Schmerzen **nach Abnahme** der Orthese?*

0 1 2 3 ✗ 4 5 6 7 8 9 10

20. Haben Sie das Gefühl, dass die Orthese selbst Ihnen Schmerzen verursacht? Wenn ja, wie stark sind diese?

✗ 0 1 2 3 4 5 6 7 8 9 10

hr

Patienten-ID: 2012 - 06 - 04 - 37 P

Fragebogen Teil 1

Liebe Patientin, lieber Patient,

Wir bitten Sie alle nachfolgenden Fragen wahrheitsgemäß zu beantworten.
Bitte kreuzen Sie für jede Frage, sofern nicht anders angegeben, ein Kästchen an!
Bearbeiten Sie zuerst die linke, dann die rechte Spalte.
Bei Fragen stehen wir jederzeit zur Verfügung.

Persönliche Angaben

1. Geburtsdatum: 05.07.78 Alter: 33
2. Geschlecht: ☐ männlich ☒ weiblich
3. Größe: 153
4. Gewicht: 58 5. BMI: ______
6. Beinigkeit: ☐ links ☐ rechts
7. Beckenumfang: 84 cm
   Orthesengröße: ☒ 1 ☐ 2 ☐ 3 ☐ 4

8. Haben sie das Gefühl, dass Sie sich unsicher beim Gehen fühlen, bzw. häufig stürzen?
☐ ja ☒ nein

Angaben zur Schmerzsymptomatik

9. Seit wann bestehen die Schmerzen?

☐ weniger als 1 Monat ☐ 1 Monat bis ½ Jahr
☐ ½ bis 1 Jahr ☐ 1 bis 2 Jahre
☐ 2 bis 5 Jahre ☒ mehr als 5 Jahre

10. Bitte zeichnen Sie ein, an welchen Körperstellen Ihre Schmerzen auftreten

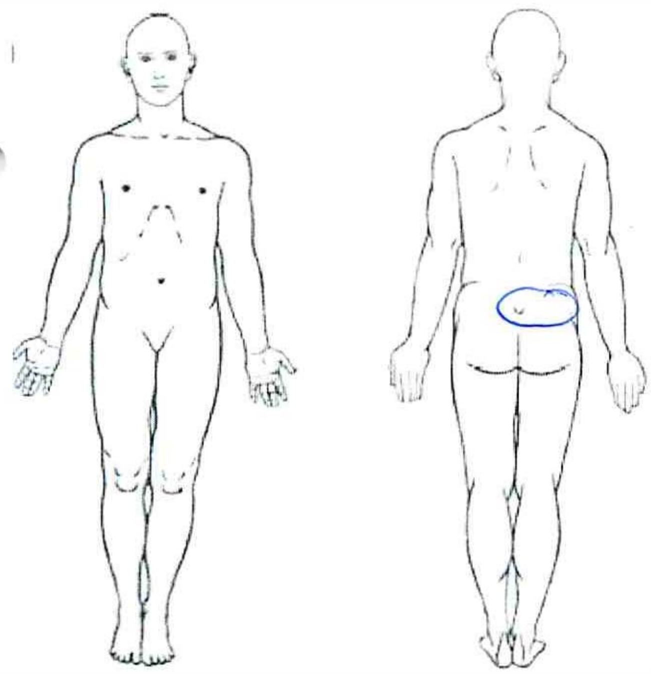

11. Bitte versuchen Sie die Schmerzqualität genauer zu beschreiben, das heißt, wie Sie die Schmerzen typischerweise empfinden. ***Mehrfachnennung ist möglich.***

☐ dumpf ☐ drückend ☐ pochend
☐ klopfend ☒ stechend ☒ ziehend
☐ heiß ☐ brennend ☐ unerträglich

Bitte geben Sie nun die Stärke Ihrer Schmerzen an. Kreuzen Sie dafür auf den Linien die Zahlen an, die Ihrer Schmerzempfindung am nächsten kommen.
0 bedeutet, dass Sie keinerlei Schmerzen haben. Die stärksten Schmerzen, die Sie sich vorstellen können, haben den Zahlenwert 10.
Alle dazwischen liegenden Zahlen sollen Abstufungen darstellen.

*12. Wie schätzen Sie die durchschnittlichen Schmerzen während der **letzten 2 Wochen** ein?*

0 1 2 3 (4) 5 6 7 8 9 10

*13. Welche Schmerzstärke wäre für Sie bei **erfolgreicher Behandlung** erträglich?*

0 1 (2) 3 4 5 6 7 8 9 10

*14. Wie stark sind die Schmerzen **im Moment**?*

0 1 2 3 (4) 5 6 7 8 9 10

Therapie

15. Wie wurden Ihre Schmerzen bereits behandelt?

☒ Krankengymnastik ☒ Massagen
☒ Akupunktur ☒ Kälte-/Wärmetherapie
☒ Einspritzung in das Schmerzgebiet
☒ Medikamente, ggf. welche [illegible]
☐ andere Behandlungen:

____

16. Nehmen Sie aktuell Medikamente zur Schmerzreduktion ein?
☐ ja ☒ nein

Wenn ja, geben Sie bitte Präparat und Dosierung an.

____
____
____
____
____

Wir fragen Sie nun nach den Schmerzen, die Sie haben, wenn Sie die Orthese tragen.
Bitte geben Sie die Stärke Ihrer Schmerzen an. Kreuzen Sie dafür auf den Linien die Zahlen an, die Ihrer Schmerzempfindung am nächsten kommen.
0 bedeutet, dass Sie keinerlei Schmerzen haben. Die stärksten Schmerzen, die Sie sich vorstellen können, haben den Zahlenwert 10. Alle dazwischen liegenden Zahlen sollen Abstufungen darstellen.

17. Mit welchem Zahlenwert würden Sie die Schmerzen bei ***leicht gestraffter Orthese*** *beschreiben?*

0 1 2 (3) 4 5 6 7 8 9 10

Patienten-ID: 2012 - 06 - 04 -31P Fragebogen Teil 3

Wir fragen Sie nun noch einmal nach den Schmerzen, die Sie haben, wenn Sie die Orthese tragen.
Bitte geben Sie nun die Stärke Ihrer Schmerzen an. Kreuzen Sie dafür auf den Linien die Zahlen an, die Ihrer Schmerzempfindung am nächsten kommen.
0 bedeutet, dass Sie keinerlei Schmerzen haben. Die stärksten Schmerzen, die Sie sich vorstellen können, haben den Zahlenwert 10. Alle dazwischen liegenden Zahlen sollen Abstufungen darstellen.

*18. Wie stark empfinden Sie die Schmerzen, wenn die **Orthese maximal gestrafft** ist?*

0 1 2 (3) 4 5 6 7 8 9 10

*19. Wie stark empfinden Sie die Schmerzen **nach Abnahme** der Orthese?*

0 1 2 3 (4) 5 6 7 8 9 10

20. Haben Sie das Gefühl, dass die Orthese selbst Ihnen Schmerzen verursacht? Wenn ja, wie stark sind diese? nein

0 1 2 3 4 5 6 7 8 9 10

Patienten-ID: 2012 - 06 - 11 - 32P

Fragebogen Teil 1

Liebe Patientin, lieber Patient,

Wir bitten Sie alle nachfolgenden Fragen wahrheitsgemäß zu beantworten.
Bitte kreuzen Sie für jede Frage, sofern nicht anders angegeben, ein Kästchen an!
Bearbeiten Sie zuerst die linke, dann die rechte Spalte.
Bei Fragen stehen wir jederzeit zur Verfügung.

Persönliche Angaben

1. Geburtsdatum: 11.06.73 Alter: 39
2. Geschlecht: ☐ männlich ☒ weiblich
3. Größe: 178 cm
4. Gewicht: 64 kg 5. BMI: 20.3
6. Beinigkeit: ☒ links ☐ rechts
7. Beckenumfang: 80 cm
   Orthesengröße: ☐ 1 ☒ 2 ☐ 3 ☐ 4

8. Haben sie das Gefühl, dass Sie sich unsicher beim Gehen fühlen, bzw. häufig stürzen?
☐ ja ☒ nein

Angaben zur Schmerzsymptomatik

9. Seit wann bestehen die Schmerzen?

☐ weniger als 1 Monat ☐ 1 Monat bis ½ Jahr
☐ ½ bis 1 Jahr ☐ 1 bis 2 Jahre
☒ 2 bis 5 Jahre ☐ mehr als 5 Jahre

10. Bitte zeichnen Sie ein, an welchen Körperstellen Ihre Schmerzen auftreten

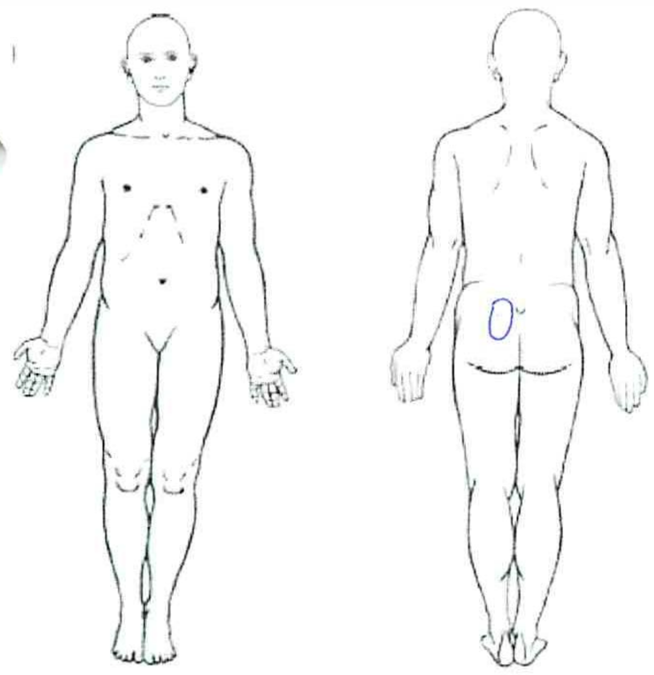

11. Bitte versuchen Sie die Schmerzqualität genauer zu beschreiben, das heißt, wie Sie die Schmerzen typischerweise empfinden. ***Mehrfachnennung ist möglich.***

☐ dumpf ☐ drückend ☐ pochend
☐ klopfend ☐ stechend ☐ ziehend
☒ heiß ☒ brennend ☐ unerträglich

Bitte geben Sie nun die Stärke Ihrer Schmerzen an. Kreuzen Sie dafür auf den Linien die Zahlen an, die Ihrer Schmerzempfindung am nächsten kommen.
0 bedeutet, dass Sie keinerlei Schmerzen haben. Die stärksten Schmerzen, die Sie sich vorstellen können, haben den Zahlenwert 10.
Alle dazwischen liegenden Zahlen sollen Abstufungen darstellen.

*12. Wie schätzen Sie die durchschnittlichen Schmerzen während der **letzten 2 Wochen** ein?*

0 1 2 3 4 5 6 7 8 9 10 (Markierung zwischen 3 und 4)

*13. Welche Schmerzstärke wäre für Sie bei **erfolgreicher Behandlung** erträglich?*

0 1 2 3 4 5 6 7 8 9 10 (Markierung bei 2)

*14. Wie stark sind die Schmerzen **im Moment**?*

0 1 2 3 4 5 6 7 8 9 10 (Markierung bei 3)

Therapie

15. Wie wurden Ihre Schmerzen bereits behandelt?

☒ Krankengymnastik ☒ Massagen
☐ Akupunktur ☐ Kälte-/Wärmetherapie
☒ Einspritzung in das Schmerzgebiet
☐ Medikamente, ggf. welche
☐ andere Behandlungen:

16. Nehmen Sie aktuell Medikamente zur Schmerzreduktion ein?
☐ ja ☒ nein

Wenn ja, geben Sie bitte Präparat und Dosierung an.

AS

Wir fragen Sie nun nach den Schmerzen, die Sie haben, wenn Sie die Orthese tragen.
Bitte geben Sie die Stärke Ihrer Schmerzen an. Kreuzen Sie dafür auf den Linien die Zahlen an, die Ihrer Schmerzempfindung am nächsten kommen.
0 bedeutet, dass Sie keinerlei Schmerzen haben. Die stärksten Schmerzen, die Sie sich vorstellen können, haben den Zahlenwert 10. Alle dazwischen liegenden Zahlen sollen Abstufungen darstellen.

17. Mit welchem Zahlenwert würden Sie die Schmerzen bei ***leicht gestraffter Orthese*** *beschreiben?*

0 1 2 3 4 5 6 7 8 9 10

Patienten-ID: 2012 - 06 - 11 - 32P

Fragebogen Teil 3

Wir fragen Sie nun noch einmal nach den Schmerzen, die Sie haben, wenn Sie die Orthese tragen.
Bitte geben Sie nun die Stärke Ihrer Schmerzen an. Kreuzen Sie dafür auf den Linien die Zahlen an, die Ihrer Schmerzempfindung am nächsten kommen.
0 bedeutet, dass Sie keinerlei Schmerzen haben. Die stärksten Schmerzen, die Sie sich vorstellen können, haben den Zahlenwert 10. Alle dazwischen liegenden Zahlen sollen Abstufungen darstellen.

*18. Wie stark empfinden Sie die Schmerzen, wenn die **Orthese maximal gestrafft** ist?*

0 1 2 3 ✓ 4 5 6 7 8 9 10

*19. Wie stark empfinden Sie die Schmerzen **nach Abnahme** der Orthese?*

0 1 2 ✓ 3 4 5 6 7 8 9 10

20. Haben Sie das Gefühl, dass die Orthese selbst Ihnen Schmerzen verursacht? Wenn ja, wie stark sind diese?

X 0 1 2 3 4 5 6 7 8 9 10

Patienten-ID: 2012 - 06 - 15 - 33P

Fragebogen Teil 1

Liebe Patientin, lieber Patient,

Wir bitten Sie alle nachfolgenden Fragen wahrheitsgemäß zu beantworten.
Bitte kreuzen Sie für jede Frage, sofern nicht anders angegeben, ein Kästchen an!
Bearbeiten Sie zuerst die linke, dann die rechte Spalte.
Bei Fragen stehen wir jederzeit zur Verfügung.

Persönliche Angaben

1. Geburtsdatum: 03.03.66 Alter: 46
2. Geschlecht: ☒ männlich ☐ weiblich
3. Größe: 1,78
4. Gewicht: 85,3 kg 5. BMI: 24.4
6. Beinigkeit: ☐ links ☒ rechts
7. Beckenumfang: 95,5 cm

Orthesengröße: ☐ 1 ☒ 2 ☐ 3 ☐ 4

8. Haben sie das Gefühl, dass Sie sich unsicher beim Gehen fühlen, bzw. häufig stürzen?

☐ ja ☒ nein

Angaben zur Schmerzsymptomatik

9. Seit wann bestehen die Schmerzen?

☐ weniger als 1 Monat ☐ 1 Monat bis ½ Jahr
☒ ½ bis 1 Jahr ☐ 1 bis 2 Jahre
☐ 2 bis 5 Jahre ☐ mehr als 5 Jahre

10. Bitte zeichnen Sie ein, an welchen Körperstellen Ihre Schmerzen auftreten

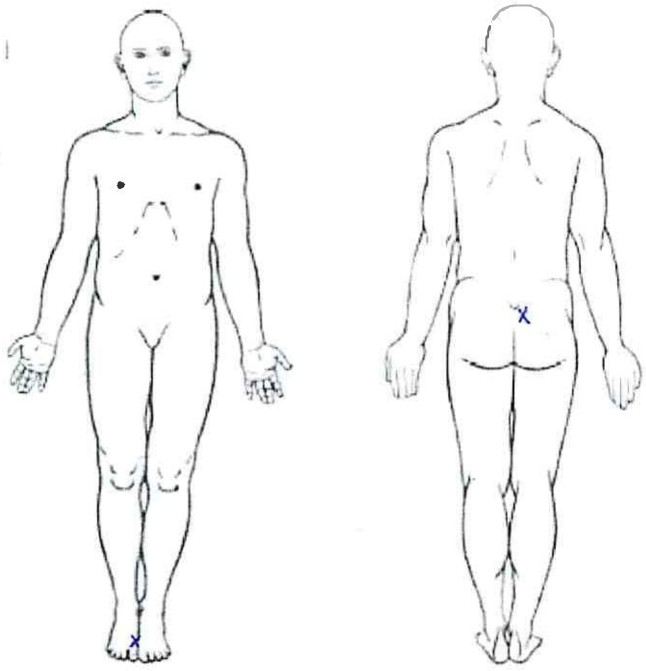

11. Bitte versuchen Sie die Schmerzqualität genauer zu beschreiben, das heißt, wie Sie die Schmerzen typischerweise empfinden. ***Mehrfachnennung ist möglich.***

☐ dumpf ☐ drückend ☐ pochend
☐ klopfend ☐ stechend ☐ ziehend
☒ heiß ☒ brennend ☐ unerträglich

Bitte geben Sie nun die Stärke Ihrer Schmerzen an. Kreuzen Sie dafür auf den Linien die Zahlen an, die Ihrer Schmerzempfindung am nächsten kommen.
0 bedeutet, dass Sie keinerlei Schmerzen haben. Die stärksten Schmerzen, die Sie sich vorstellen können, haben den Zahlenwert 10.
Alle dazwischen liegenden Zahlen sollen Abstufungen darstellen.

*12. Wie schätzen Sie die durchschnittlichen Schmerzen während der **letzten 2 Wochen** ein?*

0 1 2 3 4 5 ☒6 7 8 9 10

*13. Welche Schmerzstärke wäre für Sie bei **erfolgreicher Behandlung** erträglich?*

0 1 ☒2 3 4 5 6 7 8 9 10

*14. Wie stark sind die Schmerzen **im Moment**?*

0 1 2 3 ☒4 5 6 7 8 9 10

Therapie

15. Wie wurden Ihre Schmerzen bereits behandelt?

☒ Krankengymnastik ☐ Massagen
☐ Akupunktur ☐ Kälte-/Wärmetherapie
☒ Einspritzung in das Schmerzgebiet
☒ Medikamente, ggf. welche
☐ andere Behandlungen:

16. Nehmen Sie aktuell Medikamente zur Schmerzreduktion ein?

☒ ja ☐ nein

Wenn ja, geben Sie bitte Präparat und Dosierung an.

hr

Wir fragen Sie nun nach den Schmerzen, die Sie haben, wenn Sie die Orthese tragen.
Bitte geben Sie die Stärke Ihrer Schmerzen an. Kreuzen Sie dafür auf den Linien die Zahlen an, die Ihrer Schmerzempfindung am nächsten kommen.
0 bedeutet, dass Sie keinerlei Schmerzen haben. Die stärksten Schmerzen, die Sie sich vorstellen können, haben den Zahlenwert 10. Alle dazwischen liegenden Zahlen sollen Abstufungen darstellen.

17. Mit welchem Zahlenwert würden Sie die Schmerzen bei ***leicht gestraffter Orthese*** *beschreiben?*

X

0 1 2 3 4 5 6 7 8 9 10

Patienten-ID: 2012 - 06 - 15 - 33P Fragebogen Teil 3

Wir fragen Sie nun noch einmal nach den Schmerzen, die Sie haben, wenn Sie die Orthese tragen.
Bitte geben Sie nun die Stärke Ihrer Schmerzen an. Kreuzen Sie dafür auf den Linien die Zahlen an, die Ihrer Schmerzempfindung am nächsten kommen.
0 bedeutet, dass Sie keinerlei Schmerzen haben. Die stärksten Schmerzen, die Sie sich vorstellen können, haben den Zahlenwert 10. Alle dazwischen liegenden Zahlen sollen Abstufungen darstellen.

*18. Wie stark empfinden Sie die Schmerzen, wenn die **Orthese maximal gestrafft** ist?*

0 1 2 3 (X) 4 5 6 7 8 9 10

*19. Wie stark empfinden Sie die Schmerzen **nach Abnahme** der Orthese?*

0 1 2 (X) 3 4 5 6 7 8 9 10

20. Haben Sie das Gefühl, dass die Orthese selbst Ihnen Schmerzen verursacht? Wenn ja, wie stark sind diese?

0 1 2 3 4 5 6 (X) 7 8 9 10

Patienten-ID: 2012 - 06 - 18 - 39P

Fragebogen Teil 1

Liebe Patientin, lieber Patient,

Wir bitten Sie alle nachfolgenden Fragen wahrheitsgemäß zu beantworten.
Bitte kreuzen Sie für jede Frage, sofern nicht anders angegeben, ein Kästchen an!
Bearbeiten Sie zuerst die linke, dann die rechte Spalte.
Bei Fragen stehen wir jederzeit zur Verfügung.

Persönliche Angaben

1. Geburtsdatum: 23.12.56 Alter: 55
2. Geschlecht: ☒ männlich ☐ weiblich
3. Größe: 168 cm
4. Gewicht: 88 kg 5. BMI: 31,3
6. Beinigkeit: ☒ links ☐ rechts
7. Beckenumfang: 102 cm
   Orthesengröße: ☐ 1 ☐ 2 ☒ 3 ☐ 4

8. Haben sie das Gefühl, dass Sie sich unsicher beim Gehen fühlen, bzw. häufig stürzen?
   ☐ ja ☒ nein

Angaben zur Schmerzsymptomatik

9. Seit wann bestehen die Schmerzen?

☐ weniger als 1 Monat ☐ 1 Monat bis ½ Jahr
☐ ½ bis 1 Jahr ☒ 1 bis 2 Jahre
☐ 2 bis 5 Jahre ☐ mehr als 5 Jahre

10. Bitte zeichnen Sie ein, an welchen Körperstellen Ihre Schmerzen auftreten

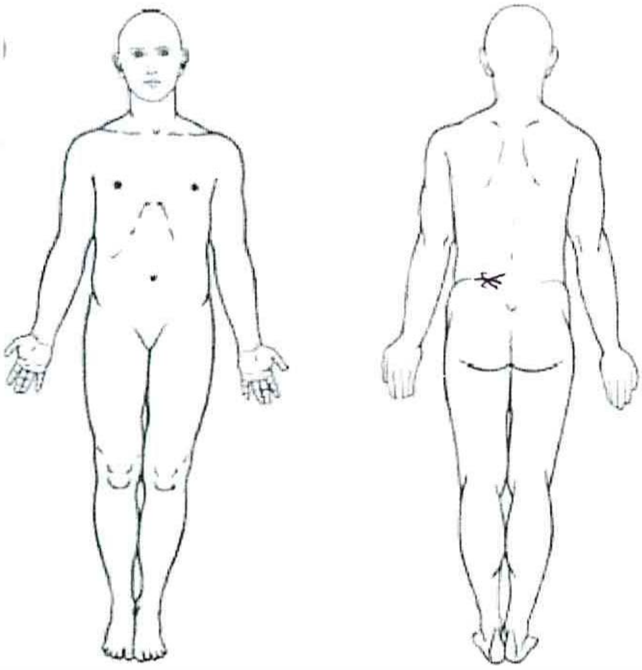

11. Bitte versuchen Sie die Schmerzqualität genauer zu beschreiben, das heißt, wie Sie die Schmerzen typischerweise empfinden. ***Mehrfachnennung ist möglich.***

☐ dumpf ☒ drückend ☒ pochend
☒ klopfend ☒ stechend ☐ ziehend
☐ heiß ☐ brennend ☐ unerträglich

Bitte geben Sie nun die Stärke Ihrer Schmerzen an. Kreuzen Sie dafür auf den Linien die Zahlen an, die Ihrer Schmerzempfindung am nächsten kommen.
0 bedeutet, dass Sie keinerlei Schmerzen haben. Die stärksten Schmerzen, die Sie sich vorstellen können, haben den Zahlenwert 10.
Alle dazwischen liegenden Zahlen sollen Abstufungen darstellen.

*12. Wie schätzen Sie die durchschnittlichen Schmerzen während der **letzten 2 Wochen** ein?*

0 1 2 3 [x] 4 5 6 7 8 9 10

*13. Welche Schmerzstärke wäre für Sie bei **erfolgreicher Behandlung** erträglich?*

0 1 2 3 4 5 6 7 8 9 10

*14. Wie stark sind die Schmerzen **im Moment**?*

0 1 2 3 4 5 [x] 6 7 8 9 10

Therapie

15. Wie wurden Ihre Schmerzen bereits behandelt?

☐ Krankengymnastik ☒ Massagen
☐ Akupunktur ☐ Kälte-/Wärmetherapie
☒ Einspritzung in das Schmerzgebiet
☐ Medikamente, ggf. welche
☐ andere Behandlungen:

16. Nehmen Sie aktuell Medikamente zur Schmerzreduktion ein?
   ☐ ja ☒ nein

Wenn ja, geben Sie bitte Präparat und Dosierung an.

WS

Wir fragen Sie nun nach den Schmerzen, die Sie haben, wenn Sie die Orthese tragen.
Bitte geben Sie die Stärke Ihrer Schmerzen an. Kreuzen Sie dafür auf den Linien die Zahlen an, die Ihrer Schmerzempfindung am nächsten kommen.
0 bedeutet, dass Sie keinerlei Schmerzen haben. Die stärksten Schmerzen, die Sie sich vorstellen können, haben den Zahlenwert 10. Alle dazwischen liegenden Zahlen sollen Abstufungen darstellen.

*17. Mit welchem Zahlenwert würden Sie die Schmerzen bei **leicht gestraffter Orthese** beschreiben?*

X (angekreuzt: 0)

0 1 2 3 4 5 6 7 8 9 10

Ww

Patienten-ID: 2012 - 06 - 18 - 34P Fragebogen Teil 3

Wir fragen Sie nun noch einmal nach den Schmerzen, die Sie haben, wenn Sie die Orthese tragen.
Bitte geben Sie nun die Stärke Ihrer Schmerzen an. Kreuzen Sie dafür auf den Linien die Zahlen an, die Ihrer Schmerzempfindung am nächsten kommen.
0 bedeutet, dass Sie keinerlei Schmerzen haben. Die stärksten Schmerzen, die Sie sich vorstellen können, haben den Zahlenwert 10. Alle dazwischen liegenden Zahlen sollen Abstufungen darstellen.

*18. Wie stark empfinden Sie die Schmerzen, wenn die **Orthese maximal gestrafft** ist?*

0 1 2 3 4 5 6 7 8 9 10 (X bei 4)

*19. Wie stark empfinden Sie die Schmerzen **nach Abnahme** der Orthese?*

0 1 2 3 4 5 6 7 8 9 10 (X bei 0)

20. Haben Sie das Gefühl, dass die Orthese selbst Ihnen Schmerzen verursacht? Wenn ja, wie stark sind diese?

0 1 2 3 4 5 6 7 8 9 10 (X bei 5)

Patienten-ID: 2012-07-24-35P

Fragebogen Teil 1

Liebe Patientin, lieber Patient,

Wir bitten Sie alle nachfolgenden Fragen wahrheitsgemäß zu beantworten.
Bitte kreuzen Sie für jede Frage, sofern nicht anders angegeben, ein Kästchen an!
Bearbeiten Sie zuerst die linke, dann die rechte Spalte.
Bei Fragen stehen wir jederzeit zur Verfügung.

Persönliche Angaben

1. Geburtsdatum: 13.4.61 Alter: 51
2. Geschlecht: ☐ männlich ☒ weiblich
3. Größe: 167
4. Gewicht: 61 5. BMI: ______
6. Beinigkeit: ☒ links ☐ rechts
7. Beckenumfang: 90,5 cm

Orthesengröße: ☐ 1 ☒ 2 ☐ 3 ☐ 4

8. Haben sie das Gefühl, dass Sie sich unsicher beim Gehen fühlen, bzw. häufig stürzen?

☐ ja ☒ nein

Angaben zur Schmerzsymptomatik

9. Seit wann bestehen die Schmerzen?

☐ weniger als 1 Monat ☐ 1 Monat bis ½ Jahr
☐ ½ bis 1 Jahr ☐ 1 bis 2 Jahre
☐ 2 bis 5 Jahre ☒ mehr als 5 Jahre

10. Bitte zeichnen Sie ein, an welchen Körperstellen Ihre Schmerzen auftreten

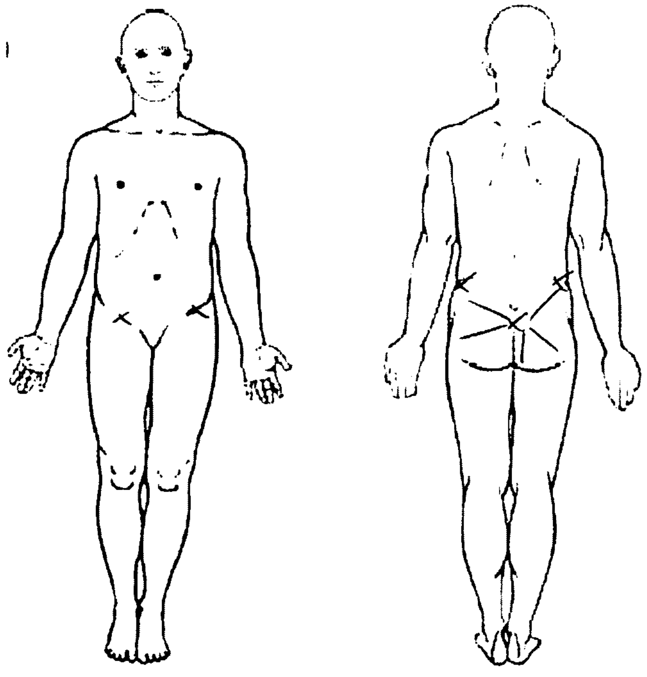

11. Bitte versuchen Sie die Schmerzqualität genauer zu beschreiben, das heißt, wie Sie die Schmerzen typischerweise empfinden. ***Mehrfachnennung ist möglich.***

☒ dumpf ☒ drückend ☐ pochend
☐ klopfend ☒ stechend ☒ ziehend
☐ heiß ☐ brennend ☐ unerträglich

Bitte geben Sie nun die Stärke Ihrer Schmerzen an. Kreuzen Sie dafür auf den Linien die Zahlen an, die Ihrer Schmerzempfindung am nächsten kommen.
0 bedeutet, dass Sie keinerlei Schmerzen haben. Die stärksten Schmerzen, die Sie sich vorstellen können, haben den Zahlenwert 10.
Alle dazwischen liegenden Zahlen sollen Abstufungen darstellen.

*12. Wie schätzen Sie die durchschnittlichen Schmerzen während der **letzten 2 Wochen** ein?*

0 1 2 3 4 5 6 X 7 8 9 10

*13. Welche Schmerzstärke wäre für Sie bei **erfolgreicher Behandlung** erträglich?*

0 1 X 2 3 4 5 6 7 8 9 10

*14. Wie stark sind die Schmerzen **im Moment**?*

0 1 2 3 4 5 X 6 7 8 9 10

Therapie

15. Wie wurden Ihre Schmerzen bereits behandelt?

☒ Krankengymnastik ☐ Massagen
☐ Akupunktur ☒ Kälte-/Wärmetherapie
☒ Einspritzung in das Schmerzgebiet
☒ Medikamente, ggf. welche
☐ andere Behandlungen:

______

16. Nehmen Sie aktuell Medikamente zur Schmerzreduktion ein?

☐ ja ☒ nein

Wenn ja, geben Sie bitte Präparat und Dosierung an.

______
______
______
______
______

Wir fragen Sie nun nach den Schmerzen, die Sie haben, wenn Sie die Orthese tragen.
Bitte geben Sie die Stärke Ihrer Schmerzen an. Kreuzen Sie dafür auf den Linien die Zahlen an, die Ihrer Schmerzempfindung am nächsten kommen.
0 bedeutet, dass Sie keinerlei Schmerzen haben. Die stärksten Schmerzen, die Sie sich vorstellen können, haben den Zahlenwert 10. Alle dazwischen liegenden Zahlen sollen Abstufungen darstellen.

17. Mit welchem Zahlenwert würden Sie die Schmerzen bei ***leicht gestraffter Orthese*** *beschreiben?*

0 1 2 3 4 X(5) 6 7 8 9 10

4r

Patienten-ID: 2012 - 07 - 24 - 35 P Fragebogen Teil 3

Wir fragen Sie nun noch einmal nach den Schmerzen, die Sie haben, wenn Sie die Orthese tragen.
Bitte geben Sie nun die Stärke Ihrer Schmerzen an. Kreuzen Sie dafür auf den Linien die Zahlen an, die Ihrer Schmerzempfindung am nächsten kommen.
0 bedeutet, dass Sie keinerlei Schmerzen haben. Die stärksten Schmerzen, die Sie sich vorstellen können, haben den Zahlenwert 10. Alle dazwischen liegenden Zahlen sollen Abstufungen darstellen.

*18. Wie stark empfinden Sie die Schmerzen, wenn die **Orthese maximal gestrafft** ist?*

0 1 2 3 4 5 6 7 8 9 10

*19. Wie stark empfinden Sie die Schmerzen **nach Abnahme** der Orthese?*

0 1 2 3 4 5 6 7 8 9 10

20. Haben Sie das Gefühl, dass die Orthese selbst Ihnen Schmerzen verursacht? Wenn ja, wie stark sind diese?

0 1 2 3 4 5 6 7 8 9 10

Patienten-ID: 2012 - 07 - 30 - 35P

Fragebogen Teil 1

Liebe Patientin, lieber Patient,

Wir bitten Sie alle nachfolgenden Fragen wahrheitsgemäß zu beantworten.
Bitte kreuzen Sie für jede Frage, sofern nicht anders angegeben, ein Kästchen an!
Bearbeiten Sie zuerst die linke, dann die rechte Spalte.
Bei Fragen stehen wir jederzeit zur Verfügung.

Persönliche Angaben

1. Geburtsdatum: 07.07.80 Alter: 32
2. Geschlecht: [x] männlich [ ] weiblich
3. Größe: 174 cm
4. Gewicht: 66,9 kg 5. BMI: ______
6. Beinigkeit: [ ] links [x] rechts
7. Beckenumfang: 85 cm

   Orthesengröße: [x] 1 [ ] 2 [ ] 3 [ ] 4

8. Haben sie das Gefühl, dass Sie sich unsicher beim Gehen fühlen, bzw. häufig stürzen?

   [ ] ja [x] nein

Angaben zur Schmerzsymptomatik

9. Seit wann bestehen die Schmerzen?

   [ ] weniger als 1 Monat [ ] 1 Monat bis ½ Jahr
   [ ] ½ bis 1 Jahr [x] 1 bis 2 Jahre
   [ ] 2 bis 5 Jahre [ ] mehr als 5 Jahre

10. Bitte zeichnen Sie ein, an welchen Körperstellen Ihre Schmerzen auftreten

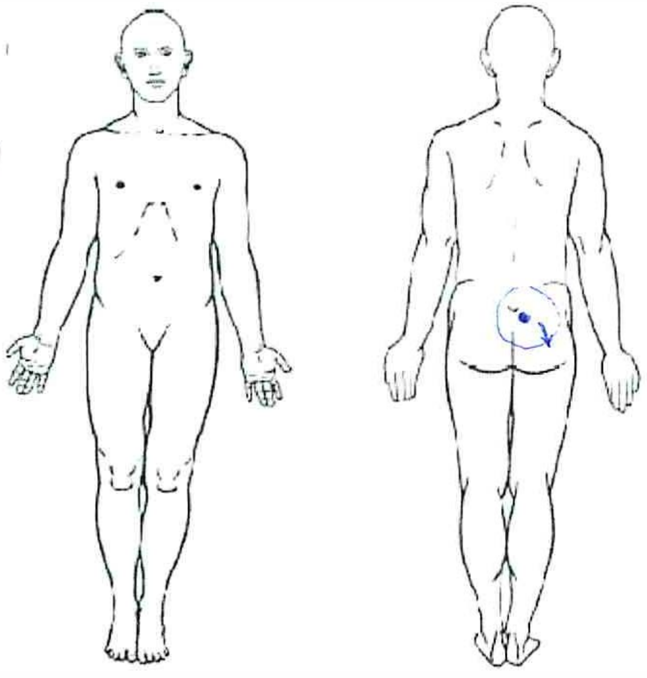

11. Bitte versuchen Sie die Schmerzqualität genauer zu beschreiben, das heißt, wie Sie die Schmerzen typischerweise empfinden. ***Mehrfachnennung ist möglich.***

   [x] dumpf [x] drückend [ ] pochend
   [ ] klopfend [ ] stechend [ ] ziehend
   [x] heiß [ ] brennend [ ] unerträglich

Bitte geben Sie nun die Stärke Ihrer Schmerzen an. Kreuzen Sie dafür auf den Linien die Zahlen an, die Ihrer Schmerzempfindung am nächsten kommen.
0 bedeutet, dass Sie keinerlei Schmerzen haben. Die stärksten Schmerzen, die Sie sich vorstellen können, haben den Zahlenwert 10.
Alle dazwischen liegenden Zahlen sollen Abstufungen darstellen.

12. *Wie schätzen Sie die durchschnittlichen Schmerzen während der **letzten 2 Wochen** ein?*

   0 1 2 (3) 4 5 6 7 8 9 10

13. *Welche Schmerzstärke wäre für Sie bei **erfolgreicher Behandlung** erträglich?*

   0 1 | 2 3 4 5 6 7 8 9 10

14. *Wie stark sind die Schmerzen **im Moment**?*

   0 1 (2) 3 4 5 6 7 8 9 10

Therapie

15. Wie wurden ihre Schmerzen bereits behandelt?

   [x] Krankengymnastik [x] Massagen
   [ ] Akupunktur [ ] Kälte-/Wärmetherapie
   [ ] Einspritzung in das Schmerzgebiet
   [ ] Medikamente, ggf. welche
   [ ] andere Behandlungen:
   ______

16. Nehmen Sie aktuell Medikamente zur Schmerzreduktion ein?

   [ ] ja [x] nein

Wenn ja, geben Sie bitte Präparat und Dosierung an.

______
______
______
______
______

Wir fragen Sie nun nach den Schmerzen, die Sie haben, wenn Sie die Orthese tragen.
Bitte geben Sie die Stärke Ihrer Schmerzen an. Kreuzen Sie dafür auf den Linien die Zahlen an, die Ihrer Schmerzempfindung am nächsten kommen.
0 bedeutet, dass Sie keinerlei Schmerzen haben. Die stärksten Schmerzen, die Sie sich vorstellen können, haben den Zahlenwert 10. Alle dazwischen liegenden Zahlen sollen Abstufungen darstellen.

17. Mit welchem Zahlenwert würden Sie die Schmerzen bei ***leicht gestraffter Orthese*** *beschreiben?*

(0) 1 2 3 4 5 6 7 8 9 10

Ur

Wir fragen Sie nun noch einmal nach den Schmerzen, die Sie haben, wenn Sie die Orthese tragen:
Bitte geben Sie nun die Stärke Ihrer Schmerzen an. Kreuzen Sie dafür auf den Linien die Zahlen an, die Ihrer Schmerzempfindung am nächsten kommen.
0 bedeutet, dass Sie keinerlei Schmerzen haben. Die stärksten Schmerzen, die Sie sich vorstellen können, haben den Zahlenwert 10. Alle dazwischen liegenden Zahlen sollen Abstufungen darstellen.

*18. Wie stark empfinden Sie die Schmerzen, wenn die **Orthese maximal gestrafft** ist?*

(0) 1 2 3 4 5 6 7 8 9 10

*19. Wie stark empfinden Sie die Schmerzen **nach Abnahme** der Orthese?*

0 (1) 2 3 4 5 6 7 8 9 10

20. Haben Sie das Gefühl, dass die Orthese selbst Ihnen Schmerzen verursacht? Wenn ja, wie stark sind diese?

(0) 1 2 3 4 5 6 7 8 9 10

Patienten-ID: 2012 - 05 - 24 - 3TP

Fragebogen Teil 1

Liebe Patientin, lieber Patient,

Wir bitten Sie alle nachfolgenden Fragen wahrheitsgemäß zu beantworten.
Bitte kreuzen Sie für jede Frage, sofern nicht anders angegeben, ein Kästchen an!
Bearbeiten Sie zuerst die linke, dann die rechte Spalte.
Bei Fragen stehen wir jederzeit zur Verfügung.

Persönliche Angaben

1. Geburtsdatum: 12.8.66 Alter: 46
2. Geschlecht: ☒ männlich ☐ weiblich
3. Größe: 173
4. Gewicht: 72,4 kg 5. BMI: 24,5
6. Beinigkeit: ☐ links ☒ rechts
7. Beckenumfang: 90 cm

Orthesengröße: ☐ 1 ☒ 2 ☐ 3 ☐ 4

8. Haben sie das Gefühl, dass Sie sich unsicher beim Gehen fühlen, bzw. häufig stürzen?

☐ ja ☒ nein

Angaben zur Schmerzsymptomatik

9. Seit wann bestehen die Schmerzen?

☐ weniger als 1 Monat ☐ 1 Monat bis ½ Jahr
☒ ½ bis 1 Jahr ☐ 1 bis 2 Jahre
☐ 2 bis 5 Jahre ☐ mehr als 5 Jahre

10. Bitte zeichnen Sie ein, an welchen Körperstellen Ihre Schmerzen auftreten

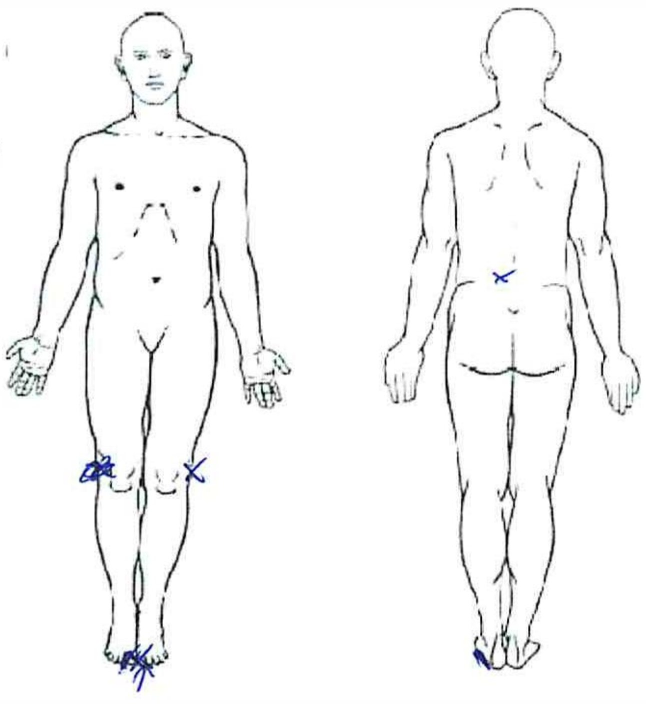

11. Bitte versuchen Sie die Schmerzqualität genauer zu beschreiben, das heißt, wie Sie die Schmerzen typischerweise empfinden. ***Mehrfachnennung ist möglich.***

☐ dumpf ☐ drückend ☐ pochend
☐ klopfend ☒ stechend ☐ ziehend
☐ heiß ☒ brennend ☐ unerträglich

Bitte geben Sie nun die Stärke Ihrer Schmerzen an. Kreuzen Sie dafür auf den Linien die Zahlen an, die Ihrer Schmerzempfindung am nächsten kommen.
0 bedeutet, dass Sie keinerlei Schmerzen haben. Die stärksten Schmerzen, die Sie sich vorstellen können, haben den Zahlenwert 10.
Alle dazwischen liegenden Zahlen sollen Abstufungen darstellen.

*12. Wie schätzen Sie die durchschnittlichen Schmerzen während der **letzten 2 Wochen** ein?*

0 1 2 3 4 5 ☒6 7 8 9 10

*13. Welche Schmerzstärke wäre für Sie bei **erfolgreicher Behandlung** erträglich?*

0 1 ☒2 3 4 5 6 7 8 9 10

*14. Wie stark sind die Schmerzen **im Moment**?*

0 1 2 3 4 5 ☒6 7 8 9 10

Therapie

15. Wie wurden Ihre Schmerzen bereits behandelt?

☒ Krankengymnastik ☐ Massagen
☐ Akupunktur ☐ Kälte-/Wärmetherapie
☒ Einspritzung in das Schmerzgebiet
☒ Medikamente, ggf. welche
☐ andere Behandlungen:

16. Nehmen Sie aktuell Medikamente zur Schmerzreduktion ein?

☒ ja ☐ nein

Wenn ja, geben Sie bitte Präparat und Dosierung an.

Novaminsulfon 500mg
2-2-2

thr

Patienten-ID: 2012 - 09 - 24 - 370 Fragebogen Teil 2

Wir fragen Sie nun nach den Schmerzen, die Sie haben, wenn Sie die Orthese tragen.
Bitte geben Sie die Stärke Ihrer Schmerzen an. Kreuzen Sie dafür auf den Linien die Zahlen an, die Ihrer Schmerzempfindung am nächsten kommen.
0 bedeutet, dass Sie keinerlei Schmerzen haben. Die stärksten Schmerzen, die Sie sich vorstellen können, haben den Zahlenwert 10. Alle dazwischen liegenden Zahlen sollen Abstufungen darstellen.

17. Mit welchem Zahlenwert würden Sie die Schmerzen bei ***leicht gestraffter Orthese*** *beschreiben?*

am Tag: 0 1 2 3 ~~4~~ (X) 5 6 7 8 9 10

früh: X (7)

Abend: X (6)

Patienten-ID: 2012 - 05 - C4 - 37P

Fragebogen Teil 3

Wir fragen Sie nun noch einmal nach den Schmerzen, die Sie haben, wenn Sie die Orthese tragen.
Bitte geben Sie nun die Stärke Ihrer Schmerzen an. Kreuzen Sie dafür auf den Linien die Zahlen an, die Ihrer Schmerzempfindung am nächsten kommen.
0 bedeutet, dass Sie keinerlei Schmerzen haben. Die stärksten Schmerzen, die Sie sich vorstellen können, haben den Zahlenwert 10. Alle dazwischen liegenden Zahlen sollen Abstufungen darstellen.

am Tag
früh
abends

18. Wie stark empfinden Sie die Schmerzen, wenn die ***Orthese maximal gestrafft*** *ist?*

0 1 2 3 (X) 4 5 6 (X) 7 8 (X) 9 10

am Tag
früh
abends

19. Wie stark empfinden Sie die Schmerzen ***nach Abnahme*** *der Orthese?*

0 1 2 (X) 3 4 5 (X) 6 (X) 7 8 9 10

nach Abnahme der Orthese fühle ich mich leichter, aber leider nicht sehr lang.

20. Haben Sie das Gefühl, dass die Orthese selbst Ihnen Schmerzen verursacht? Wenn ja, wie stark sind diese?

0 1 2 3 4 5 6 7 8 9 10

ja, beim Treppen steigen X (6)
ja, wenn die Schmerzspitzen kommen X (8)

Patienten-ID: 2012 - 10 - 15 - 3PP

Fragebogen Teil 1

Liebe Patientin, lieber Patient,

Wir bitten Sie alle nachfolgenden Fragen wahrheitsgemäß zu beantworten.
Bitte kreuzen Sie für jede Frage, sofern nicht anders angegeben, ein Kästchen an!
Bearbeiten Sie zuerst die linke, dann die rechte Spalte.
Bei Fragen stehen wir jederzeit zur Verfügung.

Persönliche Angaben

1. Geburtsdatum: 06.11.57 Alter: 60 J
2. Geschlecht: ☐ männlich ☒ weiblich
3. Größe: 163 cm
4. Gewicht: 68,8 kg 5. BMI: ______
6. Beinigkeit: ☐ links ☒ rechts
7. Beckenumfang: 104 cm
   Orthesengröße: ☐ 1 ☐ 2 ☒ 3 ☐ 4

8. Haben sie das Gefühl, dass Sie sich unsicher beim Gehen fühlen, bzw. häufig stürzen?

☐ ja ☒ nein

Angaben zur Schmerzsymptomatik

9. Seit wann bestehen die Schmerzen?

☐ weniger als 1 Monat ☐ 1 Monat bis ½ Jahr
☐ ½ bis 1 Jahr ☐ 1 bis 2 Jahre
☐ 2 bis 5 Jahre ☒ mehr als 5 Jahre

10. Bitte zeichnen Sie ein, an welchen Körperstellen Ihre Schmerzen auftreten

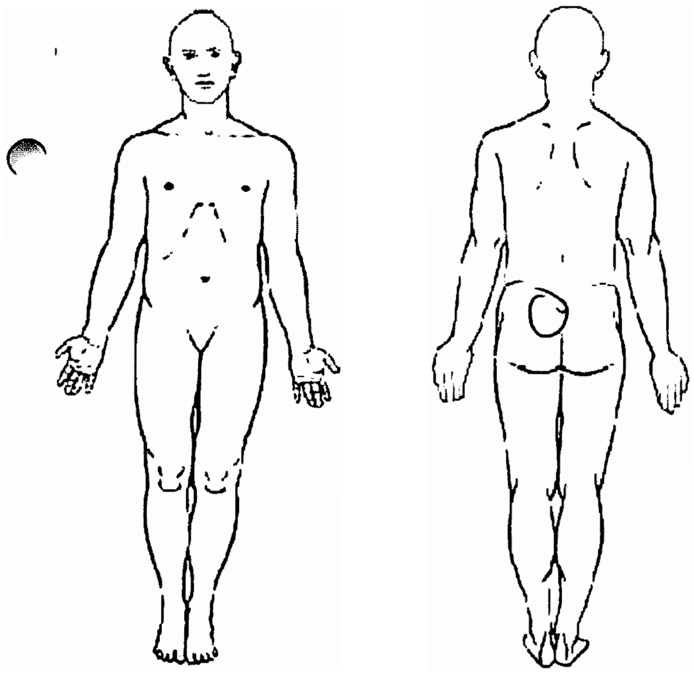

11. Bitte versuchen Sie die Schmerzqualität genauer zu beschreiben, das heißt, wie Sie die Schmerzen typischerweise empfinden. ***Mehrfachnennung ist möglich.***

☐ dumpf ☐ drückend ☐ pochend
☐ klopfend ☐ stechend ☐ ziehend
☐ heiß ☐ brennend ☐ unerträglich

Bitte geben Sie nun die Stärke Ihrer Schmerzen an. Kreuzen Sie dafür auf den Linien die Zahlen an, die Ihrer Schmerzempfindung am nächsten kommen.
0 bedeutet, dass Sie keinerlei Schmerzen haben. Die stärksten Schmerzen, die Sie sich vorstellen können, haben den Zahlenwert 10.
Alle dazwischen liegenden Zahlen sollen Abstufungen darstellen.

*12. Wie schätzen Sie die durchschnittlichen Schmerzen während der **letzten 2 Wochen** ein?*

0 1 2 3 4 5 6 7 8 9 10 (markiert: 4)

*13. Welche Schmerzstärke wäre für Sie bei **erfolgreicher Behandlung** erträglich?*

0 1 2 3 4 5 6 7 8 9 10 (markiert: 2)

*14. Wie stark sind die Schmerzen **im Moment**?*

0 1 2 3 4 5 6 7 8 9 10 (markiert: 3)

Therapie

15. Wie wurden Ihre Schmerzen bereits behandelt?

☐ Krankengymnastik ☐ Massagen
☐ Akupunktur ☐ Kälte-/Wärmetherapie
☒ Einspritzung in das Schmerzgebiet
☒ Medikamente, ggf. welche
☐ andere Behandlungen:
____

16. Nehmen Sie aktuell Medikamente zur Schmerzreduktion ein?

☐ ja ☐ nein

Wenn ja, geben Sie bitte Präparat und Dosierung an.

____
____
____
____
____

Ur

Wir fragen Sie nun nach den Schmerzen, die Sie haben, wenn Sie die Orthese tragen.
Bitte geben Sie die Stärke Ihrer Schmerzen an. Kreuzen Sie dafür auf den Linien die Zahlen an, die Ihrer Schmerzempfindung am nächsten kommen.
0 bedeutet, dass Sie keinerlei Schmerzen haben. Die stärksten Schmerzen, die Sie sich vorstellen können, haben den Zahlenwert 10. Alle dazwischen liegenden Zahlen sollen Abstufungen darstellen.

*17. Mit welchem Zahlenwert würden Sie die Schmerzen bei **leicht gestraffter Orthese** beschreiben?*

0 1 2 3 4 5 6 7 8 9 10

4

Patienten-ID: 2012 - 10 - 15 - 38P Fragebogen Teil 3

Wir fragen Sie nun noch einmal nach den Schmerzen, die Sie haben, wenn Sie die Orthese tragen.
Bitte geben Sie nun die Stärke Ihrer Schmerzen an. Kreuzen Sie dafür auf den Linien die Zahlen an, die Ihrer Schmerzempfindung am nächsten kommen.
0 bedeutet, dass Sie keinerlei Schmerzen haben. Die stärksten Schmerzen, die Sie sich vorstellen können, haben den Zahlenwert 10. Alle dazwischen liegenden Zahlen sollen Abstufungen darstellen.

*18. Wie stark empfinden Sie die Schmerzen, wenn die **Orthese maximal gestrafft** ist?*

0 1 2 3 4 5 6 7 8 9 10

*19. Wie stark empfinden Sie die Schmerzen **nach Abnahme** der Orthese?*

0 1 2 3 4 5 6 7 8 9 10

20. Haben Sie das Gefühl, dass die Orthese selbst Ihnen Schmerzen verursacht? Wenn ja, wie stark sind diese?

0 1 2 3 4 5 6 7 8 9 10

Patienten-ID: 2012 - 10 - 22 - 390 Fragebogen Teil 1

Liebe Patientin, lieber Patient,

Wir bitten Sie alle nachfolgenden Fragen wahrheitsgemäß zu beantworten.
Bitte kreuzen Sie für jede Frage, sofern nicht anders angegeben, ein Kästchen an!
Bearbeiten Sie zuerst die linke, dann die rechte Spalte.
Bei Fragen stehen wir jederzeit zur Verfügung.

Persönliche Angaben

1. Geburtsdatum: 30.07.70 Alter: 42
2. Geschlecht: ☐ männlich ☒ weiblich
3. Größe: 175
4. Gewicht: 74 5. BMI: ____
6. Beinigkeit: ☒ links ☐ rechts
7. Beckenumfang: 94 cm
   Orthesengröße: ☐ 1 ☒ 2 ☐ 3 ☐ 4

8. Haben sie das Gefühl, dass Sie sich unsicher beim Gehen fühlen, bzw. häufig stürzen?
   ☐ ja ☒ nein

Angaben zur Schmerzsymptomatik

9. Seit wann bestehen die Schmerzen?

☐ weniger als 1 Monat ☐ 1 Monat bis ½ Jahr
☐ ½ bis 1 Jahr ☐ 1 bis 2 Jahre
☐ 2 bis 5 Jahre ☒ mehr als 5 Jahre

10. Bitte zeichnen Sie ein, an welchen Körperstellen Ihre Schmerzen auftreten

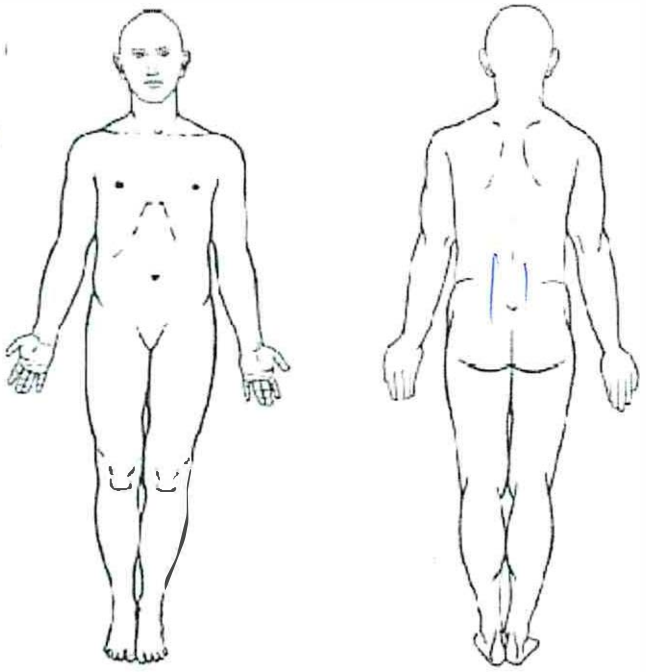

11. Bitte versuchen Sie die Schmerzqualität genauer zu beschreiben, das heißt, wie Sie die Schmerzen typischerweise empfinden. ***Mehrfachnennung ist möglich.***

☒ dumpf ☒ drückend ☐ pochend
☐ klopfend ☐ stechend ☒ ziehend
☐ heiß ☐ brennend ☐ unerträglich

Bitte geben Sie nun die Stärke Ihrer Schmerzen an. Kreuzen Sie dafür auf den Linien die Zahlen an, die Ihrer Schmerzempfindung am nächsten kommen.
0 bedeutet, dass Sie keinerlei Schmerzen haben. Die stärksten Schmerzen, die Sie sich vorstellen können, haben den Zahlenwert 10.
Alle dazwischen liegenden Zahlen sollen Abstufungen darstellen.

*12. Wie schätzen Sie die durchschnittlichen Schmerzen während der **letzten 2 Wochen** ein?*

0 1 2 3 4 5 6 7 8 9 10 (Kreuz zwischen 3 und 4)

*13. Welche Schmerzstärke wäre für Sie bei **erfolgreicher Behandlung** erträglich?*

0 1 2 3 4 5 6 7 8 9 10 (Kreuz zwischen 1 und 2)

*14. Wie stark sind die Schmerzen **im Moment**?*

0 1 2 3 4 5 6 7 8 9 10 (Kreuz zwischen 2 und 3)

Therapie

15. Wie wurden Ihre Schmerzen bereits behandelt?

☒ Krankengymnastik ☐ Massagen
☐ Akupunktur ☒ Kälte-/Wärmetherapie
☒ Einspritzung in das Schmerzgebiet (NT)
☒ Medikamente, ggf. welche Ibu
☒ andere Behandlungen:
Chirotherapie

16. Nehmen Sie aktuell Medikamente zur Schmerzreduktion ein?
   ☐ ja ☒ nein

Wenn ja, geben Sie bitte Präparat und Dosierung an.

____
____
____
____
____

Wir fragen Sie nun nach den Schmerzen, die Sie haben, wenn Sie die Orthese tragen.
Bitte geben Sie die Stärke Ihrer Schmerzen an. Kreuzen Sie dafür auf den Linien die Zahlen an, die Ihrer Schmerzempfindung am nächsten kommen.
0 bedeutet, dass Sie keinerlei Schmerzen haben. Die stärksten Schmerzen, die Sie sich vorstellen können, haben den Zahlenwert 10. Alle dazwischen liegenden Zahlen sollen Abstufungen darstellen.

*17. Mit welchem Zahlenwert würden Sie die Schmerzen bei **leicht gestraffter Orthese** beschreiben?*

0 1 ~~2~~ X 3 4 5 6 7 8 9 10

Wir fragen Sie nun noch einmal nach den Schmerzen, die Sie haben, wenn Sie die Orthese tragen.
Bitte geben Sie nun die Stärke Ihrer Schmerzen an. Kreuzen Sie dafür auf den Linien die Zahlen an, die Ihrer Schmerzempfindung am nächsten kommen.
0 bedeutet, dass Sie keinerlei Schmerzen haben. Die stärksten Schmerzen, die Sie sich vorstellen können, haben den Zahlenwert 10. Alle dazwischen liegenden Zahlen sollen Abstufungen darstellen.

*18. Wie stark empfinden Sie die Schmerzen, wenn die **Orthese maximal gestrafft** ist?*

0 1 2 X 3 4 5 6 7 8 9 10

*19. Wie stark empfinden Sie die Schmerzen **nach Abnahme** der Orthese?*

0 1 X 2 3 4 5 6 7 8 9 10

20. Haben Sie das Gefühl, dass die Orthese selbst Ihnen Schmerzen verursacht? Wenn ja, wie stark sind diese?

0 1 2 3 4 5 6 7 8 9 10 ø

Patienten-ID: 2012 - 11 - 05 - 40P

Fragebogen Teil 1

Liebe Patientin, lieber Patient,

Wir bitten Sie alle nachfolgenden Fragen wahrheitsgemäß zu beantworten.
Bitte kreuzen Sie für jede Frage, sofern nicht anders angegeben, ein Kästchen an!
Bearbeiten Sie zuerst die linke, dann die rechte Spalte.
Bei Fragen stehen wir jederzeit zur Verfügung.

Persönliche Angaben

1. Geburtsdatum: 17.08.85 Alter: 27
2. Geschlecht: ☒ männlich ☐ weiblich
3. Größe: 184
4. Gewicht: 71 5. BMI: ________
6. Beinigkeit: ☐ links ☒ rechts
7. Beckenumfang: 86 cm
   Orthesengröße: ☒ 1 ☐ 2 ☐ 3 ☐ 4

8. Haben sie das Gefühl, dass Sie sich unsicher beim Gehen fühlen, bzw. häufig stürzen?
   ☐ ja ☒ nein

Angaben zur Schmerzsymptomatik

9. Seit wann bestehen die Schmerzen?

☐ weniger als 1 Monat ☒ 1 Monat bis ½ Jahr
☐ ½ bis 1 Jahr ☐ 1 bis 2 Jahre
☐ 2 bis 5 Jahre ☐ mehr als 5 Jahre

10. Bitte zeichnen Sie ein, an welchen Körperstellen Ihre Schmerzen auftreten

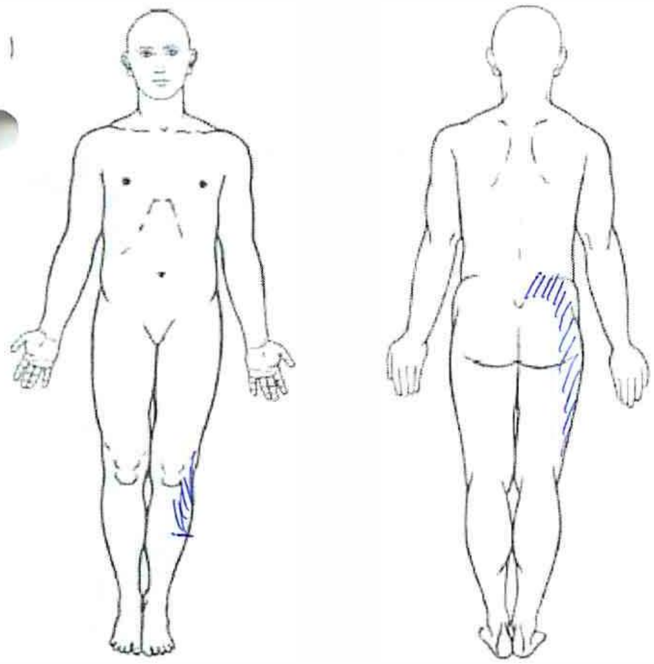

11. Bitte versuchen Sie die Schmerzqualität genauer zu beschreiben, das heißt, wie Sie die Schmerzen typischerweise empfinden. ***Mehrfachnennung ist möglich.***

☒ dumpf ☐ drückend ☐ pochend
☐ klopfend ☐ stechend ☒ ziehend
☐ heiß ☐ brennend ☒ unerträglich
initial

Bitte geben Sie nun die Stärke Ihrer Schmerzen an. Kreuzen Sie dafür auf den Linien die Zahlen an, die Ihrer Schmerzempfindung am nächsten kommen.
0 bedeutet, dass Sie keinerlei Schmerzen haben. Die stärksten Schmerzen, die Sie sich vorstellen können, haben den Zahlenwert 10. Alle dazwischen liegenden Zahlen sollen Abstufungen darstellen.

12. Wie schätzen Sie die durchschnittlichen Schmerzen während ***der letzten 2 Wochen*** *ein?*

0 1 2 (3) 4 5 6 7 8 9 10

13. Welche Schmerzstärke wäre für Sie bei ***erfolgreicher Behandlung*** *erträglich?*

0 (1) 2 3 4 5 6 7 8 9 10

14. Wie stark sind die Schmerzen ***im Moment****?*

0 1 (2) 3 4 5 6 7 8 9 10

Therapie

15. Wie wurden Ihre Schmerzen bereits behandelt?

☒ Krankengymnastik ■ Massagen
☐ Akupunktur ☒ Kälte-/Wärmetherapie → Sauna
☒ Einspritzung in das Schmerzgebiet
☒ Medikamente, ggf. welche Diclofenac + Dexamethason
☐ andere Behandlungen: ____________________

16. Nehmen Sie aktuell Medikamente zur Schmerzreduktion ein?
   ☐ ja ☒ nein

Wenn ja, geben Sie bitte Präparat und Dosierung an.

____
____
____
____
____

Wir fragen Sie nun nach den Schmerzen, die Sie haben, wenn Sie die Orthese tragen.
Bitte geben Sie die Stärke Ihrer Schmerzen an. Kreuzen Sie dafür auf den Linien die Zahlen an, die Ihrer Schmerzempfindung am nächsten kommen.
0 bedeutet, dass Sie keinerlei Schmerzen haben. Die stärksten Schmerzen, die Sie sich vorstellen können, haben den Zahlenwert 10. Alle dazwischen liegenden Zahlen sollen Abstufungen darstellen.

17. Mit welchem Zahlenwert würden Sie die Schmerzen bei ***leicht gestraffter Orthese*** *beschreiben?*

0 1 (2) 3 4 5 6 7 8 9 10

Patienten-ID: 2012 - 11 - 05 - 40P Fragebogen Teil 3

Wir fragen Sie nun noch einmal nach den Schmerzen, die Sie haben, wenn Sie die Orthese tragen. Bitte geben Sie nun die Stärke Ihrer Schmerzen an. Kreuzen Sie dafür auf den Linien die Zahlen an, die Ihrer Schmerzempfindung am nächsten kommen.
0 bedeutet, dass Sie keinerlei Schmerzen haben. Die stärksten Schmerzen, die Sie sich vorstellen können, haben den Zahlenwert 10. Alle dazwischen liegenden Zahlen sollen Abstufungen darstellen.

*18. Wie stark empfinden Sie die Schmerzen, wenn die **Orthese maximal gestrafft** ist?*

0 (1) 2 3 4 5 6 7 8 9 10

*19. Wie stark empfinden Sie die Schmerzen **nach Abnahme** der Orthese?*

0 1 (2) 3 4 5 6 7 8 9 10

20. Haben Sie das Gefühl, dass die Orthese selbst Ihnen Schmerzen verursacht? Wenn ja, wie stark sind diese?

0 (1) 2 3 4 5 6 7 8 9 10

↳ bei viel Bewegung => Reibung

Patienten-ID: 2012 - 11 - 12 - 41P

Fragebogen Teil 1

Liebe Patientin, lieber Patient,

Wir bitten Sie alle nachfolgenden Fragen wahrheitsgemäß zu beantworten.
Bitte kreuzen Sie für jede Frage, sofern nicht anders angegeben, ein Kästchen an!
Bearbeiten Sie zuerst die linke, dann die rechte Spalte.
Bei Fragen stehen wir jederzeit zur Verfügung.

Persönliche Angaben

1. Geburtsdatum: 30.08.74 Alter: 38
2. Geschlecht: ☐ männlich ☒ weiblich
3. Größe: 164 cm
4. Gewicht: 83,7 kg 5. BMI: ______
6. Beinigkeit: ☐ links ☒ rechts
7. Beckenumfang: 104 cm
   Orthesengröße: ☐ 1 ☐ 2 ☒ 3 ☐ 4

8. Haben sie das Gefühl, dass Sie sich unsicher beim Gehen fühlen, bzw. häufig stürzen?
   ☐ ja ☒ nein

Angaben zur Schmerzsymptomatik

9. Seit wann bestehen die Schmerzen?

☐ weniger als 1 Monat
☐ ½ bis 1 Jahr
☐ 2 bis 5 Jahre
☐ 1 Monat bis ½ Jahr
☒ 1 bis 2 Jahre
☐ mehr als 5 Jahre

10. Bitte zeichnen Sie ein, an welchen Körperstellen Ihre Schmerzen auftreten

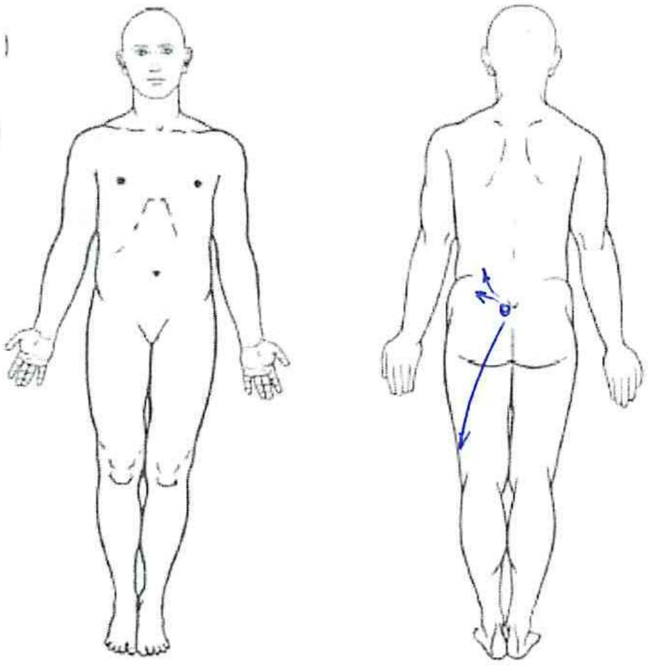

11. Bitte versuchen Sie die Schmerzqualität genauer zu beschreiben, das heißt, wie Sie die Schmerzen typischerweise empfinden. ***Mehrfachnennung ist möglich.***

☐ dumpf ☒ drückend ☐ pochend
☐ klopfend ☐ stechend ☐ ziehend
☒ heiß ☒ brennend ☐ unerträglich

Bitte geben Sie nun die Stärke Ihrer Schmerzen an. Kreuzen Sie dafür auf den Linien die Zahlen an, die Ihrer Schmerzempfindung am nächsten kommen.
0 bedeutet, dass Sie keinerlei Schmerzen haben. Die stärksten Schmerzen, die Sie sich vorstellen können, haben den Zahlenwert 10. Alle dazwischen liegenden Zahlen sollen Abstufungen darstellen.

12. *Wie schätzen Sie die durchschnittlichen Schmerzen während der **letzten 2 Wochen** ein?*

0 1 2 3 4 5 6 7 8 9 10

13. *Welche Schmerzstärke wäre für Sie bei **erfolgreicher Behandlung** erträglich?*

0 1 2 3 4 5 6 7 8 9 10

14. *Wie stark sind die Schmerzen **im Moment**?*

0 1 2 3 4 5 6 7 8 9 10

Therapie

15. Wie wurden Ihre Schmerzen bereits behandelt?

☒ Krankengymnastik
☐ Akupunktur
☒ Einspritzung in das Schmerzgebiet
☒ Medikamente, ggf. welche
☐ andere Behandlungen: ______
☒ Massagen
☐ Kälte-/Wärmetherapie

16. Nehmen Sie aktuell Medikamente zur Schmerzreduktion ein?
   ☒ ja ☐ nein

Wenn ja, geben Sie bitte Präparat und Dosierung an. ?

Wir fragen Sie nun nach den Schmerzen, die Sie haben, wenn Sie die Orthese tragen.
Bitte geben Sie die Stärke Ihrer Schmerzen an. Kreuzen Sie dafür auf den Linien die Zahlen an, die Ihrer Schmerzempfindung am nächsten kommen.
0 bedeutet, dass Sie keinerlei Schmerzen haben. Die stärksten Schmerzen, die Sie sich vorstellen können, haben den Zahlenwert 10. Alle dazwischen liegenden Zahlen sollen Abstufungen darstellen.

*17. Mit welchem Zahlenwert würden Sie die Schmerzen bei **leicht gestraffter Orthese** beschreiben?*

0 1 2 3 4 5 6 7 8 9 10

Patienten-ID: 2012 - 12 - 12 - 47P Fragebogen Teil 3

Wir fragen Sie nun noch einmal nach den Schmerzen, die Sie haben, wenn Sie die Orthese tragen.
Bitte geben Sie nun die Stärke Ihrer Schmerzen an. Kreuzen Sie dafür auf den Linien die Zahlen an, die Ihrer Schmerzempfindung am nächsten kommen.
0 bedeutet, dass Sie keinerlei Schmerzen haben. Die stärksten Schmerzen, die Sie sich vorstellen können, haben den Zahlenwert 10. Alle dazwischen liegenden Zahlen sollen Abstufungen darstellen.

*18. Wie stark empfinden Sie die Schmerzen, wenn die **Orthese maximal gestrafft** ist?*

0 1 2 3 4 5 ~~6~~ X 7 8 9 10

*19. Wie stark empfinden Sie die Schmerzen **nach Abnahme** der Orthese?*

0 1 2 3 4 5 6 X 8 9 10

20. Haben Sie das Gefühl, dass die Orthese selbst Ihnen Schmerzen verursacht? Wenn ja, wie stark sind diese? nein

0 1 2 3 4 5 6 7 8 9 10

Patienten-ID: 2012 - 12 - 03 - 42P

Fragebogen Teil 1

Liebe Patientin, lieber Patient,

Wir bitten Sie alle nachfolgenden Fragen wahrheitsgemäß zu beantworten.
Bitte kreuzen Sie für jede Frage, sofern nicht anders angegeben, ein Kästchen an!
Bearbeiten Sie zuerst die linke, dann die rechte Spalte.
Bei Fragen stehen wir jederzeit zur Verfügung.

Persönliche Angaben

1. Geburtsdatum: 16.05.58 Alter: 54
2. Geschlecht: ☐ männlich ☒ weiblich
3. Größe: 168 cm
4. Gewicht: 71,8 kg 5. BMI: ________
6. Beinigkeit: ☐ links ☒ rechts
7. Beckenumfang: 101 cm

Orthesengröße: ☐ 1 ☐ 2 ☒ 3 ☐ 4

8. Haben sie das Gefühl, dass Sie sich unsicher beim Gehen fühlen, bzw. häufig stürzen?

☐ ja ☒ nein

Angaben zur Schmerzsymptomatik

9. Seit wann bestehen die Schmerzen?

☐ weniger als 1 Monat ☐ 1 Monat bis ½ Jahr
☐ ½ bis 1 Jahr ☐ 1 bis 2 Jahre
☐ 2 bis 5 Jahre ☒ mehr als 5 Jahre

10. Bitte zeichnen Sie ein, an welchen Körperstellen Ihre Schmerzen auftreten

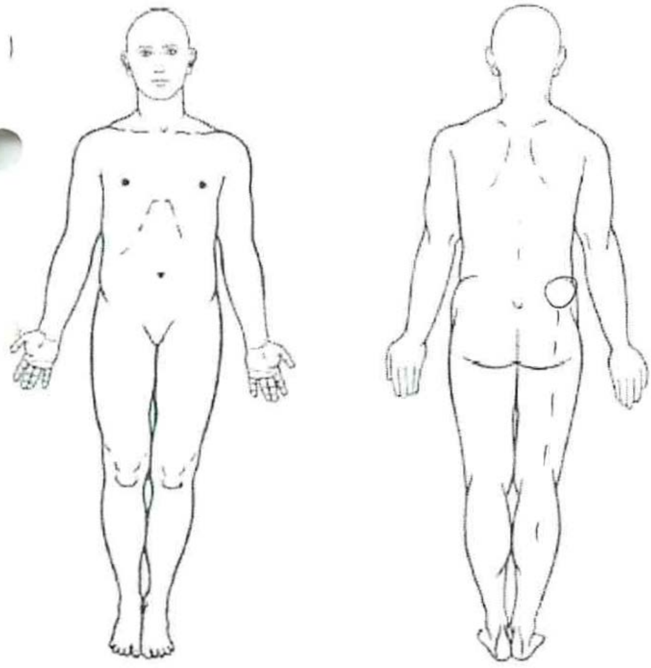

11. Bitte versuchen Sie die Schmerzqualität genauer zu beschreiben, das heißt, wie Sie die Schmerzen typischerweise empfinden. ***Mehrfachnennung ist möglich.***

☐ dumpf ☐ drückend ☐ pochend
☐ klopfend ☐ stechend ☒ ziehend
☐ heiß ☐ brennend ☐ unerträglich

Bitte geben Sie nun die Stärke Ihrer Schmerzen an. Kreuzen Sie dafür auf den Linien die Zahlen an, die Ihrer Schmerzempfindung am nächsten kommen.
0 bedeutet, dass Sie keinerlei Schmerzen haben. Die stärksten Schmerzen, die Sie sich vorstellen können, haben den Zahlenwert 10. Alle dazwischen liegenden Zahlen sollen Abstufungen darstellen.

*12. Wie schätzen Sie die durchschnittlichen Schmerzen während der **letzten 2 Wochen** ein?*

0 1 2 3 4 5 (X) 6 7 8 9 10

*13. Welche Schmerzstärke wäre für Sie bei **erfolgreicher Behandlung** erträglich?*

0 1 2 3 (X) 4 5 6 7 8 9 10

*14. Wie stark sind die Schmerzen **im Moment?***

0 1 2 3 4 5 (X) 6 7 8 9 10

Therapie

15. Wie wurden Ihre Schmerzen bereits behandelt?

☒ Krankengymnastik ☐ Massagen
☐ Akupunktur ☒ Kälte-/Wärmetherapie
☐ Einspritzung in das Schmerzgebiet
☒ Medikamente, ggf. welche Fentanyl Pfl.
☐ andere Behandlungen:

16. Nehmen Sie aktuell Medikamente zur Schmerzreduktion ein?

☒ ja ☐ nein

Wenn ja, geben Sie bitte Präparat und Dosierung an.

Fentanyl Pfl. 12,5 µg
Lyrica 75 0-0-1

Wir fragen Sie nun nach den Schmerzen, die Sie haben, wenn Sie die Orthese tragen.
Bitte geben Sie die Stärke Ihrer Schmerzen an. Kreuzen Sie dafür auf den Linien die Zahlen an, die Ihrer Schmerzempfindung am nächsten kommen.
0 bedeutet, dass Sie keinerlei Schmerzen haben. Die stärksten Schmerzen, die Sie sich vorstellen können, haben den Zahlenwert 10. Alle dazwischen liegenden Zahlen sollen Abstufungen darstellen.

*17. Mit welchem Zahlenwert würden Sie die Schmerzen bei **leicht gestraffter Orthese** beschreiben?*

X (marked at 4)

0 1 2 3 4 5 6 7 8 9 10

Patienten-ID: 2012 - 12 - 03 - 42P

Wir fragen Sie nun noch einmal nach den Schmerzen, die Sie haben, wenn Sie die Orthese tragen.
Bitte geben Sie nun die Stärke Ihrer Schmerzen an. Kreuzen Sie dafür auf den Linien die Zahlen an, die Ihrer Schmerzempfindung am nächsten kommen.
0 bedeutet, dass Sie keinerlei Schmerzen haben. Die stärksten Schmerzen, die Sie sich vorstellen können, haben den Zahlenwert 10. Alle dazwischen liegenden Zahlen sollen Abstufungen darstellen.

*18. Wie stark empfinden Sie die Schmerzen, wenn die **Orthese maximal gestrafft** ist?*

0 1 2 3 ~~4~~ X 5 6 7 8 9 10

*19. Wie stark empfinden Sie die Schmerzen **nach Abnahme** der Orthese?*

0 1 2 3 4 5 ~~6~~ X 7 8 9 10

20. Haben Sie das Gefühl, dass die Orthese selbst Ihnen Schmerzen verursacht? Wenn ja, wie stark sind diese?

0 1 2 3 4 5 6 7 8 9 10
