# Supplementary figures and images for: Pelvic Belt Effects on Pelvic Morphometry, Muscle Activity and Body Balance in Patients with Sacroiliac Joint Dysfunction
Source: PLoS One. 2015 Mar 17;10(3):e0116739. doi: 10.1371/journal.pone.0116739 (PMC4364533; doi:10.1371/journal.pone.0116739)

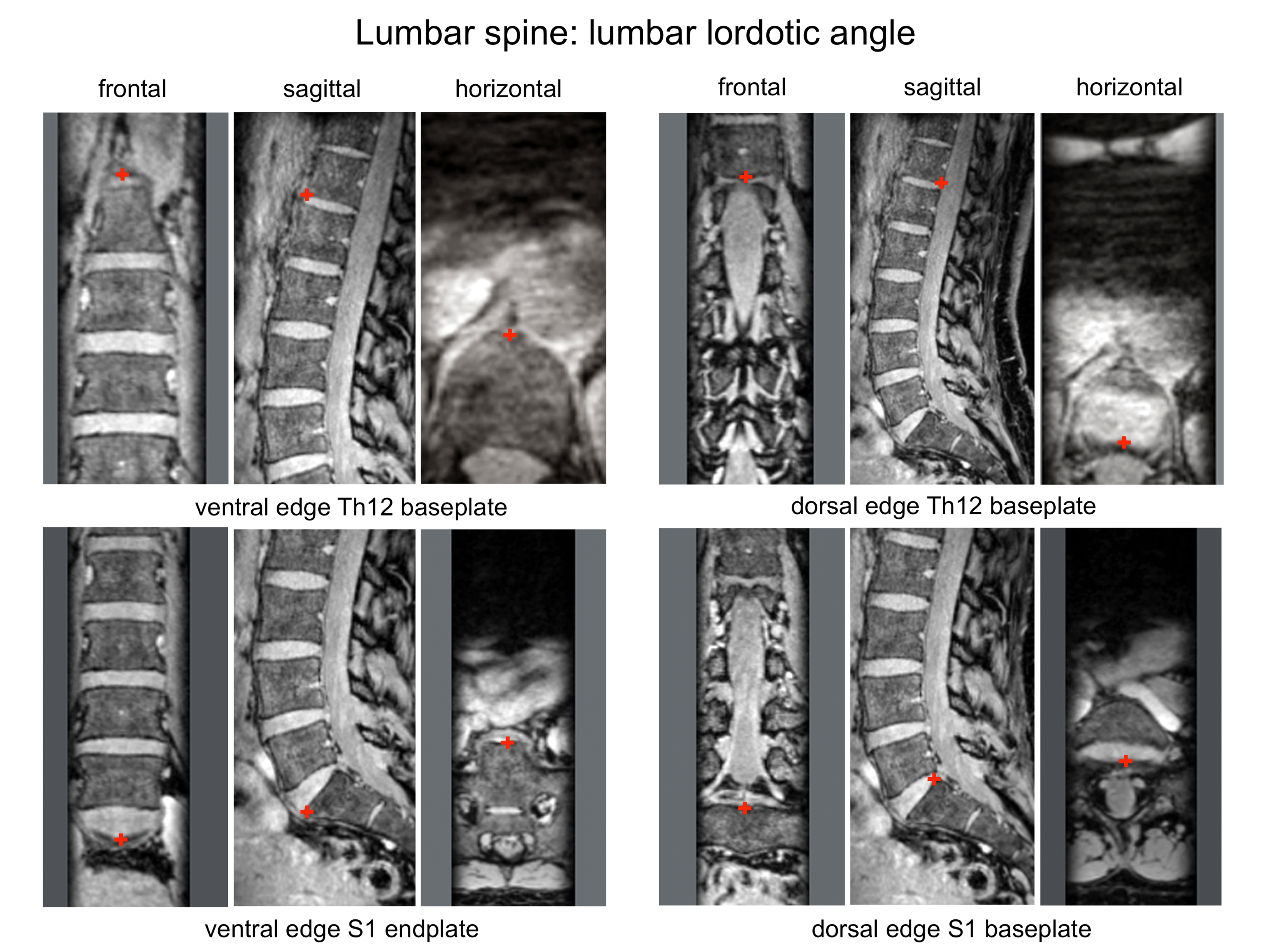

Supplement: S1 Fig — Each of the landmarks was checked in all standard anatomical planes. The lumbar lordotic angle was defined by the intersection of two lines in the median sagittal plane. One line represented the lower twelfth thoracic vertebra surface (baseplate) and the other line represented the upper first sacral vertebra surface (endplate). Each line consisted of a landmark at the ventral and the dorsal edge at the respective vertebra. (TIF) [file pone.0116739.s002.tif]

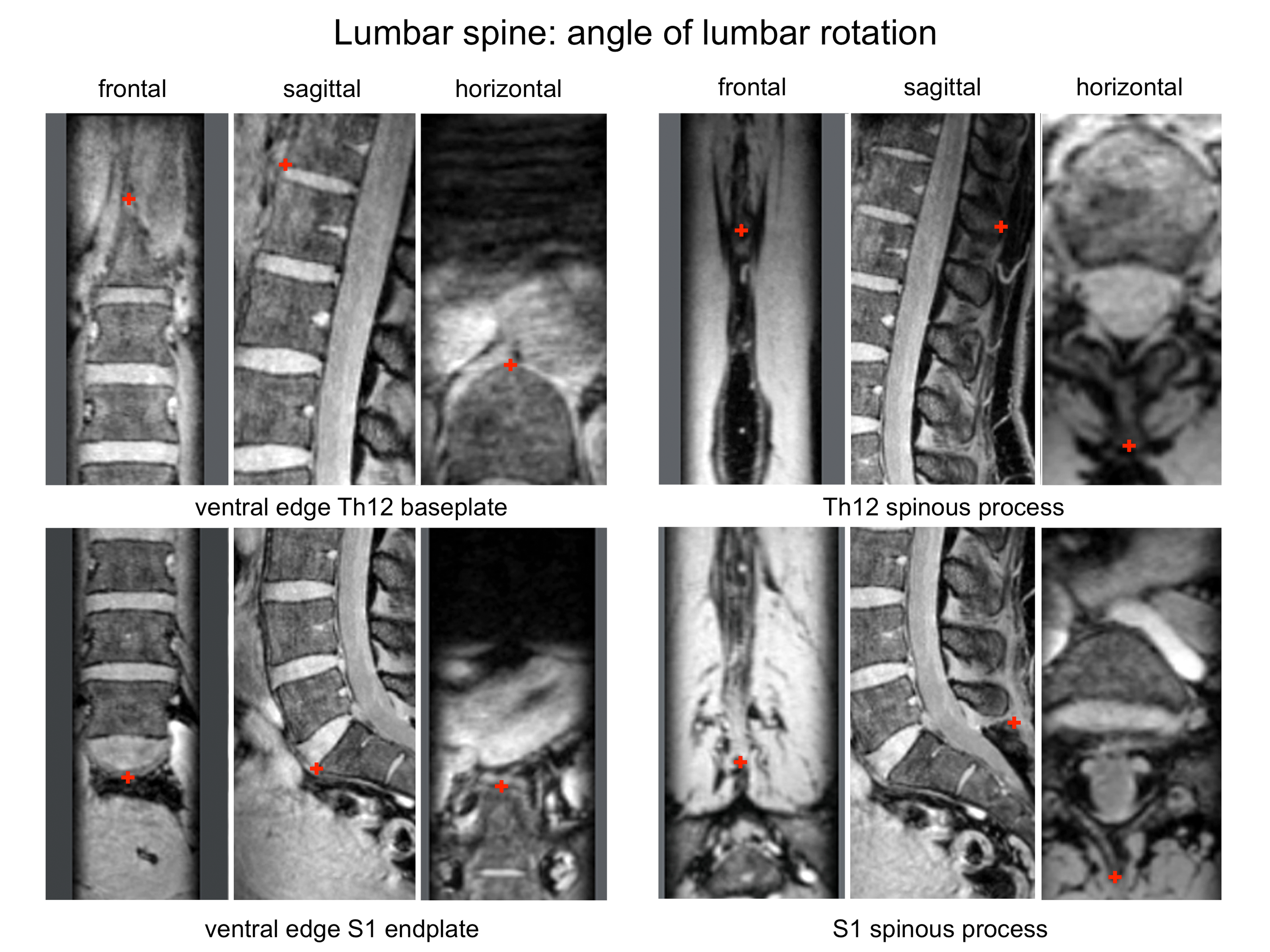

Supplement: S2 Fig — Each of the landmarks was checked in all standard anatomical planes. The angle of lumbar rotation was defined by the intersection of two lines from the baseplate of the twelfth thoracic vertebra and the endplate of the first sacral vertebra in the horizontal plane. Each line consisted of a landmark at the ventral edge and the spinous process of the respective vertebra. (TIF) [file pone.0116739.s003.tif]

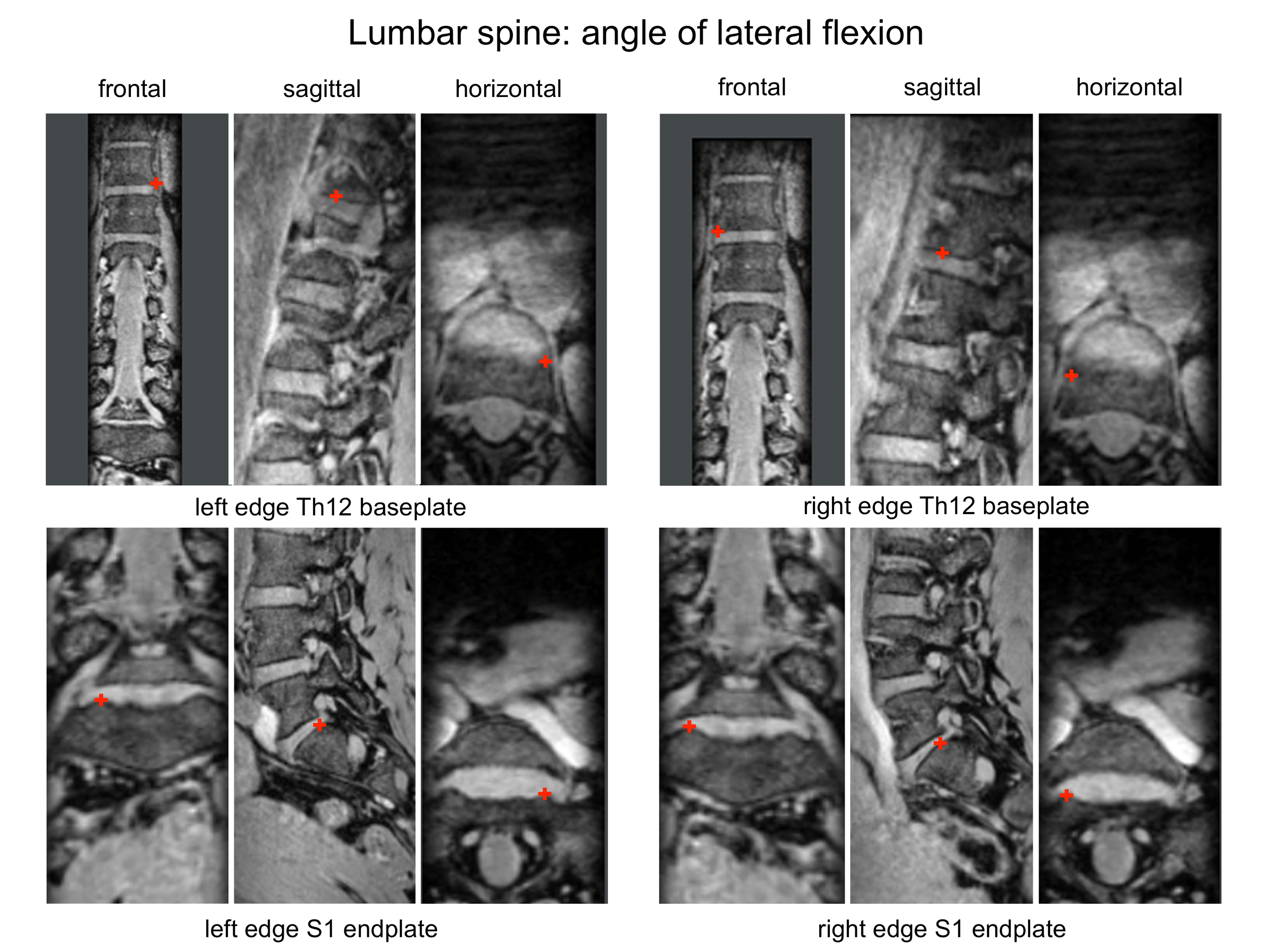

Supplement: S3 Fig — Each of the landmarks was checked in all standard anatomical planes. The angle of lateral flexion was defined by the intersection of a line at the twelfth thoracic vertebra baseplate with a line at the first sacral vertebra endplate in the frontal plane. Both lines consisted of two points set at the lateral edges of each respective vertebral body. (TIF) [file pone.0116739.s004.tif]

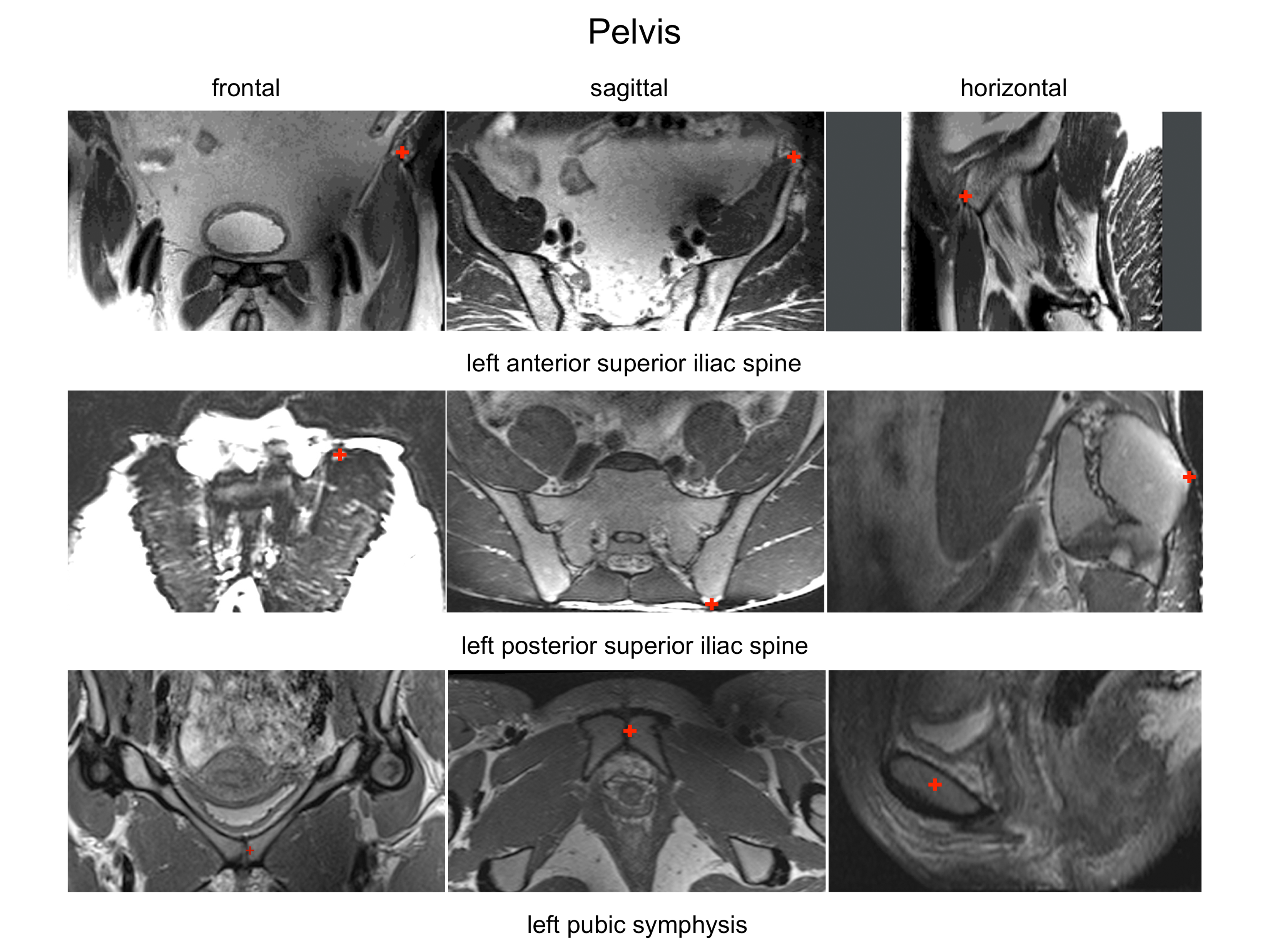

Supplement: S4 Fig — Each of the landmarks was checked in all standard anatomical planes. The anterior and superior iliac spine and the pubic symphysis are marked at the pelvis. (TIF) [file pone.0116739.s005.tif]

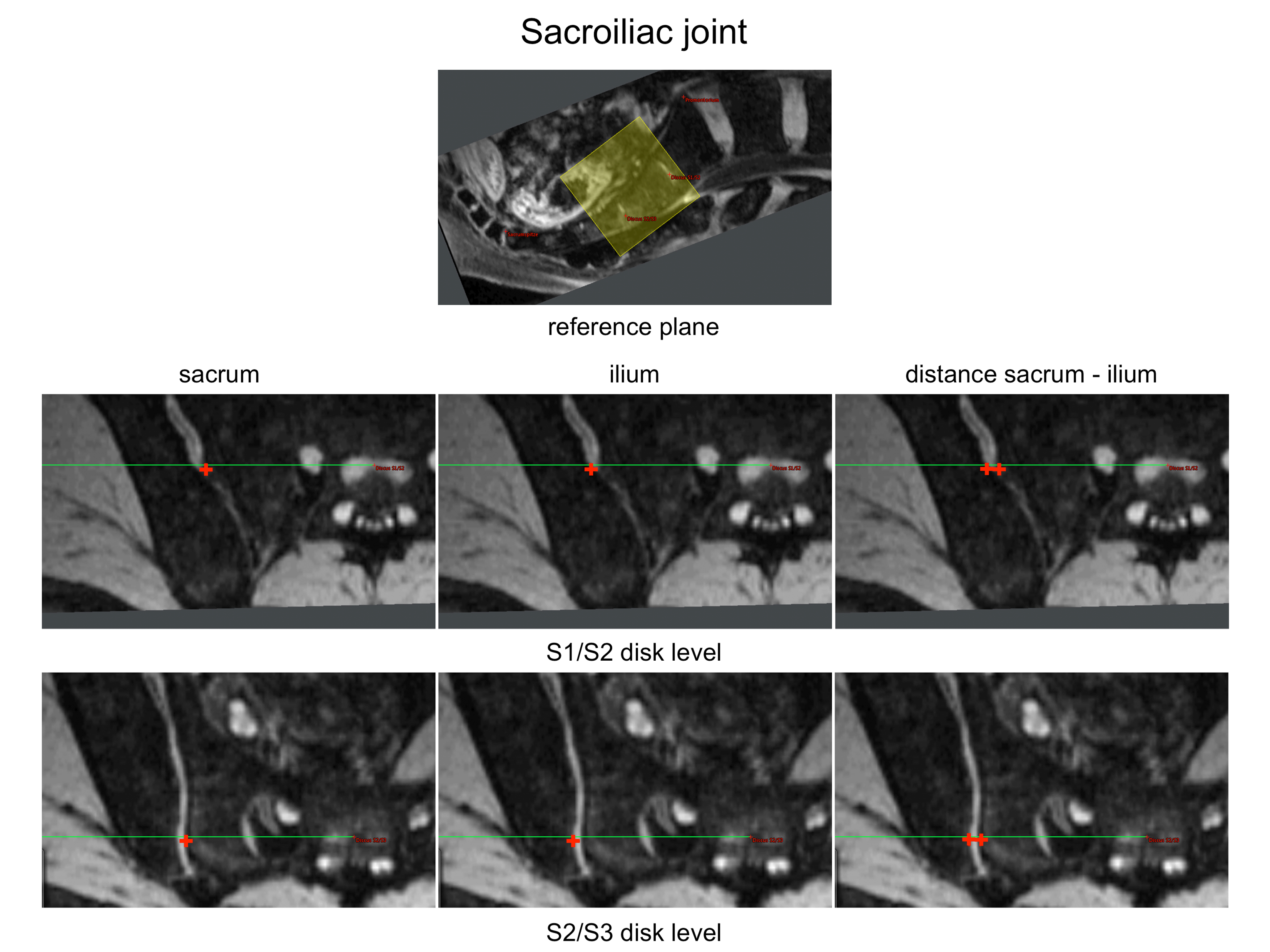

Supplement: S5 Fig — Each of the landmarks was checked in all standard anatomical planes. A reference plane was created with three landmarks: the ventral edge of the first sacral vertebra endplate (promontory), the first sacral vertebra spinous process and the caudal tip of the fifth sacral vertebra. Two lines indicated in green perpendicular to this plane were set at the S1-S2 and S2-S3 disk level. At this line, landmarks were set on the sacral and the iliac side of the sacroiliac joint, measuring the cartilage and joint cavity to full extent. (TIF) [file pone.0116739.s006.tif]

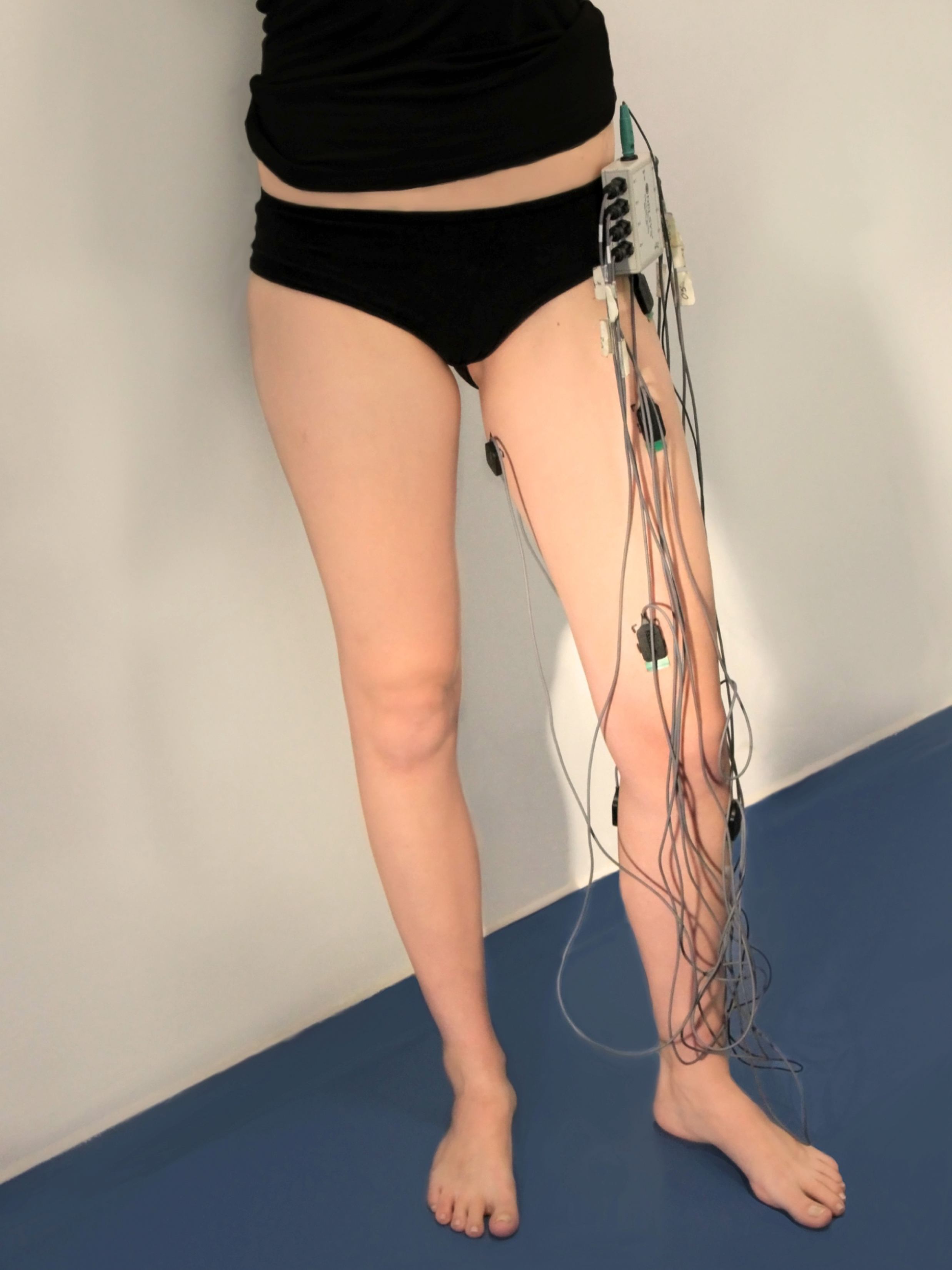

Supplement: S6 Fig — The electrodes were positioned on the adductor magnus, the rectus femoris, the medial vastus, the tibialis anterior. (TIF) [file pone.0116739.s007.tif]

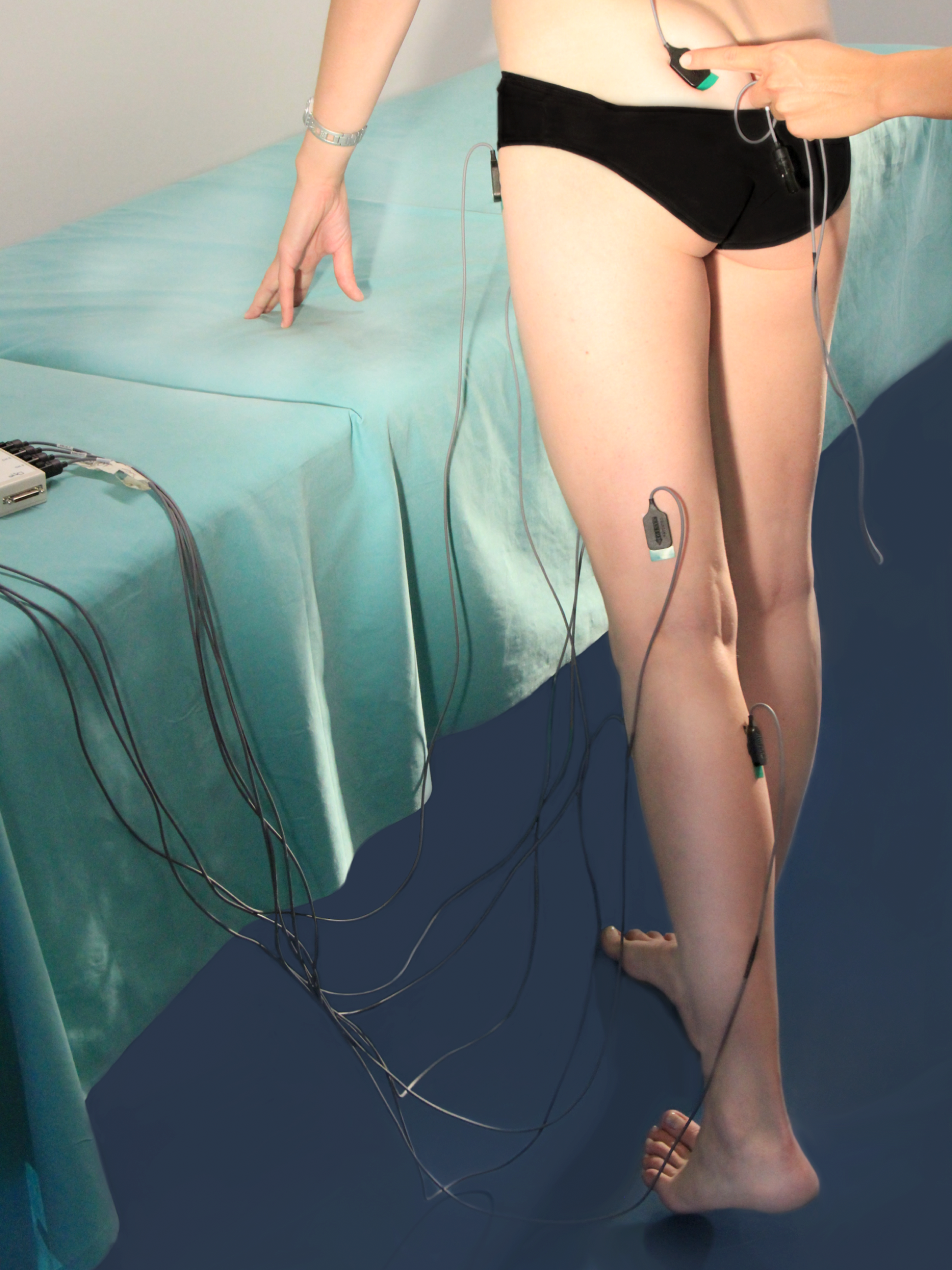

Supplement: S7 Fig — The electrodes were positioned on the gluteus maximus, the tensor fasciae latae, the biceps femoris and the (medial) gastrocnemius. (TIF) [file pone.0116739.s008.tif]
