# Supplementary material for: Pelvic Belt Effects on Pelvic Morphometry, Muscle Activity and Body Balance in Patients with Sacroiliac Joint Dysfunction
Source: PLoS One. 2015 Mar 17;10(3):e0116739. doi: 10.1371/journal.pone.0116739 (PMC4364533; doi:10.1371/journal.pone.0116739)
Supplement: S1 Table — Means and bias of the Bland-Altman analyses are given. ASIS = anterior superior iliac spine, PSIS = posterior superior iliac spine, S1 (2, 3) = first (second, third) sacral vertebral body. A: Between-group comparison of SIJ patients and healthy controls. B: Within-group comparison of the SIJ patient group and the control group measurements from two raters (DOCX) [file pone.0116739.s009.docx]

**Supplement tables**

*S1 Table:* Comparison of MRI-based measurement agreement of lumbar spine, pelvis and sacroiliac joint (SIJ) morphometries on basis of Bland-Altman blots without pelvic belt, under moderate and maximum tension. Means and bias of the Bland-Altman analyses are given. ASIS = anterior superior iliac spine, PSIS = posterior superior iliac spine, S1 (2, 3) = first (second, third) sacral vertebral body

*S1A:* Between-group comparison of SIJ patients and healthy controls

|  |  | **No belt** | | | | **Moderate tension** | | | | **Maximum tension** | | | |
| --- | --- | --- | --- | --- | --- | --- | --- | --- | --- | --- | --- | --- | --- |
|  |  | **SIJ patients** | | **controls** | | **SIJ patients** | | **controls** | | **SIJ patients** | | **controls** | |
|  | | **mean** | **bias** | **mean** | **bias** | **mean** | **bias** | **mean** | **bias** | **mean** | **bias** | **mean** | **bias** |
|  |  |  |  |  |  |  |  |  |  |  |  |  |  |
| **Lumbar spine** | |  |  |  |  |  |  |  |  |  |  |  |  |
| angle | lateral flexion | -0.19 | 4.44 | 0.13 | 4.27 | not recorded | | | | not recorded | | | |
| [°] | lumbar rotation | -0.30 | 3.09 | -0.54 | 3.26 |  |  |  |  |  |  |  |  |
|  | lumbar lordotic | -0.20 | 5.19 | 0.42 | 4.34 |  |  |  |  |  |  |  |  |
|  |  |  |  |  |  |  |  |  |  |  |  |  |  |
| **Pelvis** | |  |  |  |  |  |  |  |  |  |  |  |  |
| distance | ASIS left - ASIS right | 0.16 | 2.18 | 0.67 | 3.97 | 1.42 | 3.15 | -0.11 | 3.99 | 0.56 | 2.88 | -1.14 | 6.51 |
| [mm] | PSIS left - PSIS right | -0.30 | 3.70 | -0.19 | 2.65 | -1.16 | 3.48 | 0.02 | 3.68 | -0.38 | 4.30 | 1.27 | 4.80 |
|  | symphysis left - right | -80.63 | 22.98 | -79.50 | 23.15 | -79.73 | 20.95 | -79.68 | 21.48 | -79.61 | 23.88 | -79.50 | 23.32 |
|  | ASIS - PSIS left | -0.11 | 2.62 | -0.24 | 3.05 | 0.37 | 3.78 | -0.15 | 2.15 | 0.25 | 2.33 | 0.54 | 4.64 |
|  | ASIS - PSIS right | 0.31 | 2.29 | 0.03 | 1.63 | -0.42 | 2.95 | -0.25 | 2.67 | -0.35 | 2.93 | 0.29 | 1.86 |
|  | ASIS - symphysis left | -0.28 | 4.30 | 0.25 | 5.79 | 1.33 | 7.06 | -0.25 | 3.60 | 0.29 | 3.96 | -1.52 | 8.28 |
|  | ASIS - symphysis right | -1.02 | 8.52 | -0.52 | 5.43 | 2.41 | 6.61 | -0.30 | 4.04 | -1.62 | 9.67 | -1.25 | 8.23 |
|  | PSIS - symphysis left | -0.23 | 2.60 | -0.91 | 5.97 | -0.91 | 3.98 | 0.06 | 4.20 | 0.06 | 4.84 | 0.62 | 7.06 |
|  | PSIS - symphysis right | 7.01 | 60.74 | 0.50 | 4.50 | -0.71 | 4.46 | 0.22 | 4.19 | -0.15 | 4.60 | 0.41 | 6.88 |
|  | symphysis - S5 | -2.51 | 27.11 | -1.87 | 7.00 | -4.59 | 31.36 | 0.57 | 8.44 | 0.14 | 1.93 | 0.36 | 2.32 |
| angle | promontory - ASIS left | 0.00 | 0.08 | 0.01 | 0.11 | -2.05 | 7.94 | -1.46 | 9.18 | 0.01 | 0.07 | 0.00 | 0.06 |
| [°] | promontory - ASIS right | 0.11 | 3.86 | 1.92 | 5.39 | 1.14 | 18.32 | -0.22 | 10.95 | 0.68 | 5.13 | 0.52 | 3.55 |
|  |  |  |  |  |  |  |  |  |  |  |  |  |  |
| **SIJ** | |  |  |  |  |  |  |  |  |  |  |  |  |
| distance | S1 - S2 left | 0.33 | 3.64 | -0.18 | 5.41 | 1.30 | 5.10 | 0.24 | 7.16 | 0.68 | 5.22 | 0.88 | 6.40 |
| [mm] | S1 - S2 right | 0.16 | 3.45 | -0.55 | 2.70 | 0.49 | 2.55 | -1.12 | 4.13 | 0.81 | 5.46 | 0.29 | 3.54 |
|  | S2 - S3 left | -0.27 | 3.62 | -0.27 | 3.01 | 0.35 | 7.38 | 0.26 | 3.52 | 0.07 | 5.09 | -0.08 | 1.84 |
|  | S2 - S3 right | -0.39 | 2.11 | 0.07 | 2.81 | 0.36 | 2.75 | 0.24 | 2.62 | 0.32 | 3.18 | -0.10 | 1.62 |

*S1B:* Within-group comparison of the SIJ patient group and the control group measurements from two raters

|  |  | **No belt** | | | | **Moderate tension** | | | | **Maximum tension** | | | |
| --- | --- | --- | --- | --- | --- | --- | --- | --- | --- | --- | --- | --- | --- |
|  |  | **SIJ patients** | | **controls** | | **SIJ patients** | | **controls** | | **SIJ patients** | | **controls** | |
|  |  | **mean** | **bias** | **mean** | **bias** | **mean** | **bias** | **mean** | **bias** | **mean** | **bias** | **mean** | **bias** |
|  |  |  |  |  |  |  |  |  |  |  |  |  |  |
| **Pelvis** |  |  |  |  |  |  |  |  |  |  |  |  |  |
| distance | ASIS left - ASIS right | -0.60 | 3.64 | -0.27 | 3.38 | -0.93 | 3.48 | -0.05 | 38.82 | -0.34 | 4.49 | 0.22 | 4.45 |
| [mm] | PSIS left - PSIS right | 0.02 | 4.32 | -0.01 | 2.55 | 0.35 | 5.23 | 0.34 | 15.94 | 0.33 | 3.30 | 0.34 | 2.90 |
|  | symphysis left - right | -76.04 | 42.82 | -79.69 | 22.36 | -75.71 | 44.90 | -79.35 | 21.12 | -79.17 | 22.97 | -79.82 | 22.79 |
|  | ASIS - PSIS left | 0.48 | 7.53 | -0.04 | 1.89 | 0.43 | 4.86 | 0.47 | 11.59 | -0.05 | 3.37 | 0.51 | 2.29 |
|  | ASIS - PSIS right | 0.43 | 6.03 | 0.30 | 1.45 | 0.19 | 6.15 | 0.30 | 10.17 | -0.24 | 1.94 | 0.00 | 1.74 |
|  | ASIS - symphysis left | -0.06 | 6.18 | -0.28 | 3.73 | -0.32 | 4.61 | -0.61 | 26.14 | -0.26 | 7.74 | -0.32 | 6.07 |
|  | ASIS - symphysis right | -0.67 | 6.05 | -0.78 | 4.35 | -0.38 | 5.98 | -0.88 | 26.24 | 0.29 | 5.15 | -0.10 | 6.01 |
|  | PSIS - symphysis left | 0.70 | 5.17 | -0.54 | 4.10 | 0.22 | 3.07 | -0.15 | 16.27 | -0.48 | 3.47 | 0.39 | 3.59 |
|  | PSIS - symphysis right | -3.61 | 30.84 | 0.57 | 2.58 | -3.32 | 29.71 | 0.63 | 18.68 | 0.28 | 2.16 | 0.06 | 3.54 |
|  | symphysis - S5 | -4.17 | 19.97 | -0.50 | 4.89 | -2.50 | 14.38 | -0.05 | 27.45 | 1.67 | 15.46 | 0.45 | 4.11 |
| angle | promontory - ASIS left | 1.08 | 3.97 | 0.74 | 4.60 | 0.01 | 0.10 | 0.01 | 0.18 | -1.07 | 3.94 | -0.73 | 4.55 |
| [°] | promontory - ASIS right | 2.09 | 10.72 | 0.57 | 8.57 | 1.05 | 5.00 | 0.25 | 10.13 | -1.03 | 9.17 | -0.32 | 6.38 |
|  |  |  |  |  |  |  |  |  |  |  |  |  |  |
| **SIJ** |  |  |  |  |  |  |  |  |  |  |  |  |  |
| distance | S1 - S2 left | 2.31 | 6.60 | 0.16 | 5.81 | 2.05 | 5.53 | 0.19 | 5.70 | 0.95 | 4.69 | 0.91 | 6.38 |
| [mm] | S1 - S2 right | -0.02 | 3.86 | -0.25 | 3.54 | -0.23 | 4.26 | -0.21 | 4.11 | 0.13 | 2.61 | 0.10 | 2.32 |
|  | S2 - S3 left | -0.11 | 3.33 | -0.06 | 2.11 | 0.33 | 2.68 | -0.52 | 2.14 | 0.22 | 3.39 | -0.42 | 2.83 |
|  | S2 - S3 right | 0.54 | 2.83 | -0.26 | 2.63 | 0.29 | 3.10 | 0.38 | 1.37 | -0.32 | 1.97 | 0.62 | 2.82 |
