# Supplementary material for: Pelvic Belt Effects on Pelvic Morphometry, Muscle Activity and Body Balance in Patients with Sacroiliac Joint Dysfunction
Source: PLoS One. 2015 Mar 17;10(3):e0116739. doi: 10.1371/journal.pone.0116739 (PMC4364533; doi:10.1371/journal.pone.0116739)
Supplement: S2 Table — The p-values refer to the data given in Table 3. No significant differences were observed for the different conditions of pelvic belt tension. (DOCX) [file pone.0116739.s010.docx]

*S2 Table:*

Within-group comparison of MRI-based morphometry data of patients with sacroiliac joint (SIJ) pain and controls with and without pelvic belt application. The *p-values* refer to the data given in table 3. No significant differences were observed for the different conditions of pelvic belt tension.

| ***p-values*** | | **no belt : moderate tension : maximum tension** | |
| --- | --- | --- | --- |
|  |  |  |  |
|  |  | **SIJ patients** | **controls** |
|  |  |  |  |
| **Pelvis** |  |  |  |
| distance | ASIS left - ASIS right | *0.99* | *0.67* |
| [mm] | PSIS left - PSIS right | *0.99* | *0.75* |
|  | symphysis left - symphysis right | *0.55* | *0.37* |
|  | ASIS - PSIS left | *0.98* | *0.94* |
|  | ASIS - PSIS right | *0.98* | *0.94* |
|  | ASIS - symphysis left | *0.99* | *0.94* |
|  | ASIS - symphysis right | *0.99* | *0.63* |
|  | PSIS - symphysis left | *0.98* | *0.66* |
|  | PSIS - symphysis right | *0.66* | *0.96* |
|  | symphysis - S5 (lower edge) | *0.47* | *0.66* |
| angle | promontory - ASIS left | *0.14* | *0.06* |
| [°] | promontory - ASIS right | *0.52* | *1.00* |
|  |  |  |  |
| **SIJ** |  |  |  |
| distance | S1 - S2 left | *0.66* | *0.44* |
| [mm] | S1 - S2 right | *0.66* | *0.96* |
|  | S2 - S3 left | *0.84* | *0.90* |
|  | S2 - S3 right | *0.47* | *0.55* |
